# Autoimmune Addison's Disease as Part of the Autoimmune Polyglandular Syndrome Type 1: Historical Overview and Current Evidence

**DOI:** 10.3389/fimmu.2021.606860

**Published:** 2021-02-26

**Authors:** Roberto Perniola, Alessandra Fierabracci, Alberto Falorni

**Affiliations:** ^1^Department of Pediatrics-Neonatal Intensive Care, V. Fazzi Hospital, ASL LE, Lecce, Italy; ^2^Infectivology and Clinical Trials Research Department, Bambino Gesù Children's Hospital, IRCCS, Rome, Italy; ^3^Section of Internal Medicine and Endocrinological and Metabolic Sciences, Department of Medicine, University of Perugia, Perugia, Italy

**Keywords:** Addison's disease, autoimmune polyendocrinopathies, cytochrome P450 enzyme system, history, transcription factors

## Abstract

The autoimmune polyglandular syndrome type 1 (APS1) is caused by pathogenic variants of the autoimmune regulator (*AIRE*) gene, located in the chromosomal region 21q22.3. The related protein, AIRE, enhances thymic self-representation and immune self-tolerance by localization to chromatin and anchorage to multimolecular complexes involved in the initiation and post-initiation events of tissue-specific antigen-encoding gene transcription. Once synthesized, the self-antigens are presented to, and cause deletion of, the self-reactive thymocyte clones. The clinical diagnosis of APS1 is based on the classic triad idiopathic hypoparathyroidism (HPT)—chronic mucocutaneous candidiasis—autoimmune Addison's disease (AAD), though new criteria based on early non-endocrine manifestations have been proposed. HPT is in most cases the first endocrine component of the syndrome; however, APS1-associated AAD has received the most accurate biochemical, clinical, and immunological characterization. Here is a comprehensive review of the studies on APS1-associated AAD from initial case reports to the most recent scientific findings.

## Introduction

The term autoimmune polyglandular syndrome (APS) designates a heterogeneous group of diseases sharing a common fundamental characteristic—damage to more than one organ, essentially but not exclusively endocrine—caused by pathological processes identifiable as autoimmune (1, 2). APSs were classified in the early 1980s (3–5). APS type 1 (APS1), often referred to as autoimmune polyendocrinopathy-candidiasis-ectodermal dystrophy (APECED) to summarize clinical features, is a monogenic disease with an autosomal recessive inheritance (6, 7). Traditionally, at least two of the major components—chronic mucocutaneous candidiasis (CMC), idiopathic hypoparathyroidism (HPT), and autoimmune Addison's disease (AAD)—are required for clinical diagnosis (8–10). The diagnostic pathway of APS1 cases with uncommon presentation and course may take advantage of early non-triad components, such as urticaria-like eruptions, gastrointestinal dysfunction, and enamel hypoplasia, along with type-1 interferon (IFN) antibody assay, pending molecular confirmation (11–16).

In APS type 2 (APS2), or Schmidt's syndrome, AAD is associated with autoimmune thyroid disease (ATD), type-1 diabetes (T1D), or both (17–19). In contrast, ATD along with other autoimmune diseases, HPT and AAD excluded, identifies APS type 3 (APS3) (20). Worldwide medical literature is closely aligned with this classification (21–28). However, it has been suggested to merge non-monogenic APSs into a single disease entity (29), as they recognize a multifactorial genetic predisposition, mostly related to the major histocompatibility complex class-I and class-II (*MHCI* and *MHCII*, respectively) genes and their regulators (30–35).

Since AAD is the bridge between APS1 and APS2, the revision of its principles implies the treatment of these subjects (36–41). This work aims to review the characteristics of APS1-associated AAD, providing a historical overview and looking at the most recent results deriving from studies on the animal models of the disease. Due to the common embryogenesis of the adrenal cortex and gonads (42), AAD intersects the autoimmune events involving the gonads, especially in the form of autoimmune primary ovarian insufficiency (APOI) of the female sex.

## AIRE, APS1, and the Animal Models of Disease

### AIRE: Basic Properties and Functions

Autoimmune regulator (*AIRE*), the gene responsible for APS1, is found in the chromosomal region 21q22.3 (6, 7, 43–45). The murine homolog (*Aire*) lies on chromosome 10 (46–48). The pool of tissues in which the gene is transcribed must be fully delineated (49–51), but undoubtedly the highest degree of expression is in medullary thymic epithelial cells (mTECs); here, the protein (AIRE/Aire) forms distinct nuclear speckles and co-localizes with the microtubular cytoskeleton (52, 53).

Earlier studies proved that bipotent thymic epithelial progenitor cells (TEPCs) give rise to mTEC and cortical TEC (cTEC) compartments in the embryonic and early neonatal thymus (54–58). It was then shown that selected TEPC clusters differentiate into mTEC sublineage derailing from a predefined cTEC development program (59–63). In the post-natal thymus, bipotent TEPCs become progressively quiescent, and the replenishment of TEC compartments is supported by sublineage-restricted precursors (64–69).

Committed mTECs descend from the apical layer of the thymic anlage, marked by tight-junction claudins 3 and 4 (70), while the initial stages of maturation require lymphostromal “crosstalk” with early T-cell subsets (71–73). MHC^lo^CD80^lo^AIRE^−^ mTECs (mTECs^lo^) include not only precursors of the mature MHC^hi^CD80^hi^AIRE^+^ mTECs (mTECs^hi^), but also cortico-medullary junctional TECs (jTECs) that recruit positively selected thymocytes into the medulla (74–78). In turn, mTECs^hi^ have a rapid turnover and give rise to various post-AIRE subsets (79–81); these include corneocyte-like mTECs and thymic tuft cells, which play a presumed role in addressing cytokine responses (82–87).

AIRE contains a caspase-activation and recruitment domain (CARD), a nuclear localization signal (NLS), a SAND (for Sp100-AIRE-NucP41/75-Deaf-1) domain, and two plant-homeodomain zinc fingers (PHD1 and PHD2, respectively) (88, 89). Amino-terminal and middle regions perform auxiliary functions, such as oligomerization, pro-apoptosis, nuclear shuttling, and DNA binding (90–93). At the carboxyl-terminal end, PHD1 binds to the tail of unmethylated histone H3 by electrostatic complementarity, and PHD2 activates gene transcription (94–97). To perform this function, AIRE interacts with enzymes, such as DNA-topoisomerases (DNA-TOPs) and DNA-activated protein kinase (DNA-PK), which belong to the multimolecular complex involved in DNA break and repair by non-homologous end joining (98–100).

As demonstrated in the murine thymus, Aire and co-actors localize to long stretches of chromatin known as super-enhancers, which enclose the transcription start sites of most Aire-dependent genes (101). Initiation of gene transcription is made effective by AIRE-induced recruitment of the positive transcriptional elongation factor b (P-TEFb), which enables elongation and pre-mRNA splicing into mature mRNA by phosphorylation and release of the stalled RNA-polymerase II (102, 103).

Due to the above properties, AIRE plays a crucial role in promiscuous gene expression within the thymus; in other words, AIRE drives the ectopic expression of genes that encode for enzymes, hormones, receptors, structural proteins and other molecules acting as self-antigens and normally synthesized in a few tissues (104–107). Their presentation to thymocytes induces apoptosis and deletion of self-reactive clones, which prevent their spreading as mature T cells (108–111). However, AIRE controls only a part of these genes; furthermore, the expression of any single AIRE-dependent gene affects a small percentage of mTECs and follows a stochastic pattern (112, 113), though co-expression pools of overlapping and complementary gene sets have been established (114–116).

AIRE also promotes the generation of regulatory T cells (111, 117, 118). The result of self-reactive thymocytes (i.e., negative selection or switch to tolerogenic function) depends on mTEC subsets and AIRE availability (119–122), division of labor and interplay between antigen-presenting cells (123–127), and degree of affinity between self-antigens and T-cell receptors (128, 129).

For a careful dissection of the above-mentioned arguments, the authors suggest readers to refer recent reviews that have thoroughly analyzed TEC dynamics and functions (130, 131), the molecular properties of AIRE, and its role in self-tolerance (132–135).

### Clinical Picture of APS1 and the Animal Models of Disease

The clinical picture of APS1 is quite repetitive, although, as indicated, minor or uncommon entities may precede the main triad in a number of cases (15, 16). In other patients, severe forms of autoimmune hepatitis (136–139), lung disease (140, 141), and oral/esophageal carcinoma (142, 143) worsen the disease and progress into a life-threatening condition.

*In vitro* mutagenesis suggests that specific *AIRE* genotypes, particularly that of Iranian-Jewish patients with APS1, give rise to distinctive clinical features (144–147). Other peculiarities concern patients carrying mutated AIRE chains that co-localize with the wild-type protein and undermine the oligomeric structure in a dominant way. Incomplete penetrance, late-onset autoimmunity, and a milder phenotype characterize the clinical picture (148–151).

Additional genetic traits, with particular reference to the MHC alleles, can influence the APS1 phenotype so that in large APS1 patient cohorts most of the disease components mirror the HLA associations established for the general population (152). As an example, Finnish patients with APS1 have a high prevalence of T1D, while ATD is surprisingly common in those of Southern Italy (8, 153); actually, both observations reflect the MHC predispositions of the respective populations (154, 155).

Regarding APS1-associated AAD, no susceptibility related to MHCI and MHCII was initially reported, while HLA-DR3 and -DR4 were recognized to confer a significant risk for sporadic and APS2-associated forms (5, 156, 157). Subsequent analysis of larger patient cohorts contradicted the previous claim and revealed that patients with APS1 carrying the DRB1^*^03 allele have a significantly higher prevalence of AAD, while DRB1^*^04 appears to be more closely associated with alopecia (152). A number of other genes have been linked to non-APS1-associated AAD, but none of these play a role in APS1 phenotype (35). On the contrary, *AIRE* sequencing allows a correct classification of some AAD cases, which were previously framed within sporadic forms and actually being part of APS1 (158, 159).

The animal models of APS1 provide a formidable support for delineating AIRE-related self-tolerance mechanisms, but provide scant information, if any, on the characteristics of the disease components (108–111). This is because *Aire*-deficient (*Aire*^−/−^) mice exhibit different pictures than human APS1 (160, 161); typically, the exocrine glands are targets of autoimmunity, while the adrenal cortex, and other endocrine glands, shows little or no damage (108, 109, 111). Furthermore, the clinical picture of *Aire*^−/−^ mice is modulated by the strain background, supporting the idea that gene modifiers control patterns of autoimmunity to each organ (162–164).

More recently, *Aire*^−/−^ rats were engineered by zinc-finger nucleases; the animals exhibited various APS1-like ectodermal dystrophies, periportal lymphocyte infiltrates (with “piecemeal” necrosis) of the liver, and a broad spectrum of antibodies to self-antigens, type-1 IFNs included, but the endocrine glands were not significantly affected. In part contradicting the above findings, the reproductive capacity of the animals was impaired by the damage of testis Leydig cells (165).

Interestingly, the thymic expression of genes encoding for three enzymes of the cytochrome-P450 (CYP) family involved in the steroid pathway and electively targeted by adrenal autoimmunity in APS1 suggests differing and somewhat contrasting regulatory mechanisms between humans and mice. Murine mitochondrial cholesterol side-chain cleavage enzyme (CYP11A1, or CYPscc) and microsomal 21-hydroxylase (CYP21) have a strong and intermediate degree, respectively, of dependence on Aire, while the gene expression of another microsomal CYP enzyme, 17α-hydroxylase/17,20 lyase (CYP17), does not change significantly in murine *Aire*^−/−^ mTECs (86, 109, 166–168). Unexpectedly, the expression of the human corresponding genes is unrelated to that of *AIRE* in thymoma and HEK293 cells, underlining that dependence on AIRE follows a species-specific pattern (169–171).

## AAD and APOI as Parts of APS1

### Prevalence of APS1-Associated AAD and APOI

The prevalence of AAD in APS2 and APS3 is, by definition, 100 and 0%, respectively. Regarding the prevalence of AAD in APS1, it should be noted that, prior to the APS classification (3–5), patients with APS1 are either described in case reports or grouped together with subjects suffering from other diseases. Consequently, a comprehensive overview of these APS1 cases is missing. Thanks to the collection of hundreds of scientific articles covering the whole relevant literature and considering the utmost care to avoid counting each patient more than once; the Authors reviewed 282 certain or highly probable APS1 cases reported till 1980. Personal data and clinical course of each patient and related references are listed in Supplementary Material. Any further information can be requested by e-mail to the corresponding author.

As shown in Table 1, AAD has a prevalence of ~57%, with no gender preponderance. Moreover, the mean age of the patients at disease onset was comparable: just under 12 years in males, and just over 11 years in female patients. AAD, in the form of acute adrenal failure neither readily recognized nor effectively treated, was a significant cause of death, starting with the little girl whose clinical course and autopsy findings were detailed by Ostertag (172).

**Table 1:** Summary of data from 282 confirmed or highly probable cases of autoimmune polyglandular syndrome type 1 (APS1) reported up until 1980.

	**Whole casuistic**	**Male patients^a^**	**Female patients^a^**
APS1 cases	282	126	153
Age at last observation/death	14.9 (0–56)	15.1 (0–38)	15.0 (0–56)
AAD patients	162 (57.4%)	72 (57.1%)	88 (57.5%)
Age at AAD onset	11.3 (0–34)	11.8 (0–33)	11.1 (0–34)
Patients deceased	64 (22.7%)	33 (26.2%)	30 (19.6%)
Age at decease	10.0 (0–33)	8.6 (0–24)	11.6 (0–33)
Patents deceased by acute adrenal failure	26 (9.2%)	12 (9.5%)	14 (9.2%)
APOI patients^b^	—	—	32/88 (36.4%)
Primary amenorrhea	—	—	21/88 (23.9%)
Secondary amenorrhea	—	—	11/88 (12.5%)

*Prevalence of autoimmune Addison's disease (AAD) and autoimmune primary ovarian insufficiency (APOI) is specified. Personal data and medical history of each patient are detailed in Supplementary Material*.

a *Gender was not specified in three (two Addisonian) patients*.

b *Calculated on female patients aged ≥13*.

Among Finnish patients with APS1, who represent a reference cohort in terms of number, genetic homogeneity, data centralization, and serial updates (8, 173–180), AAD reaches an incidence of 84%. To retrieve the data, Finnish researchers estimate the onset of each APS1 component over fixed age ranges, assuming that all patients live up to 50 years old. As of the 2006 update, no AAD cases were reported up to a patient age of 2 years, while AAD rate increased to 8, 40, and 65% in the age ranges 2–5, 5–10, and 10–15, respectively (180).

Ferre et al. calculated the incidence of APS1 components in American (mostly the US) patients in the same way and obtained consistent percentages for AAD (16). Again, AAD had a prevalence of 73 and 67% in two major reviews of 41 and 112 patients with APS1 from Northern Italy and Russia, respectively (9, 10).

In Table 2, the prevalence of AAD and APOI in various APS1 patient cohorts is reported, with the specification of their ethnic backgrounds (15, 144, 153, 181–197). AAD prevalence can sometimes be influenced by the mean patient age, for example, in Saudi patients with APS1 described by Bin-Abbas et al. (193). Conversely, the extent of follow-up suggests that the low prevalence of AAD in Iranian-Jewish patients with APS1 does not recognize an age-related factor (144).

**Table 2:** Prevalence of autoimmune Addison's disease (AAD) and autoimmune primary ovarian insufficiency (APOI) in cohorts of autoimmune polyglandular syndrome type-1 (APS1) patients, with specification of their ethnic background.

	***N***	**Mean age**			**AAD**			**Mean age at AAD onset**			**APOI^a^**	**Mean age at APOI onset**
		**Total**	**M**	**F**	**Total**	**M**	**F**	**Total**	**M**	**F**	**F**	**F**
Wagman et al. (181) Canadian patients	16	20.6	22.4	19.7	9/16	3/5	6/11	nr	nr	nr	2/7	nr
Zlotogora and Shapiro (144) Iranian-Jewish patients	23	nr	nr	nr	5/23	2/11	3/12	21.8	21.0	22.3	1/9	32.0
Wang et al. (182) US patients	20	nr	nr	nr	19/20	8/8	11/12	nr	nr	nr	nr	—
Cihakova et al. (183) Central-Eastern European patients	27	nr	nr	nr	24/27	nr	nr	nr	nr	nr	nr	—
Stolarski et al. (184) Polish patients	16	21.4	19.6	22.3	8/16	3/5	5/11	8.9	9.3	8.6	4/10	16.0
Dominguez et al. (185) Irish patients	31	19.9	20.2	19.6	21/31	8/13	13/18	nr	nr	nr	11/16	nr
Adamson et al. (186)UK patients	33	24.2	24.9	23.1	24/33	19/24	5/9	nr	nr	nr	3/8	nr
Wolff et al. (187) Norwegian patients	34	33.8	35.2	32.0	23/34	13/19	10/15	12.3	11.8	12.9	7/13	17.0
Trebušak Podkrajšek et al. (188) Dutch, Eastern European patients	11	16.2	14.7	18.0	10/11	5/6	5/5	9.0	9.2	8.8	3/4	14.3
Perniola et al. (153) Southern Italian patients	12	26.7	28.7	24.7	9/12	5/6	4/6	8.3	10.8	5.2	5/6	19.6
Zaidi et al. (189) Indian patients	9	15.1	23.5	12.7	7/9	2/2	5/7	10.9	15.0	9.2	1/4	nr
Proust-Lemoine et al. (190) North-Western French patients	19	31.2	28.2	36.4	15/19	10/12	5/7	12.6	11.3	15.2	5/7	18.8
Tóth et al. (191) Hungarian patients	7	15.1	14.0	16.7	4/7	1/4	3/3	nr	nr	nr	1/2	nr
Orlova et al. (192) Russian patients	46	nr	nr	nr	30/46	nr	nr	9.9	nr	nr	nr	—
Bin-Abbas et al. (193) Saudi patients	20	10.9	9.4	12.2	8/20	3/9	5/11	7.5	6.7	8.0	1/4	15.0
Mazza et al. (15) Northern Italian patients	24	16.5	nr	nr	15/24	nr	nr	8.0	nr	nr	nr	—
Meloni et al. (194) Sardinian patients	22	25.8	28.1	24.3	15/22	5/9	10/13	10.0	10.3	9.8	8/10	24.5
Bratanic et al. (195) Slovenian patients	15	24.5	26.9	17.7	8/15	6/11	2/4	8.8	9.7	5.9	2/3	16.3
Fierabracci et al. (196) Turkish patients	23	17.5	18.3	17.0	14/23	4/7	7/12	15.0	16.2	14.2	2/8	15.0
Weiler et al. (197) Brazilian patients	14	18.7	15.5	19.5	10/14	3/4	7/10	nr	nr	nr	5/7	nr

a *Calculated on female patients aged ≥13*.

*nr, not reported*.

Closely related to the mechanisms involved in AAD, a large percentage of patients with APS1 suffer from primary hypogonadism, with a privileged gender association. APS1 women have been included in APOI studies (198, 199), and APS1 has become a well-known condition causing this disease (200–218). Recently, the clinical and immunological features of APS1-associated APOI have been detailed in the Finnish cohort of APS1 women (219).

As shown in Table 2, APOI approaches or exceeds AAD prevalence in some APS1 patient cohorts. Surprisingly, Adamson et al. reported that 6 out of 19 UK APS1 male patients had gonadal insufficiency, but the criteria adopted to satisfy this diagnosis were not reported (186). Similar findings were outlined in Slovenian patients with APS1, but again the diagnostic criteria were not specified (195).

### Clinical History and Particular Issues

Irvine and Barnes observed a bimodal age distribution at AAD onset; the first peak emerged at the end of the first decade and involved the majority of HPT patients. A later peak (fifth decade) mainly affected women with T1D, ATD, or both (220). In subsequent years, long-term observation of large AAD cohorts confirmed that the disease onset occurs at an early age only in patients with APS1 (221–227). Conversely, more recent nationwide AAD studies have purposely excluded patients with APS1, emphasizing the difference between monogenic and multifactorial pathogenesis (228, 229).

Furthermore, the prediction of AAD onset in patients with APS1 with related humoral autoimmunity has contributed to characterize the biochemical and clinical stages of the disease. Damage typically begins in the zona glomerulosa and causes impaired mineralocorticoid secretion and increased plasma renin activity. The subsequent involvement of the zona fasciculata has been divided into three stages of hypocortisolism: subnormal cortisol response after adrenocorticotropic-hormone (ACTH) stimulation test, persistent ACTH increase, and decrease in basal cortisol level, respectively (230–235).

Replacement therapy is unable to reproduce the natural hormone pulse, so the treatment of AAD is a challenge in itself and carries risks of suboptimal or excessive dosage (236). In APS1, the problem is accentuated by the coexistence of other hormonal deficits, with particular reference to HPT and ATD. Untreated AAD masks the early stages of HPT, as the hypercalciuric and hypocalcemic effects of glucocorticoids wear off. By the same principle, AAD replacement therapy can induce hypocalcemic seizures in patients with APS1 with subclinical HPT (237, 238). Fortunately, HPT treatment mitigates the negative impact of glucocorticoids on bone health (239).

Again, it is important to remember that, regardless of the underlying disease, untreated AAD can cause reversible thyroid dysfunction (240–242). Co-occurrence of AAD and ATD should caution when initiating thyroid replacement therapy due to the risk of raising the basal metabolic rate and precipitating an adrenal crisis.

Patients with APS1 have been included in AAD therapeutic trials (243–245), and special attention is paid to them in consensus statements on diagnosis, treatment, and follow-up (246).

## APS1-Associated AAD and APOI: Humoral Immunity

Although in statistical terms AAD is the third component of APS1 triad, it has received the best immunological characterization; it is probably accepted that APS1 studies have made a substantial contribution to the delineation of the pathogenetic and immunological aspects of AAD.

### The Founding Studies

Antibodies to adrenal cortex (AC-Abs) were first demonstrated by complement fixation (CF) in sera from patients with sporadic or APS2-associated AAD (247). Sera from unselected patients with APS1 were included in subsequent studies using both CF and indirect immunofluorescence (IIF) (248–255). The cell cytoplasm of the adrenal layers was stained in positive samples (248); the mitochondrial and microsomal fractions of the tissue extracts retained the antigenic properties, since pre-absorption with them inhibited the reaction (249, 253). In addition, AAD sera from the patients with associated HPT, CMC, or both contained other antibody specificities, such as those to thyroid (248, 249, 252, 254), salivary gland ducts (249), gastric parietal cells and intrinsic factor (250, 252, 254), parathyroid glands (251, 255), and liver (252).

In parallel studies, the same methods were used to test for antibodies to steroid-producing cells (StC-Abs). Five out of 77 AAD sera reacted against granulosa and theca interna cells of Graafian follicles, luteal cells, ovarian interstitial cells, Leydig cells, and placental syncytiotrophoblasts; three of these sera belonged to APS1 women with coexistent APOI (256, 257). A similar result was obtained with serum from a male patient with APS1 (258). Moreover, StC-Ab-positive sera from patients with APS1 showed *in vitro* cytotoxicity on granulosa cells of the Graafian follicle (259). The above-mentioned results were confirmed by subsequent studies (260–262).

Pre-absorption with adrenal and gonadal extracts reduced or abolished the antibody titer, leading researchers to infer that the self-antigens shared by the adrenal cortex and steroid-producing extra-adrenal tissues were related to steroid pathway enzymes (256–258, 262).

Antibodies to germline cells were also detected in some patients with APS1 (256, 257, 260, 263), following the studies of Vallotton and Forbes, who found them in patients with gonadal dysgenesis (264–266).

Unlike Anglo-American patients in the 1970s, Finnish patients with APS1 had already been grouped into a distinct disease entity called moniliasis-polyendocrinopathy syndrome; precipitating AC-Abs were detected in the serum of these patients by gel diffusion (267, 268). The association appeared to be restricted to APS1-associated AAD (269). Two specific adrenal antigens were targeted, one named P (particulate) and precipitating in the mitochondrial fraction, and the other named S (soluble) since it was present in all subcellular fractions; the latter contained a variety of determinants, partly common to human sera and sera from other species (270, 271).

### Antibodies to CYP Enzymes

In the mid-1980s, genes encoding for the above-mentioned CYP enzymes were identified and cloned (272–274). CYP21 was soon identified as the major self-antigen target of adult-onset, either sporadic or APS2-associated AAD; generally, AC-Abs were searched for by IIF; the assay was followed by Western blot (WB) on tissue fractions separated by gel electrophoresis (275–277). Data were confirmed by immunoprecipitation (IP) of ^35^S-labeled-cell lysates from human adrenal cells (275) and by the reaction of human antibodies to recombinant CYP21 (CYP21-Abs) expressed in *Saccharomyces cerevisiae* (276, 277).

In contrast, the definition of adrenal autoimmunity in APS1 was delayed by contradicting data. For the first time, CYP17 was identified as the self-antigen recognized by precipitating AC-Abs of APS1 sera (278). In another study, while CYP21-Abs were found in sera from non-APS1 AAD patients as indicated, the serum from the only patient with APS1 stained testis Leydig cells and targeted CYPscc (275). Further analysis of APS1 sera confirmed these results (279, 280). Thus, APS1 sera appeared to react against either CYP17 or CYPscc, while CYP21 was thought to represent an exclusive target of autoimmunity in sporadic and APS2-associated AAD (281, 282).

Finally, Uibo et al., using WB on *Escherichia coli* lysates, which expressed recombinant CYP fragments, stated that all three enzymes are self-antigens in APS1 (283). Furthermore, the absorption studies were congruent with tissue distribution: since CYP21 is restricted to the adrenal cortex, CYP17 is also represented in the gonads, and the ubiquitous CYPscc is also found in the placenta, only incubation with adrenal homogenates could completely abolish reactivity. Ovarian and testis homogenates absorbed reactivity against CYPScc and CYP17, while placental homogenates selectively absorbed that against CYPScc (281). Several subsequent studies have confirmed the strong association (60–100% of cases) of adrenal autoimmunity, any form of AAD included, with AC-Abs and CYP21-Abs (284–293).

In contrast, StC-Abs and antibodies to CYP17 and CYPscc (CYP17-Abs and CYPscc-Abs, respectively) can be found in a limited number (5–42%) of sporadic or APS2-associated AAD cases and mainly identified in APOI women (286, 287, 292–295). As shown in Table 3, their prevalence is significantly higher in patients with APS1 (16, 152, 183, 187, 194, 283, 287, 296–298).

**Table 3:** Fraction of sera positive for antibodies to adrenal cortex (AC-Abs), steroid-producing cells (StC-Abs), cytochrome-P450 21-hydroxylase (CYP21-Abs), cytochrome-P450 cholesterol side-chain cleavage enzyme (CYPscc-Abs), and cytochrome-P450 17α-hydroxylase/17,20 lyase (CYP17-Abs) in cohorts of autoimmune polyglandular syndrome type-1 (APS1) patients, with specification of their ethnic background.

	***N***	**AC-Abs**	**StC-Abs**	**CYP21-Abs**	**CYPscc-Abs**	**CYP17-Abs**
Uibo et al. (283) Finnish patients	50	nd	nd	16/50	22/50	16/50
Chen et al. (287) Northern Italian patients	11	8/11	5/11	7/11	5/11	6/11
Perniola et al. (296) Southern Italian patients	10	10/10	10/10	8/10	9/10	5/10
Myhre et al. (297) Norwegian patients	20	nd	nd	13/20	7/20	5/20
Cihakova et al. (183) Central-Eastern European patients	18	nd	nd	8/18	11/18	12/18
Halonen et al. (152) Finnish, Scandinavian patients	60	nd	nd	38/60	38/60	29/60
Söderbergh et al. (298) Finnish, Scandinavian patients	90	nd	nd	59/90	47/90	40/90
Wolff et al. (187) Norwegian patients	29	nd	nd	20/29	12/29	6/29
Meloni et al. (194) Sardinian patients	13	nd	nd	10/13	12/13	11/13
Ferre et al. (16) American patients	35	nd	nd	18/35	24/35	nd

*nd, not determined*.

The same results have been achieved starting from the opposite point of view, i.e., extrapolating from patients with various autoimmune diseases, precisely those with gonadal insufficiency. Furthermore, gonadal autoimmunity markers prevail in APS1 and APS2 APOI women, while the percentage of sera positive for StC-Abs and CYPscc-Abs/CYP17-Abs falls in women with non-AAD-associated APOI, and in patients with other autoimmune diseases (299–302).

Lastly, in a minority of APS1 sera, it is possible to find antibodies against members of other steroid pathways; this is the case of antibodies to an enzyme belonging to the hydroxysteroid dehydrogenase (HSD) family, namely 3βHSD (303).

### Concordance Between IIF and Antibodies to CYP Enzymes

Comparing IIF, a tool normally available in laboratory medicine, and the assays used to detect antibodies to CYP enzymes, which are generally reserved for experimental settings, presumably contains the most useful information for clinical purposes. Due to the selective synthesis of CYP21 in the adrenal cortex, AC-Abs and CYP21-Abs have a close relationship that produces very high correlation coefficients, regardless of the analytical methods (284, 285, 289, 290, 292, 293).

Progressive depletion of self-antigen source causes antibody titers to show an inverse correlation with disease duration (285, 291). However, the sensitivity of the analytical methods makes CYP21-Abs more reliable than AC-Abs in long-lasting AAD (more than 15–20 years of duration) (288). In other studies, CYP21-Abs neither correlate with AAD duration (286) nor are they prevalent over AC-Abs (293).

In relation to the tissue distribution of CYPs, StC-Abs, routinely tested on ovary and testis specimens, are associated with positivity for CYPscc-Abs, CYP17-Abs, or both; again, this relationship does not differ among the subcohorts of AAD patients (287, 293).

### Predictive Role of AC-Abs, StC-Abs, and Antibodies to CYP Enzymes

In most cases, AC-Abs and CYP21-Abs anticipate the onset of AAD, and their predictive role is higher in patients with APS1 (233, 304). The same is true for StC-Abs, though the overlap of AC-Abs and StC-Abs creates a phenomenon of statistical redundancy in univariate analyses (233).

The above-mentioned results are more relevant when adding patients with other diseases. An Italian research group followed adult and pediatric patients suffering from various autoimmune diseases and divided according to AC-Ab positivity: while only three patients with APS1 were included in the group of adult subjects (one only being AC-Ab-positive and acquiring overt AAD at follow-up), all but one AC-Ab-positive children enrolled in the latter group were patients with APS1 (305, 306).

In a reappraisal of these studies, the same research group observed that, among the parameters at highest risk of acquiring overt AAD in AC-Ab-positive patients, was an impaired adrenal function at enrollment, the simultaneous presence of idiopathic HPT, CMC, or both, pediatric age, and a high titer of AC-Abs and CYP21-Abs. In particular, the cumulative risk of developing AAD at 11 years of age was 100% in patients with APS1, a much higher percentage than in patients with other autoimmune and non-autoimmune diseases (307, 308).

On the other hand, StC-Abs play a prominent role in predicting APOI in APS1 women, but the statistical power is slightly lower than that achieved by AC-Abs (and StC-Abs themselves) in predicting AAD (231). Interestingly, measurement of AC-Abs and CYP21-Abs in women with sporadic APOI may be important in identifying patients at risk of acquiring overt AAD (299).

### Epitope Targeting

A first determination of the epitopes recognized by CYP21-Abs tested only sporadic and APS2-associated AAD sera (309). A subsequent study showed that the middle region of CYP21 (CYP21_164−356_) retained antigenic sites, and sera from patients with APS1 reacted not unlike those from patients with other AAD forms (310). The use of CYP21 fragments and mouse monoclonal antibodies emphasized the antigenic relevance of the middle and carboxyl-terminal regions of CYP21 and resulted in the detection of two short amino-acid stretches (CYP21_335−339_ and CYP21_406−411_) as the main epitopes independently from patient disease (sporadic AAD, APS1, APS2, and isolated CYP21-Ab positivity) (311, 312). The same is true for CYPscc epitopes (313).

Due to the similarity between the antigenic targets, it was suggested that CYP17-Abs and CYP21-Abs have immunological cross-reactivity (314), but subsequent studies in sera from patients with APS1 disproved this hypothesis (315, 316).

Finally, it has been shown that antibodies to CYP enzymes belong mainly to the IgG1 subclass in patients with APS1, indicating a predominant Th1 response (317). However, IgG4 isotype specificity identifies a small subset of patients with Th2-oriented response (318).

### Other Antibody Specificities and Proteomics

Adrenal antibody specificities other than those related to steroid pathways have been occasionally described in patients with APS1: Kendall-Taylor et al. found IgG-class antibodies that antagonized ACTH action in an APS1 girl with AAD (319).

The view is somewhat richer if we look at the gonadal self-antigens; testis-specific protein 10 (TSGA10) was identified by immunoscreening of testis and pituitary cDNA expression library with sera from patients with APS1, but no clinical phenotype correlated with antibody positivity (320, 321).

In turn, based on the observation that the maternal antigen that the embryo requires (Mater) acts as an ovarian target of autoimmunity in an early thymectomy mouse model (322, 323), Brozzetti et al. searched for antibodies to the leucine-rich repeat protein 5 (NALP5), which represents the corresponding human self-antigen. Interestingly, antibody positivity involved the majority of patients with APS1 and included a number of patients with non-APS1-associated AAD, APOI, or both (324).

Currently, proteomics appears to be capable of capturing the enormous biodiversity of potential targets and offers an undisputable advantage over previous candidate-based approaches (325). Landegren et al. used sera from patients with APS1 to probe proteome arrays containing thousands of full-length human proteins. Together with established autoimmunity targets, they detected melanoma-associated antigen B2 (MAGEB2) and disulfide isomerase-like protein of the testis (PDILT) as gonadal self-antigens (326).

More recently, Vazquez et al., employing a high-throughput, proteome-wide phage display method, identified some novel self-antigens targeted by APS1 sera. No adrenal targets were included but, in turn, antibodies to the ovarian KH-domain-containing 3-like protein (KHDC3L), which form an oocyte-specific critical complex with NALP5, were involved in APS1-associated APOI (327).

## APS1-Associated AAD: Cellular Immunity

Although antibodies to CYP enzymes play a prominent role in indicating the nature, antigenic targets and, especially in the APS1-associated form, the time interval before the clinical onset of AAD, it is well-known that cellular immunity is responsible for organ damage (328). Even circulating T-lymphocytes show clear signs of activation in the early stage of the disease (16, 185, 329–333). Further evidence is provided by the (rare) animal models of autoimmune adrenalitis (334).

Findings from patients with APS1 strongly support this belief—starting from the cited study of Ostertag (172), autopsy samples of patients with Addisonian APS1 show massive infiltration of the adrenal cortex by mononuclear cells; with time, gross atrophy of the gland is evident, characterized by the replacement of the adrenal layers with connective tissue. The medulla is usually spared (335–340). As expected, a variable degree of adrenal inflammation is frequently found in patients with APS1 who had not yet reached a symptomatic stage, an uncommon feature of autopsy, if present, in the general population (341).

Interestingly, the delineation of CYP21 epitopes benefited from interferon-γ (IFNγ, a type-2 IFN) assay in cultures of peripheral blood mononuclear cells challenged with several CYP21 fragments: some patients with APS1 carrying the MHCI HLA-B35 allele were included in these studies. A CYP21_431−450_ fragment stimulated lymphocytes from non-APS1 AAD patients, while those from two APS1 siblings reacted against CYP_131−150_. Lymphocytes from the third patient with APS1 did not proliferate, presumably due to long-lasting AAD (342, 343).

## Conclusions

Several key issues need to be clarified in the relationship between the loss of self-tolerance resulting from AIRE/Aire deficiency and related organ failures. In particular, the non-dependence on AIRE of the main adrenal self-antigens in the human thymus raises questions about the intrinsic mechanisms that trigger autoimmunity in APS1. Dissection of these aspects can help plan suitable strategies for causal therapy.

Meanwhile, a look at the history of APS1 and APS1-associated AAD confirms the challenge that such disease implies—interpolation between AAD and other endocrine failures, mainly HPT and ATD, amplifies the risks and benefits of replacement therapies and highlights the need for proper use of mineralo- and glucocorticoids.

Above all, correct management of specific laboratory tests can identify early markers of adrenal impairment and avoid pitfalls and dangers deriving from the onset and stabilization of one of the most insidious APS1 components.

## Author Contributions

RP revised APS1 case reports and cohort studies, detailed AIRE properties and drew up the manuscript. AFi described APS1 clinical picture and examined the genotype/phenotype relationship. AFa dissected the immunological aspects of APS1. All authors contributed to the article and approved the submitted version.

## Conflict of Interest

The authors declare that the research was conducted in the absence of any commercial or financial relationships that could be construed as a potential conflict of interest.

## Abbreviations

AAD: autoimmune Addison's disease
AC-Abs: adrenal-cortex antibodies
ACTH: adrenocorticotropic hormone
AIRE/Aire: autoimmune regulator
APECED: autoimmune polyendocrinopathy-candidiasis-ectodermal dystrophy
APOI: autoimmune primary ovarian insufficiency
APS: autoimmune polyglandular syndrome
ATD: autoimmune thyroid disease
CARD: caspase-activation and recruitment domain
CMC: chronic mucocutaneous candidiasis
CF: complement fixation
CYP: cytochrome P450
CYP17: CYP 17α-hydroxylase/17,20 lyase
CYP17-Abs: antibodies to CYP17
CYP21: CYP 21-hydroxylase
CYP21-Abs: antibodies to CYP21
CYP11A1/CYPscc: CYP cholesterol side-chain cleavage enzyme
CYPscc-Abs: antibodies to CYPscc
DNA-PK: DNA-activated protein kinase
DNA-TOP: DNA topoisomerase
HPT: hypoparathyroidism
HSD: hydroxysteroid dehydrogenase
IFN: interferon
IIF: indirect immunofluorescence
IP: immunoprecipitation
KHDC3L: KH-domain-containing 3-like protein
MAGEB2: melanoma-associated antigen B2
Mater: maternal antigen that embryo requires
MHC: major histocompatibility complex
NALP5: leucine-rich-repeat protein 5
NLS: nuclear localization signal
PDILT: disulfide isomerase-like protein of the testis
PHD: plant-homeodomain (zinc finger)
P-TEFb: positive transcriptional elongation factor b
SAND: Sp100-AIRE-NucP41/75-Deaf-1
StC-Abs: steroid-cell antibodies
T1D: type-1 diabetes
TEC: thymic epithelial cell
TEPC: thymic epithelial progenitor cell
TSGA10: testis-specific protein 10
WB: Western blot.

## References

[1] Witebsky E Rose NR Terplan K Paine JR Egan RW. Chronic thyroiditis and autoimmunization. J Am Med Assoc. (1957) 164:1439–47. 10.1001/jama.1957.02980130015004 13448890

[2] Rose NR Bona C. Defining criteria for autoimmune diseases (Witebsky's postulates revisited). Immunol Today. (1993) 14:426–30. 10.1016/0167-5699(93)90244-F 8216719

[3] Neufeld M Maclaren N Blizzard R. Autoimmune polyglandular syndromes. Pediatr Ann. (1980) 9:154–62. 6990358

[4] Neufeld M Blizzard RM. Polyglandular autoimmune disease. Proc Serono Symp. (1980) 33:357–65.

[5] Neufeld M Maclaren NK Blizzard RM. Two types of autoimmune Addison's disease associated with different polyglandular autoimmune (PGA) syndromes. Medicine (Baltimore). (1981) 60:355–62. 10.1097/00005792-198109000-00003 7024719

[6] Nagamine K Peterson P Scott HS Kudoh J Minoshima S Heino M. Positional cloning of the APECED gene. Nat Genet. (1997) 17:393–8. 10.1038/ng1297-393 9398839

[7] Finnish-German APECED Consortium. An autoimmune disease, APECED, caused by mutations in a novel gene featuring two PHD-type zinc-finger domains. Nat Genet. (1997) 17:399–403. 10.1038/ng1297-399 9398840

[8] Ahonen P Myllärniemi S Sipilä I Perheentupa J. Clinical variation of autoimmune polyendocrinopathy-candidiasis-ectodermal dystrophy (APECED) in a series of 68 patients. N Engl J Med. (1990) 322:1829–36. 10.1056/NEJM199006283222601 2348835

[9] Betterle C Greggio NA Volpato M. Autoimmune polyglandular syndrome type 1. J Clin Endocrinol Metab. (1998) 83:1049–55. 10.1210/jcem.83.4.4682 9543115

[10] Orlova EM Sozaeva LS Kareva MA Oftedal BE Wolff ASB Breivik L. Expanding the phenotypic and genotypic landscape of autoimmune polyendocrine syndrome type 1. J Clin Endocrinol Metab. (2017) 102:3546–56. 10.1210/jc.2017-00139 28911151

[11] Buzi F Badolato R Mazza C Giliani S Notarangelo LD Radetti G. Autoimmune polyendocrinopathy-candidiasis-ectodermal dystrophy syndrome: time to review diagnostic criteria? J Clin Endocrinol Metab. (2003) 88:3146–8. 10.1210/jc.2002-021495 12843157

[12] Meager A Visvalingam K Peterson P Möll K Murumägi A Krohn K. Anti-interferon autoantibodies in autoimmune polyendocrinopathy syndrome type 1. PLoS Med. (2006) 3:1152–64. 10.1371/journal.pmed.0030289 16784312 PMC1475653

[13] Oftedal BE Bøe Wolff AS Bratland E Kämpe O Perheentupa J Myhre AG. Radioimmunoassay for autoantibodies against interferon omega; its use in the diagnosis of autoimmune polyendocrine syndrome type I. Clin Immunol. (2008) 129:163–9. 10.1016/j.clim.2008.07.002 18708298

[14] Meloni A Furcas M Cetani F Marcocci C Falorni A Perniola R. Autoantibodies against type I interferons as an additional diagnostic criterion for autoimmune polyendocrine syndrome type I. J Clin Endocrinol Metab. (2008) 93:4389–97. 10.1210/jc.2008-0935 18728167

[15] Mazza C Buzi F Ortolani F Vitali A Natarangelo LD Weber G. Clinical heterogeneity and diagnostic delay of autoimmune polyendocrinopathy-candidiasis-ectodermal dystrophy syndrome. Clin Immunol. (2011) 139:6–11. 10.1016/j.clim.2010.12.021 21295522

[16] Ferre EMN Rose SR Rosenzweig SD Burbelo PD Romito KR Niemela JE. Redefined clinical features and diagnostic criteria in autoimmune polyendocrinopathy-candidiasis-ectodermal dystrophy. JCI Insight. (2016) 1:e88782. 10.1172/jci.insight.88782 27588307 PMC5004733

[17] Bennett IL Jr Harvey AM Carpenter CCJ Solomon N Silberberg SG Bledsoe T. Schmidt's syndrome (thyroid and adrenal insufficiency): a review of the literature and a report of fifteen new cases including ten instances of coexistent diabetes mellitus. Trans Am Clin Climatol Assoc. (1964) 75:27–36. 14132718

[18] Lechuga-Gomez EE Meyerson J Bigazzi PE Walfish PG. Polyglandular autoimmune syndrome type II. CMAJ. (1988) 138:632–4. 3281739 PMC1267743

[19] Betterle C Volpato M Greggio AN Presotto F. Type 2 polyglandular autoimmune disease (Schmidt's syndrome). J Pediatr Endocrinol Metab. (1996) 9(S1):113–23. 10.1515/jpem.1996.9.s1.113 8887161

[20] Betterle C Zanchetta R. Update on autoimmune polyendocrine syndromes (APS). Acta Biomed. (2003) 74:9–33. 12817789

[21] Trence DL Morley JE Handwerger BS. Polyglandular autoimmune syndromes. Am J Med. (1984) 77:107–16. 10.1016/0002-9343(84)90444-3 6377888

[22] Meyerson J Lechuga-Gomez EE Bigazzi PE Walfish PG. Polyglandular autoimmune syndrome: current concepts. CMAJ. (1988) 138:605–12. 3281737 PMC1267738

[23] Riley WJ. Autoimmune polyglandular syndromes. Horm Res. (1992) 38:9–15. 10.1159/000182585 1292989

[24] Obermayer-Straub P Manns MP. Autoimmune polyglandular syndromes. Baillieres Clin Gastroenterol. (1998) 12:293–315. 10.1016/s0950-3528(98)90136-1 9890074

[25] Eisenbarth GS Gottlieb PA. Autoimmune Polyendocrine Syndromes. N Engl J Med. (2004) 350:2068–79. 10.1056/NEJMra030158 15141045

[26] Kahaly GJ. Polyglandular autoimmune syndromes. Eur J Endocrinol. (2009) 161:11–20. 10.1530/EJE-09-0044 19411300

[27] Michels AW Gottlieb PA. Autoimmune polyglandular syndromes. Nat Rev Endocrinol. (2010) 6:270–7. 10.1038/nrendo.2010.40 20309000

[28] Frommer L Kahaly GJ. Autoimmune polyendocrinopathy. J Clin Endocrinol Metab. (2019) 104:4769–82. 10.1210/jc.2019-00602 31127843

[29] Husebye ES Anderson MS Kämpe O. Autoimmune polyendocrine syndromes. N Engl J Med. (2018) 378:2543–4. 10.1056/NEJMc1805308 29949487

[30] Eisenbarth G Wilson P Ward F Lebovitz HE. HLA type and occurrence of disease in familial polyglandular failure. N Engl J Med. (1978) 298:92–4. 10.1056/NEJM197801122980209 619238

[31] Farid NR Larsen B Payne R Noel EP Sampson L. Polyglandular autoimmune disease and HLA. Tissue Antigens. (1980) 16:23–9. 10.1111/j.1399-0039.1980.tb00284.x 7008251

[32] Muir A She JX. Advances in the genetics and immunology of autoimmune polyglandular syndrome II/III and their clinical applications. Ann Med Interne (Paris). (1999) 150:301–12. 10519018

[33] Robles DT Fain PR Gottlieb PA Eisenbarth GS. The genetics of autoimmune polyendocrine syndrome type II. Endocrinol Metab Clin North Am. (2002) 31:353–68. 10.1016/s0889-8529(01)00015-9 12092455

[34] Dittmar M Kahaly GJ. Polyglandular autoimmune syndromes: immunogenetics and long-term follow-up. J Clin Endocrinol Metab. (2003) 88:2983–92. 10.1210/jc.2002-021845 12843130

[35] Falorni A Brozzetti A Perniola R. From genetic predisposition to molecular mechanisms of autoimmune primary adrenal insufficiency. Front Horm Res. (2016) 46:115–32. 10.1159/000443871 27211051

[36] Riley WJ Maclaren NK. Autoimmune adrenal disease. In: Laron Z, editor. Pediatric and Adolescent Endocrinology. Vol. 13: Adrenal Diseases in Childhood. Basel: Karger (1984). p. 162–72.

[37] Muir A Schatz DA Maclaren NK. Autoimmune Addison's disease. Springer Semin Immunopathol. (1993) 14:275–84. 10.1007/BF00195978 8438210

[38] Vaidya B Pearce S Kendall-Taylor P. Recent advances in the molecular genetics of congenital and acquired primary adrenocortical failure. Clin Endocrinol (Oxf). (2000) 53:403–18. 10.1046/j.1365-2265.2000.01116.x 11012564

[39] Peterson P Uibo R Krohn KJE. Adrenal autoimmunity: results and developments. Trends Endocrinol Metab. (2000) 11:285–90. 10.1016/s1043-2760(00)00283-6 10920386

[40] Betterle C Dal Pra C Mantero F Zanchetta R. Autoimmune adrenal insufficiency and autoimmune polyendocrine syndromes: autoantibodies, autoantigens, and their applicability in diagnosis and disease prediction. Endocr Rev. (2002) 23:327–64. 10.1210/edrv.23.3.0466 12050123

[41] Arlt W Allolio B. Adrenal insufficiency. Lancet. (2003) 361:1881–93. 10.1016/S0140-6736(03)13492-7 12788587

[42] Hatano O Takakusu A Nomura M Morohashi K. Identical origin of adrenal cortex and gonad revealed by expression profiles of Ad4BP/SF-1. Genes Cells. (1996) 1:663–71. 10.1046/j.1365-2443.1996.00254.x 9078392

[43] Aaltonen J Björses P Sandkuijl L Perheentupa J Peltonen L. An autosomal locus causing autoimmune disease: autoimmune polyglandular disease type I assigned to chromosome 21. Nat Genet. (1994) 8:83–7. 10.1038/ng0994-83 7987397

[44] Björses P Aaltonen J Vikman A Perheentupa J Ben-Zion G Chiumello G. Genetic homogeneity of autoimmune polyglandular disease type I. Am J Hum Genet. (1996) 59:879–86. 8808604 PMC1914815

[45] Aaltonen J Horelli-Kuitunen N Fan JB Björses P Perheentupa J Myers R. High-resolution physical and trascriptional mapping of the autoimmune polyendocrinopathy-candidiasis-ectodermal dystrophy locus on chromosome 21q22.3 by FISH. Genome Res. (1997) 7:820–9. 10.1101/gr.7.8.820 9267805

[46] Mittaz L Rossier C Heino M Peterson P Krohn KJE Gos A. Isolations and characterization of the mouse *Aire* gene. Biochem Biophys Res Commun. (1999) 255:483–90. 10.1006/bbrc.1999.0223 10049735

[47] Blechschmidt K Schweiger M Wertz K Poulson R Christensen HM Rosenthal A. The mouse *Aire* gene: comparative genomic sequencing, gene organization, and expression. Genome Res. (1999) 9:158–66. 10.1101/gr.9.2.158 10022980 PMC310712

[48] Wang CY Shi JD Davoodi-Semiromi A She JX. Cloning of *Aire*, the mouse homologue of the autoimmune regulator (*AIRE*) gene responsible for autoimmune polyglandular syndrome type 1 (APS1). Genomics. (1999) 55:322–6. 10.1006/geno.1998.5656 10049587

[49] Eldershaw SA Sansom DM Narendran P. Expression and function of the autoimmune regulator (Aire) gene in non-thymic tissue. Clin Exp Immunol. (2011) 163:296–308. 10.1111/j.1365-2249.2010.04316.x 21303359 PMC3048612

[50] Perniola R. Expression of the autoimmune regulator gene and its relevance to the mechanisms of central and peripheral tolerance. Clin Dev Immunol. (2012) 2012:e207403. 10.1155/2012/207403 23125865 PMC3485510

[51] Zhao B Chang L Fu H Sun G Yang W. The role of autoimmune regulator (AIRE) in peripheral tolerance. J Immunol Res. (2018) 2018:e3930750. 10.1155/2018/3930750 30255105 PMC6142728

[52] Heino M Peterson P Kudoh J Nagamine K Lagerstedt A Ovod V. Autoimmune regulator is expressed in the cells regulating immune tolerance in thymus medulla. Biochem Biophys Res Commun. (1999) 257:821–5. 10.1006/bbrc.1999.0308 10208866

[53] Heino M Peterson P Sillanpää N Guérin S Wu L Anderson G. RNA and protein expression of the murine autoimmune regulator gene (Aire) in normal, RelB-deficient and in NOD mouse. Eur J Immunol. (2000) 30:1884–93. 10.1002/1521-4141(200007)30:7<1884::AID-IMMU1884>3.0.CO;2-P 10940877

[54] Bennett AR Farley A Blair NF Gordon J Sharp L Blackburn CC. Identification and characterization of thymic epithelial progenitor cells. Immunity. (2002) 16:803–14. 10.1016/S1074-7613(02)00321-728 12121662

[55] Gill J Malin M Holländer GA Boyd R. Generation of a complete thymic micro-environment by MTS24^+^ epithelial cells. Nat Immunol. (2002) 3:635–42. 10.1038/ni812 12068292

[56] Rossi SW Jenkinson WE Anderson G Jenkinson EJ. Clonal analysis reveals a common progenitor for thymic cortical and medullary epithelium. Nature. (2006) 441:988–91. 10.1038/nature04813 16791197

[57] Bleul CC Corbeaux T Reuter A Fisch P Mönting JS Boehm T. Formation of a functional thymus initiated by a postnatal epithelial progenitor cell. Nature. (2006) 441:992–6. 10.1038/nature04850 16791198

[58] Rossi SW Chidgey AP Parnell SM Jenkinson WE Scott HS Boyd RL. Redefining epithelial progenitor potential in the developing thymus. Eur J Immunol. (2007) 37:2411–8. 10.1002/eji.200737275 17694573

[59] Shakib S Desanti GE Jenkinson WE Parnell SM Jenkinson EJ Anderson G. Checkpoints in the development of thymic cortical epithelial cells. J Immunol. (2009) 182:130–7. 10.4049/jimmunol.182.1.130 19109143

[60] Baik S Jenkinson EJ Lane PJL Anderson G Jenkinson WE. Generation of both cortical and Aire^+^ medullary thymic epithelial compartments from CD205^+^ progenitors. Eur J Immunol. (2013) 43:589–94. 10.1002/eji.201243209 23299414 PMC3960635

[61] Ohigashi I Zuklys S Sakata M Mayer CE Zhanybekova S Murata S. Aire-expressing thymic medullary epithelial cells originate from β5t-expressing progenitor cells. Proc Natl Acad Sci USA. (2013) 110:9885–90. 10.1073/pnas.1301799110 23720310 PMC3683726

[62] Mayer CE Žuklys S Zhanybekova S Ohigashi I Teh HY Sansom SN. Dynamic spatio-temporal contribution of single β5t^+^ cortical epithelial precursors to the thymus medulla. Eur J Immunol. (2016) 46:846–56. 10.1002/eji.201545995 26694097 PMC4832341

[63] Alves NL Takahama Y Ohigashi I Ribeiro AR Baik S Anderson G. Serial progression of cortical and medullary thymic epithelial microenvironments. Eur J Immunol. (2014) 44:16–22. 10.1002/eji.201344110 24214487 PMC4253091

[64] Wong K Lister NL Barsanti M Lim JMC Hammett MV Khong DM. Multilineage potential and self-renewal define an epithelial progenitor cell population in the adult thymus. Cell Rep. (2014) 8:1198–209. 10.1016/j.celrep.2014.07.029 25131206

[65] Ucar A Ucar O Klug P Matt S Brunk F Hofmann TG. Adult thymus contains FoxN1^−^ epithelial stem cells that are bipotent for medullary and cortical thymic epithelial lineages. Immunity. (2014) 41:257–69. 10.1016/j.immuni.2014.07.005 25148026 PMC4148705

[66] Ohigashi I Zuklys S Sakata M Mayer CE Hamazaki Y Minato N. Adult thymic medullary epithelium is maintained and regenerated by lineage-restricted cells rather than bipotent progenitors. Cell Rep. (2015) 13:1432–43. 10.1016/j.celrep.2015.10.012 26549457

[67] Ulyanchenko S O'Neill KE Medley T Farley AM Vaidya HJ Cook AM. Identification of a bipotent epithelial progenitor population in the adult thymus. Cell Rep. (2016) 14:2819–32. 10.1016/j.celrep.2016.02.080 26997270 PMC4819909

[68] Dumont-Lagacé M Gerbe H Daouda T Laverdure JP Brochu S Lemieux S. Detection of quiescent radioresistant epithelial progenitors in the adult thymus. Front Immunol. (2017) 8:e1717. 10.3389/fimmu.2017.01717 29259606 PMC5723310

[69] Lepletier A Hun ML Hammett MV Wong K Naeem H Hedger M. Interplay between Follistatin, Activin A, and BMP4 signaling regulates postnatal thymic epithelial progenitor cell differentiation during aging. Cell Rep. (2019) 27:3887–901. 10.1016/j.celrep.2019.05.045 31242421

[70] Hamazaki Y Fujita H Kobayashi T Choi Y Scott HS Matsumoto M. Medullary thymic epithelial cells expressing Aire represent a unique lineage derived from cells expressing claudin. Nat Immunol. (2007) 8:304–11. 10.1038/ni1438 17277780

[71] Rossi SW Kim MY Leibbrandt A Parnell SM Jenkinson WE Glanville SH. RANK signals from CD4^+^3^−^ inducer cells regulate development of Aire-expressing epithelial cells in the thymic medulla. J Exp Med. (2007) 204:1267–72. 10.1084/jem.20062497 17502664 PMC2118623

[72] Roberts NA White AJ Jenkinson WE Turchinovich G Nakamura K Withers DR. Rank signaling links the development of invariant γδ T cell progenitors and Aire^+^ medullary epithelium. Immunity. (2012) 36:427–37. 10.1016/j.immuni.2012.01.016 22425250 PMC3368267

[73] Lopes N Sergé A Ferrier P Irla M. Thymic crosstalk coordinates medulla organization and T-cell tolerance induction. Front Immunol. (2015) 6:e365. 10.3389/fimmu.2015.00365 26257733 PMC4507079

[74] Kurobe H Liu C Ueno T Saito F Ohigashi I Seach N. CCR7-dependent cortex-to-medulla migration of positively selected thymocytes is essential for establishing central tolerance. Immunity. (2006) 24:165–77. 10.1016/j.immuni.2005.12.011 16473829

[75] Lkhagvasuren E Sakata M Ohigashi I Takahama Y. Lymphotoxin β receptor regulates the development of CCL21-expressing subset of postnatal medullary thymic epithelial cells. J Immunol. (2013) 190:5110–7. 10.4049/jimmunol.120320 23585674

[76] Onder L Nindl V Scandella E Chai Q Cheng HW Caviezel-Firner S. Alternative NF-κB signaling regulates mTEC differentiation from podoplanin-expressing precursors in the cortico-medullary junction. Eur J Immunol. (2015) 45:2218–31. 10.1002/eji.201545677 25973789

[77] Kozai M Kubo Y Katakai T Kondo H Kiyonari H Schaeuble K. Essential role of CCL21 in establishment of central self-tolerance in T cells. J Exp Med. (2017) 214:1925–35. 10.1084/jem.20161864 28611158 PMC5502431

[78] Miragaia RJ Zhang X Gomes T Svensson V Ilicic T Henriksson J. Single-cell RNA-sequencing resolves self-antigen expression during mTEC development. Sci Rep. (2018) 8:e685. 10.1038/s41598-017-19100-4 29330484 PMC5766627

[79] Gray DHD Seach N Ueno T Milton MK Liston A Lew AM. Developmental kinetics, turnover, and stimulatory capacity of thymic epithelial cells. Blood. (2006) 108:3777–85. 10.1182/blood-2006-02-004531 16896157

[80] Gray D Abramson J Benoist C Mathis D. Proliferative arrest and rapid turnover of thymic epithelial cells expressing Aire. J Exp Med. (2007) 204:2521–8. 10.1084/jem.20070795 17908938 PMC2118482

[81] Colomé N Collado J Bech-Serra JJ Liiv I Antón LC Peterson P. Increased apoptosis after autoimmune regulator expression in epithelial cells revealed by a combined quantitative proteomics approach. J Proteome Res. (2010) 9:2600–9. 10.1021/pr100044d 20218732

[82] Yano M Kuroda N Han H Meguro-Horike M Nishikawa Y Kiyonari H. Aire controls the differentiation program of thymic epithelial cells in the medulla for the establishment of self-tolerance. J Exp Med. (2008) 205:2827–38. 10.1084/jem.20080046 19015306 PMC2585853

[83] White AJ Nakamura K Jenkinson WE Saini M Sinclair C Seddon B. Lymphotoxin signals from positively selected thymocytes regulate the terminal differentiation of medullary thymic epithelial cells. J Immunol. (2010) 185:4769–76. 10.4049/jimmunol.1002151 20861360 PMC3826119

[84] Wang X Laan M Bichele R Kisand K Scott HS Peterson P. Post-Aire maturation of thymic medullary epithelial cells involves selective expression of keratinocyte-specific autoantigens. Front Immunol. (2012) 3:e19. 10.3389/fimmu.2012.00019 22448160 PMC3310317

[85] Michel C Miller CN Küchler R Brors B Anderson MS Kyewski B. Revisiting the road map of medullary thymic epithelial cell differentiation. J Immunol. (2017) 199:3488–503. 10.4049/jimmunol.1700203 28993517

[86] Bornstein C Nevo S Giladi A Kadouri N Pouzolles M Gerbe F. Single-cell mapping of the thymic stroma identifies IL-25-producing tuft epithelial cells. Nature. (2018) 559:622–6. 10.1038/s41586-018-0346-1 30022162

[87] Miller CN Proekt I von Moltke J Wells KL Rajpurkar AR Wang H. Thymic tuft cells promote an IL-4-enriched medulla and shape thymocyte development. Nature. (2018) 559:627–31. 10.1038/s41586-018-0345-2 30022164 PMC6062473

[88] Björses P Aaltonen J Horelli-Kuitunen N Yaspo ML Peltonen L. Gene defect behind APECED: a new clue to autoimmunity. Hum Mol Genet. (1998) 7:1547–53. 10.1093/hmg/7.10.1547 9735375

[89] Peterson P Nagamine K Scott H Heino M Kudoh J Shimizu N. APECED: a monogenic autoimmune disease providing new clues to self-tolerance. Immunol Today. (1998) 19:384–6. 10.1016/S0167-5699(98)01293-6 9745199

[90] Pitkänen J Doucas V Sternsdorf T Nakajima T Aratani S Jensen K. The autoimmune regulator protein has transcriptional transactivating properties and interacts with the common coactivator CREB-binding protein. J Biol Chem. (2000) 275:16802–9. 10.1074/jbc.M908944199 10748110

[91] Ferguson BJ Alexander C Rossi SW Liiv I Rebane A Worth CL. AIRE's CARD revealed, a new structure for central tolerance provokes transcriptional plasticity. J Biol Chem. (2008) 283:1723–31. 10.1074/jbc.M707211200 17974569

[92] Ilmarinen T Melén K Kangas H Julkunen I Ulmanen I Eskelin P. The monopartite nuclear localization signal of autoimmune regulator mediates its nuclear import and interaction with multiple importin α molecules. FEBS J. (2006) 273:315–24. 10.1111/j.1742-4658.2005.05065.x 16403019

[93] Gibson TJ Ramu C Gemünd C Aasland R. The APECED polyglandular autoimmune syndrome protein, AIRE-1, contains the SAND domain and is probably a transcription factor. Trends Biochem Sci. (1998) 23:242–4. 10.1016/S0968-0004(98)01231-6 9697411

[94] Org T Chignola F Hetényi C Gaetani M Rebane A Liiv I. The autoimmune regulator PHD finger binds to non-methylated histone H3K4 to activate gene expression. EMBO Rep. (2008) 9:370–6. 10.1038/sj.embor.2008.11 18292755 PMC2261226

[95] Koh AS Kuo AJ Park SY Cheung P Abramson J Bua D. Aire employs a histone-binding module to mediate immunological tolerance, linking chromatin regulation with organ-specific autoimmunity. Proc Natl Acad Sci USA. (2008) 105:15878–83. 10.1073/pnas.0808470105 18840680 PMC2572939

[96] Gaetani M Matafora V Saare M Spiliotopoulos D Mollica L Quilici G. AIRE-PHD fingers are structural hubs to maintain the integrity of chromatin-associated interactome. Nucleic Acids Res. (2012) 40:11756–68. 10.1093/nar/gks933 23074189 PMC3526288

[97] Yang S Bansal K Lopes J Benoist C Mathis D. Aire's plant homeodomain(PHD)-2 is critical for induction of immunological tolerance. Proc Natl Acad Sci USA. (2013) 110:1833–8. 10.1073/pnas.1222023110 23319629 PMC3562810

[98] Liiv I Rebane A Org T Saare M Maslovskaja J Kisand K. DNA-PK contributes to the phosphorylation of AIRE: importance in transcriptional activity. Biochim Biophys Acta. (2008) 1783:74–83. 10.1016/j.bbamcr.2007.09.003 17997173 PMC2225445

[99] Abramson J Giraud M Benoist C Mathis D. Aire's partners in the molecular control of immunological tolerance. Cell. (2010) 140:123–35. 10.1016/j.cell.2009.12.030 20085707

[100] Guha M Saare M Maslovskaja J Kisand K Liiv I Haljasorg U. DNA breaks and chromatin structural changes enhance the transcription of autoimmune regulator target genes. J Biol Chem. (2017) 292:6542–54. 10.1074/jbc.M116.764704 28242760 PMC5399106

[101] Bansal K Yoshida H Benoist C Mathis D. The transcriptional regulator Aire binds to and activates super-enhancers. Nat Immunol. (2017) 18:263–73. 10.1038/ni.3675 28135252 PMC5310976

[102] Oven I Brdičková N Kohoutec J Vaupotič T Narat M Peterlin BM. AIRE recruits P-TEFb for transcriptional elongation of target genes in medullary thymic epithelial cells. Mol Cell Biol. (2007) 27:8815–23. 10.1128/MCB.01085-07 17938200 PMC2169392

[103] Giraud M Yoshida H Abramson J Rahl PB Young RA Mathis D. Aire unleashes stalled mRNA polymerase to induce ectopic gene expression in thymic epithelial cells. Proc Natl Acad Sci USA. (2012) 109:535–40. 10.1073/pnas.1119351109 22203960 PMC3258631

[104] Derbinski J Schulte A Kyewski B Klein L. Promiscuous gene expression in medullary thymic epithelial cells mirrors the peripheral self. Nat Immunol. (2001) 2:1032–9. 10.1038/ni723 11600886

[105] Kyewski B Derbinski J Gotter J Klein L. Promiscuous gene expression and central T-cell tolerance: more than meets the eye. Trends Immunol. (2002) 23:364–71. 10.1016/S1471-4906(02)02248-2 12103357

[106] Kyewski B Derbinski J. Self-representation in the thymus: an extended view. Nat Rev Immunol. (2004) 4:688–98. 10.1038/nri1436 15343368

[107] Kyewski B Klein L. A central role for central tolerance. Annu Rev Immunol. (2006) 24:571–606. 10.1146/annurev.immunol.23.021704.115601 16551260

[108] Ramsey C Winqvist O Puhakka L Halonen M Moro A Kämpe O. Aire deficient mice develop multiple features of APECED phenotype and show altered immune response. Hum Mol Genet. (2002) 11:397–409. 10.1093/hmg/11.4.397 11854172

[109] Anderson MS Venanzi ES Klein L Chen Z Berzins SP Turley SJ. Projection of an immunological self shadow within the thymus by the Aire protein. Science. (2002) 298:1395–401. 10.1126/science.1075958 12376594

[110] Liston A Lesage S Wilson J Peltonen L Goodnow CC. Aire regulates negative selection of organ-specific T cells. Nat Immunol. (2003) 4:350–4. 10.1038/ni906 12612579

[111] Kuroda N Mitani T Takeda N Ishimaru N Arakaki R Hayashi Y. Development of autoimmunity against transcriptionally unrepressed target antigen in the thymus of Aire-deficient mice. J Immunol. (2005) 174:1862–70. 10.4049/jimmunol.174.4.1862 15699112

[112] Derbinski J Pinto S Rösch S Hexel K Kyewski B. Promiscuous gene expression patterns in single medullary thymic epithelial cells argue for a stochastic mechanism. Proc Natl Acad Sci USA. (2008) 105:657–62. 10.1073/pnas.0707486105 18180458 PMC2206592

[113] Villaseñor J Besse W Benoist C Mathis D. Ectopic expression of peripheral-tissue antigens in the thymic epithelium: probabilistic, monoallelic, misinitiated. Proc Natl Acad Sci USA. (2008) 105:15854–9. 10.1073/pnas.0808069105 18836079 PMC2572966

[114] Pinto S Michel C Schmidt-Glenewinkel H Harder N Rohr K Wild S. Overlapping gene coexpression patterns in human medullary thymic epithelial cells generate self-antigen diversity. Proc Natl Acad Sci USA. (2013) 110:E3497–505. 10.1073/pnas.1308311110 23980163 PMC3773787

[115] Brennecke P Reyes A Pinto S Rattay K Nguyen M Küchler R. Single-cell transcriptome analysis reveals coordinated ectopic gene-expression patterns in medullary thymic epithelial cells. Nat Immunol. (2015) 16:933–41. 10.1038/ni.3246 26237553 PMC4675844

[116] Meredith M Zemmour D Mathis D Benoist C. Aire controls gene expression in the thymic epithelium with ordered stochasticity. Nat Immunol. (2015) 16:942–9. 10.1038/ni.3247 26237550 PMC4632529

[117] Anderson MS Venanzi ES Chen Z Berzins SP Benoist C Mathis D. The cellular mechanism of Aire control of T cell tolerance. Immunity. (2005) 23:227–39. 10.1016/j.immuni.2005.07.005 16111640

[118] Chen Z Benoist C Mathis D. How defects in central tolerance impinge on a deficiency in regulatory T cells. Proc Natl Acad Sci USA. (2005) 102:14735–40. 10.1073/pnas.0507014102 16203996 PMC1253589

[119] Aschenbrenner K D'Cruz LM Vollmann EH Hinterberger M Emmerich J Swee LK. Selection of Foxp3^+^ regulatory T cells specific for self antigen expressed and presented by Aire^+^ medullary thymic epithelial cells. Nat Immunol. (2007) 8:351–8. 10.1038/ni1444 17322887

[120] Aichinger M Wu C Nedjic J Klein L. Macroautophagy substrates are loaded onto MHC class II of medullary thymic epithelial cells for central tolerance. J Exp Med. (2013) 210:287–300. 10.1084/jem.20122149 23382543 PMC3570095

[121] Mouri Y Ueda Y Yamano T Matsumoto M Tsuneyama K Kinashi T. Mode of tolerance induction and requirement for Aire are governed by the cell types that express self-antigen and those that present antigen. J Immunol. (2017) 199:3959–71. 10.4049/jimmunol.1700892 29101311

[122] Lebel MÈ Coutelier M Galipeau M Kleinman CL Moon JJ Melichar HJ. Differential expression of tissue-restricted antigens among mTEC is associated with distinct autoreactive T cell fates. Nat Commun. (2020) 11:e3734. 10.1038/s41467-020-17544-3 32709894 PMC7381629

[123] Hubert FX Kinkel SA Davey GM Phipson B Mueller SN Liston A. Aire regulates the transfer of antigen from mTECs to dendritic cells for induction of thymic tolerance. Blood. (2011) 118:2462–72. 10.1182/blood-2010-06-286393 21505196

[124] Taniguchi RT DeVoss JJ Moon JJ Sidney J Sette A Jenkins MK. Detection of an autoreactive T-cell population within the polyclonal repertoire that undergoes distinct autoimmune regulator (Aire)-mediated selection. Proc Natl Acad Sci USA. (2012) 109:7847–52. 10.1073/pnas.1120607109 22552229 PMC3356674

[125] Perry JSA Lio CWJ Kau AL Nutsch K Yang Z Gordon JI. Distinct contributions of Aire and antigen-presenting-cell subsets to the generation of self-tolerance in the thymus. Immunity. (2014) 41:414–26. 10.1016/j.immuni.2014.08.007 25220213 PMC4175925

[126] Skogberg G Lundberg V Berglund M Gudmundsdottir J Telemo E Lindgren S. Human thymic epithelial primary cells produce exosomes carrying tissue-restricted antigens. Immunol Cell Biol. (2015) 93:727–34. 10.1038/icb.2015.33 25776846 PMC4575951

[127] Lancaster JN Thyagarajan HM Srinivasan J Li Y Hu Z Ehrlich LIR. Live-cell imaging reveals the relative contributions of antigen-presenting cell subsets to thymic central tolerance. Nat Commun. (2019) 10:e2220. 10.1038/s41467-019-09727-4 31101805 PMC6525199

[128] Wirnsberger G Mair F Klein L. Regulatory T cell differentiation of thymocytes does not require a dedicated antigen-presenting cell but is under T cell-intrinsic developmental control. Proc Natl Acad Sci USA. (2009) 106:10278–83. 10.1073/pnas.0901877106 19515822 PMC2694684

[129] Hinterberger M Aichinger M Prazeres da Costa O Voehringer D Hoffmann R Klein L. Autonomous role of medullary thymic epithelial cells in central CD4^+^ T cell tolerance. Nat Immunol. (2010) 11:512–20. 10.1038/ni.1874 20431619

[130] Abramson J Anderson G. Thymic epithelial cells. Annu Rev Immunol. (2017) 26:85–118. 10.1146/annurev-immunol-051116-052320 28226225

[131] Kadouri N Nevo S Goldfarb Y Abramson J. Thymic epithelial cell heterogeneity: TEC by TEC. Nat Rev Immunol. (2020) 20:239–53. 10.1038/s41577-019-0238-0 31804611

[132] Perniola R Musco G. The biophysical and biochemical properties of the autoimmune regulator (AIRE) protein. Biochim Biophys Acta. (2014) 1842:326–37. 10.1016/j.bbadis.2013.11.020 24275490

[133] Proekt I Miller CN Lionakis MS Anderson MS. Insights into immune tolerance from AIRE deficiency. Curr Opin Immunol. (2017) 49:71–8. 10.1016/j.coi.2017.10.003 29065385 PMC5705335

[134] Perniola R. Twenty years of AIRE. Front Immunol. (2018) 9:e98. 10.3389/fimmu.2018.00098 PMC5816566 29483906

[135] Passos GA Speck-Hernandez CA Assis AF Mendes-da-Cruz DA. Update on *Aire* and thymic negative selection. Immunology. (2018) 153:10–20. 10.1111/imm.12831 28871661 PMC5721245

[136] Michele TM Fleckenstein J Sgrignoli AR Thuluvath PJ. Chronic active hepatitis in the type I polyglandular autoimmune syndrome. Postgrad Med J. (1994) 70:128–31. 10.1136/pgmj.70.820.128 8170886 PMC2397651

[137] Gebre-Medhin G Husebye ES Gustafsson J Winqvist O Goksøyr A Rorsman F. Cytochrome P450IA2 and aromatic l-amino acid decarboxylase are hepatic autoantigens in autoimmune polyendocrine syndrome type I. FEBS Lett. (1997) 412:439–45. 10.1016/s0014-5793(97)00797-7 9276443

[138] Clemente MG Obermayer-Straub P Meloni A Strassburg CP Arangino V Tukey RH. Cytochrome P450 1A2 is a hepatic autoantigen in autoimmune polyglandular syndrome type 1. J Clin Endocrinol Metab. (1997) 82:1353–61. 10.1210/jcem.82.5.3913 9141515

[139] Clemente MG Meloni A Obermayer-Straub P Frau F Manns MP De Virgiliis S. Two cytochromes P450 are major hepatocellular autoantigens in autoimmune polyglandular syndrome type 1. Gastroenterology. (1998) 114:324–8. 10.1016/s0016-5085(98)70484-6 9453493

[140] Alimohammadi M Dubois N Sköldberg F Hallgren Å Tardivel I Hedstrand H. Pulmonary autoimmunity as a feature of autoimmune polyendocrine syndrome type 1 and identification of KCNRG as a bronchial autoantigen. Proc Natl Acad Sci USA. (2009) 106:4396–401. 10.1073/pnas.0809986106 19251657 PMC2648890

[141] Shum AK Alimohammadi M Tan CL Cheng MH Metzger TC Law CS. BPIFB1 is a lung-specific autoantigen associated with interstitial lung disease. Sci Transl Med. (2013) 5:e206ra139. 10.1126/scitranslmed.3006998 24107778 PMC3882146

[142] Rautemaa R Hietanen J Niissalo S Pirinen S Perheentupa J. Oral and oesophageal squamous cell carcinoma – a complication or component of autoimmune polyendocrinopathy-candidiasis-ectodermal dystrophy (APECED, APS-I). Oral Oncol. (2007) 43:607–13. 10.1016/j.oraloncology.2006.07.005 16997613

[143] Böckle BC Wilhelm M Müller H Götsch C Sepp NT. Oral mucous squamous cell carcinoma – an anticipated consequence of autoimmune polyendocrinopathy-candidiasis-ectodermal dystrophy (APECED). J Am Acad Dermatol. (2010) 62:864–8. 10.1016/j.jaad.2009.06.061 20304522

[144] Zlotogora J Shapiro MS. Polyglandular autoimmune syndrome type I among Iranian Jews. J Med Genet. (1992) 29:824–6. 10.1136/jmg.29.11.824 1453436 PMC1016181

[145] Björses P Halonen M Palvimo JJ Kolmer M Aaltonen J Ellonen P. Mutations in the AIRE gene: effects on subcellular location and transactivation function of the autoimmune polyendocrinopathy-candidiasis-ectodermal dystrophy protein. Am J Hum Genet. (2000) 66:378–92. 10.1086/302765 10677297 PMC1288090

[146] Ramsey C Bukrinsky A Peltonen L. Systematic mutagenesis of the functional domains of AIRE reveals their role in intracellular targeting. Hum Mol Genet. (2002) 11:3299–308. 10.1093/hmg/11.26.3299 12471056

[147] Halonen M Kangas H Rüppell T Ilmarinen T Ollila J Kolmer M. APECED-causing mutations in AIRE reveal the functional domains of the protein. Hum Mutat. (2004) 23:245–57. 10.1002/humu.20003 14974083

[148] Cetani F Barbesino G Borsari S Pardi E Cianferotti L Pinchera A. A novel mutation of the autoimmune regulator gene in an Italian kindred with autoimmune polyendocrinopathy-candidiasis-ectodermal dystrophy, acting in a dominant fashion and strongly cosegregating with hypothyroid autoimmune thyroiditis. J Clin Endocrinol Metab. (2001) 86:4747–52. 10.1210/jcem.86.10.7884 11600535

[149] Bellacchio E Palma A Corrente C Di Girolamo F Kemp EH Di Matteo G. The possible implication of the S250C variant of the autoimmune regulator protein in a patient with autoimmunity and immunodeficiency: *in silico* analysis suggests a molecular pathogenic mechanism for the variant. Gene. (2014) 549:286–94. 10.1016/j.gene.2014.07.064 25068407

[150] Oftedal BE Hellesen A Erichsen MM Bratland E Vardi A Perheentupa J. Dominant mutations in the autoimmune regulator AIRE are associated with common organ-specific autoimmune diseases. Immunity. (2015) 42:1185–96. 10.1016/j.immuni.2015.04.021 26084028

[151] Abbott JK Huoh YS Reynolds PR Yu L Rewers M Reddy M. Dominant-negative loss of function arises from a second, more frequent variant within the SAND domain of autoimmune regulator (AIRE). J Autoimmun. (2018) 88:114–20. 10.1016/j.jaut.2017.10.010 29129473 PMC5846191

[152] Halonen M Eskelin P Myhre AG Perheentupa J Husebye ES Kämpe O. AIRE mutations and human leukocyte antigen genotypes as determinants of the autoimmune polyendocrinopathy-candidiasis-ectodermal dystrophy phenotype. J Clin Endocrinol Metab. (2002) 87:2568–74. 10.1210/jcem.87.6.8564 12050215

[153] Perniola R Filograna O Greco G Pellegrino V. High prevalence of thyroid autoimmunity in Apulian patients with autoimmune polyglandular syndrome type 1. Thyroid. (2008) 18:1027–9. 10.1089/thy.2008.0027 18713028

[154] Tuomilehto J Karvonen M Pitkäniemi J Virtala E Kohtamäki K Toivanen L. Record-high incidence of Type I (insulin-dependent) diabetes mellitus in Finnish children. Diabetologia. (1999) 42:655–60. 10.1007/s001250051212 10382584

[155] Svejgaard A Morling N Platz P Ryder LP Thomsen M. HLA and disease associations with special reference to mechanisms. Transplant Proc. (1981) 13:913–7. 7022961

[156] Maclaren NK Riley WJ. Inherited susceptibility to autoimmune Addison's disease is linked to human leukocyte antigens-DR3 and/or DR4, except when associated with type I autoimmune polyglandular syndrome. J Clin Endocrinol Metab. (1986) 62:455–9. 10.1210/jcem-62-3-455 3484749

[157] Huang W Connor E Dela Rosa T Muir A Schatz D Silverstein J. Although DR3-DQB1^*^0201 may be associated with multiple component diseases of the autoimmune polyglandular syndromes, the human leukocyte antigen DR4-DQB1^*^0302 haplotype is implicated only in β-cell autoimmunity. J Clin Endocrinol Metab. (1996) 81:2559–63. 10.1210/jcem.81.7.8675578 8675578

[158] Bøe AS Knappskog PM Myhre AG Sørheim JI Husebye ES. Mutational analysis of the autoimmune regulator (AIRE) gene in sporadic autoimmune Addison's disease can reveal patients with unidentified autoimmune polyendocrine syndrome type I. Eur J Endocrinol. (2002) 146:519–22. 10.1530/eje.0.1460519 11916620

[159] Eriksson D Bianchi M Landegren N Dalin F Skov J Hultin-Rosenberg L. Common genetic variation in the autoimmune regulator (*AIRE*) locus is associated with autoimmune Addison's disease in Sweden. Sci Rep. (2018) 8:e8395. 10.1038/s41598-018-26842-2 29849176 PMC5976627

[160] Kekäläinen E Miettinen A Arstila TP. Does the deficiency of Aire in mice really resemble human APECED? Nat Rev Immunol. (2007) 7:1. 10.1038/nri2136-c1 17932495

[161] Hubert FX Kinkel SA Crewther PE Cannon PZF Webster KE Link M. Aire-deficient C57BL/6 mice mimicking the common human 13-base pair deletion mutant present with only a mild autoimmune phenotype. J Immunol. (2009) 182:3902–18. 10.4049/jimmunol.0802124 19265170

[162] Jiang W Anderson MA Bronson R Mathis D Benoist C. Modifier loci condition autoimmunity provoked by Aire deficiency. J Exp Med. (2005) 202:805–15. 10.1084/jem.20050693 16172259 PMC2212943

[163] Niki S Oshikawa K Mouri Y Hirota F Matsushima A Yano M. Alteration of intra-pancreatic target-organ specificity by abrogation of Aire in NOD mice. J Clin Invest. (2006) 116:1292–301. 10.1172/JCI26971 16628255 PMC1440703

[164] Han H. Target-organ specificity of autoimmunity is modified by thymic stroma and bone marrow-derived cells. J Med Invest. (2007) 54:54–64. 10.2152/jmi.54.54 17380015

[165] Ossart J Moreau A Autrusseau E Ménoret S Martin JC Besnard M. Breakdown of immune tolerance in AIRE-deficient rats induces a severe autoimmune polyendocrinopathy-candidiasis-ectodermal dystrophy-like autoimmune disease. J Immunol. (2018) 201:874–87. 10.4049/jimmunol.1701318 29959280

[166] Derbinski J Gäbler J Brors B Tierling S Jonnakuty S Hergenhahn M. Promiscuous gene expression in thymic epithelial cells is regulated at multiple levels. J Exp Med. (2005) 202:33–45. 10.1084/jem.20050471 15983066 PMC2212909

[167] Shikama N Nusspaumer G Holländer GA. Clearing the AIRE: on the pathophysiological basis of the autoimmune polyendocrinopathy syndrome type-1. Endocrinol Metab Clin North Am. (2009) 38:273–88. 10.1016/j.ecl.2009.01.011 19328411

[168] Sansom SN Shikama-Dorn N Zhanybekova S Nusspaumer G Macaulay IC Deadman ME. Population and single-cell genomics reveal the *Aire* dependency, relief from Polycomb silencing, and distribution of self-antigen expression in thymic epithelia. Genome Res. (2014) 24:1918–31. 10.1101/gr.171645.113 25224068 PMC4248310

[169] Ströbel P Murumägi A Klein R Luster M Lahti M Krohn K. Deficiency of the autoimmune regulator AIRE in thymomas is insufficient to elicit autoimmune polyendocrinopathy syndrome type I (APS-I). J Pathol. (2007) 211:563–71. 10.1002/path.2141 17334980

[170] Org T Rebane A Kisand K Laan M Haljasorg U Andreson R. AIRE activated tissue specific genes have histone modifications associated with inactive chromatin. Hum Mol Genet. (2009) 18:4699–710. 10.1093/hmg/ddp433 19744957 PMC2778368

[171] Wolff AS Kärner J Owe JF Oftedal BE Gilhus NE Erichsen MM. Clinical and serologic parallels to APS-I in patients with thymomas and autoantigen transcripts in their tumors. J Immunol. (2014) 193:3880–90. 10.4049/jimmunol.1401068 25230752 PMC4190667

[172] Ostertag B. Die an bestimmte Lokalisation gebundenen Konkremente des Zentralnervensystems und ihre Beziehung zur “Verkalkung intracelebraler Getäße” beigewissen endokrinen Erkrankungen. Virchows Arch Pathol Anat Physiol Klin Med. (1930) 275:828–59. 10.1007/BF01947423

[173] Perheentupa J. Kaksi ryhmää lisämunuaiskuoren periytyviä vajaatoimintatiloja. Duodecim. (1972) 88:119–27. 5013565

[174] Perheentupa J Hiekkala H. Twenty cases of the syndrome of autoimmune endocrinopathy and candidiasis. Acta Paediatr Scand. (1973) 62:110–1. 10.1111/j.1651-2227.1973.tb08074.x

[175] Myllärniemi S Perheentupa J. Oral findings in the autoimmune polyendocrinopathy-candidosis syndrome (APECS) and other forms of hypoparathyroidism. Oral Surg Oral Med Oral Pathol. (1978) 45:721–9. 10.1016/0030-4220(78)90147-0 276791

[176] Perheentupa J Tiilikainen A Lokki ML. Autoimmune polyendocrinopathy-candidosis syndrome (APECS): clinical variation, inheritance and HLA association in 40 Finnish patients. Pediatr Res. (1978) 12:1087. 10.1203/00006450-197811000-00038

[177] Perheentupa J. Autoimmune Polyendocrinopathy – Candidosis – Ectodermal Dystrophy (APECED). In: Eriksson AW, Forsius HR, Nevanlinna HR, Workman PL, Norio RK, editors. Population Structure and Genetic Disorders. London: Academic Press. (1980). p. 583–7.

[178] Perheentupa J. Autoimmune polyendocrinopathy-candidiasis-ectodermal dystrophy (APECED). Horm Metab Res. (1996) 28:353–6. 10.1055/s-2007-979814 8858385

[179] Perheentupa J. APS-I/APECED: the clinical disease and therapy. Endocrinol Metab Clin North Am. (2002) 31:295–320. 10.1016/s0889-8529(01)00013-5 12092452

[180] Perheentupa J. Autoimmune polyendocrinopathy-candidiasis-ectodermal dystrophy. J Clin Endocrinol Metab. (2006) 91:2843–50. 10.1210/jc.2005-2611 16684821

[181] Wagman RD Kazdan JJ Kooh SW Fraser D. Keratitis associated with the multiple endocrine deficiency, autoimmune disease, and candidiasis syndrome. Am J Ophthalmol. (1987) 103:569–75. 10.1016/s0002-9394(14)74281-3 3565516

[182] Wang CY Davoodi-Semiromi A Huang W Connor E Shi JD She JX. Characterization of mutations in patients with autoimmune polyglandular syndrome type 1 (APS1). Hum Genet. (1998) 103:681–5. 10.1007/s004390050891 9921903

[183] Cihakova D Trebusak K Heino M Fadeyev V Tiulpakov A Battelino T. Novel AIRE mutations and P450 cytochrome autoantibodies in Central and Eastern European patients with APECED. Hum Mutat. (2001) 18:225–32. 10.1002/humu.1178 11524733

[184] Stolarski B Pronicka E Korniszewski L Pollak A Kostrzewa G Rowińska E. Molecular background of polyendocrinopathy-candidiasis-ectodermal dystrophy syndrome in a Polish population: novel *AIRE* mutations and an estimate of disease prevalence. Clin Genet. (2006) 70:348–54. 10.1111/j.1399-0004.2006.00690.x 16965330

[185] Dominguez M Crushell E Ilmarinen T McGovern E Collins S Chang B. Autoimmune polyendocrinopathy-candidiasis-ectodermal dystrophy (APECED) in the Irish population. J Pediatr Endocrinol Metab. (2006) 19:1343–52. 10.1515/jpem.2006.19.11.1343 17220063

[186] Adamson KA Cheetham TD Kendall-Taylor P Seckl JR Pearce SHS. The role of the *IDDM2* locus in the susceptibility of UK APS1 subjects to type 1 diabetes mellitus. Int J Immunogenet. (2007) 34:17–21. 10.1111/j.1744-313X.2006.00643.x 17284223

[187] Wolff ASB Erichsen MM Meager A Magitta NF Myhre AG Bollerslev J. Autoimmune polyendocrine syndrome type 1 in Norway: phenotypic variation, autoantibodies, and novel mutations in the autoimmune regulator gene. J Clin Endocrinol Metab. (2007) 92:595–603. 10.1210/jc.2006-1873 17118990

[188] Trebušak Podkrajšek K Milenković T Odink RJ Claasen-van der Grinten HL Bratanić N Hovnik T. Detection of a complete autoimmune regulator gene deletion and two additional novel mutations in a cohort of patients with atypical phenotypic variants of autoimmune polyglandular syndrome type 1. Eur J Endocrinol. 2008; 159:633–9. 10.1530/EJE-08-0328 18682433

[189] Zaidi G Sahu RP Zhang L George G Bhavani N Shah N. Two novel *Aire* mutations in autoimmune polyendocrinopathy-candidiasis-ectodermal dystrophy (APECED) among Indians. Clin Genet. (2009) 76:441–8. 10.1111/j.1399-0004.2009.01280.x 19807739

[190] Proust-Lemoine E Saugier-Véber P Lefranc D Dubucquoi S Ryndak A Buob D. Autoimmune polyendocrine syndrome type 1 in North-Western France: *AIRE* gene mutation specificities and severe forms needing immunosuppressive therapies. Horm Res Paediatr. (2010) 74:275–84. 10.1159/000297714 20453472

[191] Tóth B Wolff ASB Halász Z Tar A Szüts P Ilyés I. Novel sequence variation of *AIRE* and detection of interferon-ω antibodies in early infancy. Clin Endocrinol (Oxf). (2010) 72:641–7. 10.1111/j.1365-2265.2009.03740.x 19863576

[192] Orlova EM Bukina AM Kuznetsova ES Kareva MA Zakharova EU Peterkova VA. Autoimmune polyglandular syndrome type 1 in Russian patients: clinical variants and autoimmune regulator mutations. Horm Res Paediatr. (2010) 73:449–57. 10.1159/000313585 20407228

[193] Bin-Abbas BS Faiyaz-Ul-Haque M Al-Fares AH Al-Gazlan SS Bhuiyan JA Al-Muhsen SZ. Autoimmune polyglandular syndrome type 1 in Saudi children. Saudi Med J. (2010) 31:788–92. 20635013

[194] Meloni A Willcox N Meager A Atzeni M Wolff ASB Husebye ES. Autoimmune polyendocrine syndrome type 1: an extensive longitudinal study in Sardinian patients. J Clin Endocrinol Metab. (2012) 97:1114–24. 10.1210/jc.2011-2461 22344197

[195] Bratanic N Kisand K Avbelj Stefanija M Battelino T Trebusak Podkrajsek K. Clinical, genetic and immunological characteristics of paediatric autoimmune polyglandular syndrome type 1 patients in Slovenia. Zdrav Var. (2015) 54:112–8. 10.1515/sjph-2015-0017 27646917 PMC4820163

[196] Fierabracci A Pellegrino M Frasca F Kilic SS Betterle C. APECED in Turkey: a case report and insights on genetic and phenotypic variability. Clin Immunol. (2018) 194:60–6. 10.1016/j.clim.2018.06.012 30018023

[197] Weiler FG Peterson P Costa-Carvalho BT de Barros Dorna M Correia-Deur JE Sader SL. The heterogeneity of autoimmune polyendocrine syndrome type 1: clinical features, new mutations and cytokine autoantibodies in a Brazilian cohort from tertiary care centers. Clin Immunol. (2018) 197:231–8. 10.1016/j.clim.2018.09.012 30287219

[198] de Moraes Ruehsen M Blizzard RM Garcia-Bunuel R Jones GS. Autoimmunity and ovarian failure. Am J Obstet Gynecol. (1972) 112:693–703. 10.1016/0002-9378(72)90797-1 4551032

[199] Fénichel P Sosset C Barbarino-Monnier P Gobert B Hiéronimus S Béné MC. Prevalence, specificity and significance of ovarian antibodies during spontaneous premature ovarian failure. Hum Reprod. (1997) 12:2623–8. 10.1093/humrep/12.12.2623 9455825

[200] Moncayo R Moncayo HE. The association of autoantibodies directed against ovarian antigens in human disease: a clinical review. J Intern Med. (1993) 234:371–8. 10.1111/j.1365-2796.1993.tb00758.x 8409833

[201] Hoek A Schoemaker J Drexhage HA. Premature ovarian failure and ovarian autoimmunity. Endocr Rev. (1997) 18:107–34. 10.1210/edrv.18.1.0291 9034788

[202] Kalantaridou SN Davs SR Nelson LM. Premature ovarian failure. Endocrinol Metab Clin North Am. (1998) 27:989–1006. 10.1016/s0889-8529(05)70051-7 9922918

[203] Anasti JN. Premature ovarian failure: an update. Fertil Steril. (1998) 70:1–15. 10.1016/s0015-0282(98)00099-5 9660412

[204] Christin-Maitre S Vasseur C Portnoï MF Bouchard P. Genes and premature ovarian failure. Mol Cell Endocrinol. (1998) 145:75–80. 10.1016/s0303-7207(98)00172-5 9922102

[205] Maclaren N Chen QY Kukreja A Marker J Zhang CH Sun ZS. Autoimmune hypogonadism as part of an autoimmune polyglandular syndrome. J Soc Gynecol Investig. (2001) 8:S52–4. 10.1016/s1071-5576(00)00109-x 11223374

[206] Laml T Preyer O Umek W Hengstschläger M Hanzal E. Genetic disorders in premature ovarian failure. Hum Reprod Update. (2002) 8:483–91. 10.1093/humupd/8.5.483 12398227

[207] Layman LC. Human gene mutations causing infertility. J Med Genet. (2002) 39:153–61. 10.1136/jmg.39.3.153 11897813 PMC1735054

[208] Practice Committee of the American Society for Reproductive Medicine. Current evaluation of amenorrhea. Fertil Steril. (2004) 82:266–72. 10.1016/j.fertnstert.2004.02.098 15237040

[209] Forges T Monnier-Barbarino P Faure GC Béné MC. Autoimmunity and antigenic targets in ovarian pathology. Hum Reprod Update. (2004) 10:163–75. 10.1093/humupd/dmh014 15073145

[210] Goswami D Conway GS. Premature ovarian failure. Hum Reprod Update. (2005) 11:391–410. 10.1093/humupd/dmi012 15919682

[211] Welt CK. Autoimmune oophoritis in the adolescent. Ann N Y Acad Sci. (2008) 1135:118–22. 10.1196/annals.1429.006 18574216

[212] Broekmans FJ Soules MR Fauser BC. Ovarian aging: mechanisms and clinical consequences. Endocr Rev. (2009) 30:465–93. 10.1210/er.2009-0006 19589949

[213] Nelson LM. Primary ovarian insufficiency. N Engl J Med. (2009) 360:606–14. 10.1056/NEJMcp0808697 19196677 PMC2762081

[214] Kokcu A. Premature ovarian failure from current perspective. Gynecol Endocrinol. (2010) 26:555–62. 10.3109/09513590.2010.488773 20500113

[215] Persani L Rossetti R Cacciatore C. Genes involved in human premature ovarian failure. J Mol Endocrinol. (2010) 45:257–79. 10.1677/JME-10-0070 20668067

[216] Warren BD Kinsey WK McGinnis LK Christenson LK Jasti S Stevens AM. Ovarian autoimmune disease: clinical concepts and animal models. Cell Mol Immunol. (2014) 11:510–21. 10.1038/cmi.2014.97 25327908 PMC4220844

[217] Greene AD Patounakis G Segars JH. Genetic associations with diminished ovarian reserve: a systematic review of the literature. J Assist Reprod Genet. (2014) 31:935–46. 10.1007/s10815-014-0257-5 24840722 PMC4130940

[218] Kirshenbaum M Orvieto R. Premature ovarian insufficiency (POI) and autoimmunity–an update appraisal. J Assist Reprod Genet. (2019) 36:2207–15. 10.1007/s10815-019-01572-0 31440958 PMC6885484

[219] Saari V Holopainen E Mäkitie O Laakso S. Pubertal development and premature ovarian insufficiency in patients with APECED. Eur J Endocrinol. (2020) 183:513–20. 10.1530/EJE-20-0516 33107435

[220] Irvine WJ Barnes EW. Addison's disease, ovarian failure and hypoparathyroidism. Clin Endocrinol Metab. (1975) 4:379–434. 10.1016/S0300-595X(75)80027-2

[221] Muir A Maclaren NK. Autoimmune diseases of the adrenal glands, parathyroid glands, gonads, and hypothalamic-pituitary axis. Endocrinol Metab Clin North Am. (1991) 20:619–44. 10.1016/S0889-8529(18)30261-5 1935921

[222] Kasperlik-Zaluska AA Migdalska B Czarnocka B Drac-Kaniewska J Niegowska E Czech W. Association of Addison's disease with autoimmune disorders – a long term observation of 180 patients. Postgrad Med J. (1991) 67:984–7. 10.1136/pgmj.67.793.984 1775423 PMC2399126

[223] Kong MF Jeffcoate W. Eighty-six cases of Addison's disease. Clin Endocrinol (Oxf). (1994) 41:757–61. 10.1111/j.1365-2265.1994.tb02790.x 7889611

[224] Betterle C Dal Pra C Greggio N Volpato M Zanchetta R. Autoimmunity in isolated Addison's disease and in polyglandular autoimmune diseases type 1, 2 and 4. Ann Endocrinol (Paris). (2001) 62:193–201. 10.1210/AE-04-2001-62-2-0003-4266-101019-ART2 11353894

[225] Falorni A Laureti S De Bellis A Zanchetta R Tiberti C Arnaldi G. Italian Addison Network Study: update of diagnostic criteria for the etiological classification of primary adrenal insufficiency. J Clin Endocrinol Metab. (2004) 89:1598–604. 10.1210/jc.2003-030954 15070918

[226] Perry R Kecha O Paquette J Huot C Van Vliet G Deal C. Primary adrenal insufficiency in children: twenty years experience at the Sainte-Justine Hospital, Montreal. J Clin Endocrinol Metab. (2005) 90:3243–50. 10.1210/jc.2004-0016 15811934

[227] Kasperlik-Zaluska AA Czarnocka B Jeske W Papierska L. Addison's disease revisited in Poland: year 2008 versus year 1990. Autoimmune Dis. (2010) 2010:e731834. 10.4061/2010/731834 21188237 PMC3005832

[228] Dalin F Nordling Eriksson G Dahlqvist P Hallgren Å Wahlberg J Ekwall O. Clinical and immunological characteristics of autoimmune Addison Disease: a nationwide Swedish multicenter study. J Clin Endocrinol Metab. (2017) 102:379–89. 10.1210/jc.2016-2522 27870550

[229] Regan EA Vaidya A Margulies PL Make BJ Lowe KE Crapo JD. Primary adrenal insufficiency in the United States: diagnostic error and patient satisfaction with treatment. Diagnosis (Berl). (2019) 6:343–50. 10.1515/dx-2019-0013 31256064

[230] Saenger P Levine LS Irvine WJ Gottesdiener K Rauh W Sonino N. Progressive adrenal failure in polyglandular autoimmune disease. J Clin Endocrinol Metab. (1982) 54:863–8. 10.1210/jcem-54-4-863 6277984

[231] Leisti S Ahonen P Perheentupa J. The diagnosis and staging of hypocortisolism in progressing autoimmune adrenalitis. Pediatr Res. (1983) 17:861–7. 10.1203/00006450-198311000-00005 6316243

[232] Ketchum CH Riley WJ Maclaren NK. Adrenal dysfunction in asymptomatic patients with adrenocortical autoantibodies. J Clin Endocrinol Metab. (1984) 58:1166–70. 10.1210/jcem-58-6-1166 6725513

[233] Ahonen P Miettinen A Perheentupa J. Adrenal and steroidal cell antibodies in patients with autoimmune polyglandular disease type I and risk of adrenocortical and ovarian failure. J Clin Endocrinol Metab. (1987) 64:494–500. 10.1210/jcem-64-3-494 3818889

[234] Betterle C Scalici C Presotto F Pedini B Moro L Rigon F. The natural history of adrenal function in autoimmune patients with adrenal autoantibodies. J Endocrinol. (1988) 117:467–75. 10.1677/joe.0.1170467 3392502

[235] Barat P Joussein M Carel JC. À propos de deux cas de déficit en minéralo-corticoïdes dû à une première manifestation endocrinienne du syndrome APECED. Arch Pediatr. (2003) 10:137–9. 10.1016/s0929-693x(03)00311-7 12829356

[236] Løvås K Husebye ES. Replacement therapy in Addison's disease. Expert Opin Pharmacother. (2003) 4:2145–9. 10.1517/14656566.4.12.2145 14640913

[237] Jeffcoate WJ Hosking DJ Jones RM. Hypoparathyroidism and Addison's disease: a potentially lethal combination. J R Soc Med. (1987) 80:709–10. 10.1177/014107688708001119 3694620 PMC1291098

[238] Husebye ES Perheentupa J Rautemaa R Kämpe O. Clinical manifestations and management of patients with autoimmune polyendocrine syndrome type I. J Intern Med. (2009) 265:514–29. 10.1111/j.1365-2796.2009.02090.x 19382991

[239] Mäkitie O Sochett EB Bondestam S Sipilä I Perheentupa J. Bone health in autoimmune polyendocrinopathy-candidiasis-ectodermal dystrophy (APECED): findings in 25 adults. Clin Endocrinol (Oxf). (2006) 64:489–94. 10.1111/j.1365-2265.2006.02495.x 16649965

[240] Gharib H Gastineau CF Hodgson SF Scholz DA Smith LA. Reversible hypothyroidism in Addison's disease. Lancet. (1972) 300:734–6. 10.1016/s0140-6736(72)92024-7 4116146

[241] Topliss DJ White EL Stockigt JR. Significance of thyrotropin excess in untreated primary adrenal insufficiency. J Clin Endocrinol Metab. (1980) 50:52–6. 10.1210/jcem-50-1-52 6765953

[242] Ismail AAA Burr WA Walker PL. Acute changes in serum thyrotrophin in treated Addison's disease. Clin Endocrinol (Oxf). (1989) 30:225–30. 10.1111/j.1365-2265.1989.tb02230.x 2591055

[243] Løvås K Loge JH Husebye ES. Subjective health status in Norwegian patients with Addison's disease. Clin Endocrinol (Oxf). (2002) 56:581–8. 10.1046/j.1365-2265.2002.01466.x 12030907

[244] Løvas K Husebye ES Holsten F Bjorvatn B. Sleep disturbances in patients with Addison's disease. Eur J Endocrinol. (2003) 148:449–56. 10.1530/eje.0.1480449 12656666

[245] Løvås K Gjesdal CG Christensen M Wolff AB Almås B Svartberg J. Glucocorticoid replacement therapy and pharmacogenetics in Addison's disease: effects on bone. Eur J Endocrinol. (2009) 160:993–1002. 10.1530/EJE-08-0880 19282465

[246] Husebye ES Allolio B Arlt W Badenhoop K Bensing S Betterle C. Consensus statement on the diagnosis, treatment and follow-up of patients with primary adrenal insufficiency. J Intern Med. (2014) 275:104–15. 10.1111/joim.12162 24330030

[247] Anderson JR Goudie RB Gray KG Timbury GC. Auto-antibodies in Addison's disease. Lancet. (1957) 269:1123–4. 10.1016/s0140-6736(57)91687-2 13439984

[248] Blizzard RM Kyle MA Chandler RW Hung W. Adrenal antibodies in Addison's disease. Lancet. (1962) 280:901–3. 10.1016/s0140-6736(62)90681-5 13971664

[249] Blizzard RM Kyle M. Studies of the adrenal antigens and antibodies in Addison's disease. J Clin Invest. (1963) 42:1653–60. 10.1172/JCI104851 14074360 PMC289445

[250] Goudie RB Anderson JR Gray KK Whyte WG. Autoantibodies in Addison's disease. Lancet. (1966) 287:1173–6. 10.1016/S0140-6736(66)91070-1

[251] Blizzard RM Chee D Davis W. The incidence of adrenal and other antibodies in the sera of patients with idiopathic adrenal insufficiency (Addison's disease). Clin Exp Immunol. (1967) 2:19–30. 6030795 PMC1578816

[252] Irvine WJ Stewart AG Scarth L. A clinical and immunological study of adrenocortical insufficiency (Addison's disease). Clin Exp Immunol. (1967) 2:31–69. 5340030 PMC1578820

[253] Goudie RB McDonald E Anderson JR Gray K. Immunological features of idiopathic Addison's disease: characterization of the adrenocortical antigens. Clin Exp Immunol. (1968) 3:119–31. 4171048 PMC1578877

[254] Irvine WJ. Clinical and immunological associations in adrenal disorders. Proc R Soc Med. (1968) 61:271–5. 4870467 10.1177/003591576806100329 PMC1902263

[255] Irvine WJ Scarth L. Antibody to the oxyphil cells of the human parathyroid in idiopathic hypoparathyroidism. Clin Exp Immunol. (1969) 4:505–10. 4892044 PMC1579075

[256] Irvine WJ Chan MMW Scarth L Kolb FO Hartog M Bayliss RIS. Immunological aspects of premature ovarian failure associated with idiopathic Addison's disease. Lancet. (1968) 292:883–7. 10.1016/s0140-6736(68)91053-2 4176147

[257] Irvine WJ Chan MMW Scarth L. The further characterization of autoantibodies reactive with extra-adrenal steroid-producing cells in patients with adrenal disorders. Clin Exp Immunol. (1969) 4:489–503. 4892043 PMC1579074

[258] Anderson JR Goudie RB Gray K Stuart-Smith DA. Immunological features of idiopathic Addison's disease: an antibody to cells producing steroid hormones. Clin Exp Immunol. (1968) 3:107–17. 4868174 PMC1578871

[259] McNatty KP Short RV Barnes EW Irvine WJ. The cytotoxic effect of serum from patients with Addison's disease and autoimmune ovarian failure of human granulosa cells in culture. Clin Exp Immunol. (1975) 22:378–84. 1225483 PMC1538450

[260] Sotsiou F Bottazzo GF Doniach D. Immunofluorescence studies on autoantibodies to steroid-producing cells, and to germline cells in endocrine disease and infertility. Clin Exp Immunol. (1980) 39:97–111. 6771073 PMC1537958

[261] Khoury EL Hammond L Bottazzo GF Doniach D. Surface-reactive antibodies to human adrenal cells in Addison's disease. Clin Exp Immunol. (1981) 45:48–55. 7030540 PMC1537257

[262] Elder M Maclaren N Riley W. Gonadal autoantibodies in patients with hypogonadism and/or Addison's disease. J Clin Endocrinol Metab. (1981) 52:1137–42. 10.1210/jcem-52-6-1137 7014594

[263] Irvine WJ. Premature menopause in autoimmune diseases. Lancet. (1969) 293:264. 10.1016/s0140-6736(69)91280-x 4178628

[264] Vallotton MB Forbes AP. Antibodies to cytoplasm of ova. Lancet. (1966) 288:264–5. 10.1016/s0140-6736(66)92546-3 4161424

[265] Vallotton MB Forbes AP. Autoimmunity in gonadal dysgenesis and Klinefelter syndrome. Lancet. (1967) 289:648–51. 10.1016/s0140-6736(67)92543-3 4163925

[266] Vallotton MB Forbes AP. Premature menopause in autoimmune diseases. Lancet. (1969) 293:156–7. 10.1016/s0140-6736(69)91171-4 4178272

[267] Krohn K Heinonen E Pelkonen R Perheentupa J. Precipitating antiadrenal antibodies in Addison's disease. Scand J Immunol. (1973) 2:450. 10.1016/0090-1229(74)90023-3

[268] Krohn K Perheentupa J Heinonen E. Precipitating anti-adrenal antibodies in Addison's disease. Clin Immunol Immunopathol. (1974) 3:59–68. 4215605 10.1016/0090-1229(74)90023-3

[269] Heinonen E Krohn K Perheentupa J Aro A Pelkonen R. Association of precipitating anti-adrenal antibodies with moniliasis-polyendocrinopathy syndrome. Ann Clin Res. (1976) 8:262–5. 999212

[270] Heinonen E. Variety of determinants in an adrenal antigen common to man and some animals. Med Biol. (1976) 54:341–6. 62895

[271] Heinonen E Krohn K. Studies on an adrenal antigen common to man and different animals. Med Biol. (1977) 55:48–53. 66422

[272] Matteson KJ Picado-Leonard J Chung BC Mohandas TK Miller WL. Assignment of the gene for adrenal P450c17 (steroid 17α-hydroxylase/17,20 lyase) to human chromosome 10. J Clin Endocrinol Metab. (1986) 63:789–91. 10.1210/jcem-63-3-789 3488328

[273] White PC New MI Dupont B. Structure of human steroid 21-hydroxylase genes. Proc Natl Acad Sci USA. (1986) 83:5111–5. 10.1073/pnas.83.14.5111 3487786 PMC323900

[274] Chung B Matteson KJ Voutilainen R Mohandas TK Miller WL. Human cholesterol side-chain cleavage enzyme, P450scc: cDNA cloning, assignment of the gene to chromosome 15, and expression in the placenta. Proc Natl Acad Sci USA. (1986) 83:8962–6. 10.1073/pnas.83.23.8962 3024157 PMC387054

[275] Winqvist O Karlsson FA Kämpe O. 21-hydroxylase, a major autoantigen in idiopathic Addison's disease. Lancet. (1992) 339:1559–62. 10.1016/0140-6736(92)91829-w 1351548

[276] Bednarek J Furmaniak J Wedlock N Kiso Y Baumann-Antczak A Fowler S. Steroid 21-hydroxylase is a major autoantigen involved in adult onset autoimmune Addison's disease. FEBS Lett. (1992) 309:51–5. 10.1016/0014-5793(92)80737-2 1511745

[277] Baumann-Antczak A Wedlock N Bednarek J Kiso Y Krishnan H Fowler S. Autoimmune Addison's disease and 21-hydroxylase. Lancet. (1992) 340:429–30. 10.1016/0140-6736(92)91513-8 1353582

[278] Krohn K Uibo R Aavik E Peterson P Savilahti K. Identification by molecular cloning of an autoantigen associated with Addison's disease as steroid 17α-hydroxylase. Lancet. (1992) 339:770–3. 10.1016/0140-6736(92)91894-e 1347802

[279] Winqvist O Gustafsson J Rorsman F Karlsson FA Kämpe O. Two different cytochrome P450 enzymes are the adrenal antigens in autoimmune polyendocrine syndrome type I and Addison's disease. J Clin Invest. (1993) 92:2377–85. 10.1172/JCI116843 8227354 PMC288420

[280] Karlsson FA Kämpe O Winqvist O Burman P. Autoimmune disease of the adrenal cortex, pituitary, parathyroid glands and gastric mucosa. J Intern Med. (1993) 234:379–86. 10.1111/j.1365-2796.1993.tb00759.x 8409834

[281] Uibo R Perheentupa J Ovod V Krohn KJE. Characterization of adrenal autoantigens recognized by sera from patients with autoimmune polyglandular syndrome (APS) type I. J Autoimmun. (1994) 7:399–411. 10.1006/jaut.1994.1029 7916911

[282] Weetman AP. Autoimmunity to steroid-producing cells and familial polyendocrine autoimmunity. Baillieres Clin Endocrinol Metab. (1995) 9:157–74. 10.1016/s0950-351x(95)80899-x 7726795

[283] Uibo R Aavik E Peterson P Perheentupa J Aranko S Pelkonen R. Autoantibodies to cytochrome P450 enzymes P450scc, P450c17, and P450c21 in autoimmune polyglandular disease types I and II and in isolated Addison's disease. J Clin Endocrinol Metab. (1994) 78:323–8. 10.1210/jcem.78.2.8106620 8106620

[284] Colls J Betterle C Volpato M Prentice L Rees Smith B Furmaniak J. Immunoprecipitation assay for autoantibodies to steroid 21-hydroxylase in autoimmune adrenal diseases. Clin Chem. (1995) 41:375–80. 7882511

[285] Falorni A Nikoshkov A Laureti S Grenbäck E Hulting AL Casucci G. High diagnostic accuracy for idiopathic Addison's disease with a sensitive radiobinding assay for autoantibodies against recombinant human 21-hydroxylase. J Clin Endocrinol Metab. (1995) 80:2752–5. 10.1210/jcem.80.9.7673419 7673419

[286] Söderbergh A Winqvist O Norheim I Rorsman F Husebye ES Dolva Ø. Adrenal autoantibodies and organ-specific autoimmunity in patients with Addison's disease. Clin Endocrinol (Oxf). (1996) 45:453–60. 10.1046/j.1365-2265.1996.8040813.x 8959085

[287] Chen S Sawicka J Betterle C Powell M Prentice L Volpato M. Autoantibodies to steroidogenic enzymes in autoimmune polyglandular syndrome, Addison's disease, and premature ovarian failure. J Clin Endocrinol Metab. (1996) 81:1871–6. 10.1210/jcem.81.5.8626850 8626850

[288] Falorni A Laureti S Nikoshkov A Picchio ML Hallengren B Vandewalle CL. 21-hydroxylase autoantibodies in adult patients with endocrine autoimmune diseases are highly specific for Addison's disease. Clin Exp Immunol. (1997) 107:341–6. 10.1111/j.1365-2249.1997.262-ce1153.x 9030873 PMC1904588

[289] Tanaka H Perez MS Powell M Sanders JF Sawicka J Chen S. Steroid 21-hydroxylase autoantibodies: measurements with a new immunoprecipitation assay. J Clin Endocrinol Metab. (1997) 82:1440–6. 10.1210/jcem.82.5.3929 9141530

[290] Betterle C Volpato M Pedini B Chen S Furmaniak J Rees Smith B. Adrenal-cortex and 21-hydroxylase autoantibodies in autoimmune Addison's disease. J Endocrinol Invest. (1997) 20(S4):91.9062510

[291] Degros V Pons L Ghulam A Racadot A. Intérêt du dosage des anticorps anti-21 hydroxylase comme marqueur de l'atteinte surrénale dans les endocrinopathies auto-immunes. Ann Biol Clin. (1999) 57:705–9. 10572219

[292] Seissler J Schott M Steinbrenner H Peterson P Scherbaum WA. Autoantibodies to adrenal cytochrome P450 antigens in isolated Addison's disease and autoimmune polyendocrine syndrome type II. Exp Clin Endocrinol Diabetes. (1999) 107:208–13. 10.1055/s-0029-1212100 10376448

[293] Betterle C Volpato M Pedini B Chen S Rees Smith B Furmaniak J. Adrenal-cortex autoantibodies and steroid-producing cells autoantibodies in patients with Addison's disease: comparison of immunofluorescence and immunoprecipitation assays. J Clin Endocrinol Metab. (1999) 84:618–22. 10.1210/jcem.84.2.5459 10022426

[294] Reato G Morlin L Chen S Furmaniak J Rees Smith B Masiero S. Premature ovarian failure in patients with autoimmune Addison's disease: clinical, genetic, and immunological evaluation. J Clin Endocrinol Metab. (2011) 96:E1255–61. 10.1210/jc.2011-0414 21677034

[295] Dalla Costa M Bonanni G Masiero S Faggian D Chen S Furmaniak J. Gonadal function in males with autoimmune Addison's disease and autoantibodies to steroidogenic enzymes. Clin Exp Immunol. (2014) 176:373–9. 10.1111/cei.12303 24666377 PMC4008981

[296] Perniola R Falorni A Clemente MG Forini F Accogli E Lobreglio G. Organ-specific and non-organ-specific autoantibodies in children and young adults with autoimmune polyendocrinopathy-candidiasis-ectodermal dystrophy (APECED). Eur J Endocrinol. (2000) 143:497–503. 10.1530/eje.0.1430497 11022196

[297] Myhre AG Halonen M Eskelin P Ekwall O Hedstrand H Rorsman F. Autoimmune polyendocrine syndrome type 1 (APS I) in Norway. Clin Endocrinol (Oxf). (2001) 54:211–7. 10.1046/j.1365-2265.2001.01201.x 11207636

[298] Söderbergh A Myhre AG Ekwall O Gebre-Medhin G Hedstrand H Landgren E. Prevalence and clinical associations of 10 defined autoantibodies in autoimmune polyendocrine syndrome type I. J Clin Endocrinol Metab. (2004) 89:557–62. 10.1210/jc.2003-030279 14764761

[299] Betterle C Rossi A Dalla Pria S Artifoni A Pedini B Gavasso S. Premature ovarian failure: autoimmunity and natural history. Clin Endocrinol (Oxf). (1993) 39:35–43. 10.1111/j.1365-2265.1993.tb01748.x 8348706

[300] Winqvist O Gebre-Medhin G Gustafsson J Ritzén EM Lundkvist Ö Karlsson FA. Identification of the main gonadal autoantigens in patients with adrenal insufficiency and associated ovarian failure. J Clin Endocrinol Metab. (1995) 80:1717–23. 10.1210/jcem.80.5.7745025 7745025

[301] Falorni A Laureti S Candeloro P Perrino S Coronella C Bizzarro A. Steroid-cell autoantibodies are preferentially expressed in women with premature ovarian failure who have adrenal autoimmunity. Fertil Steril. (2002) 78:270–9. 10.1016/s0015-0282(02)03205-3 12137862

[302] Dal Pra C Chen S Furmaniak J Rees Smith B Pedini B Moscon A. Autoantibodies to steroidogenic enzymes in patients with premature ovarian failure with and without Addison's disease. Eur J Endocrinol. (2003) 148:565–70. 10.1530/eje.0.1480565 12720541

[303] Reimand K Peterson P Hyöti H Uibo R Cooke I Weetman AP. 3β-Hydroxysteroid dehydrogenase autoantibodies are rare in premature ovarian failure. J Clin Endocrinol Metab. (2000) 85:2324–6. 10.1210/jcem.85.6.6630 10852471

[304] Betterle C Zanette F Zanchetta R Pedini B Trevisan A Mantero F. Complement-fixing adrenal autoantibodies as a marker for predicting onset of idiopathic Addison's disease. Lancet. (1983) 321:1238–41. 10.1016/s0140-6736(83)92695-8 6134038

[305] Betterle C Volpato M Rees Smith B Furmaniak J Chen S Greggio NA. I. Adrenal cortex and steroid 21-hydroxylase autoantibodies in adult patients with organ-specific autoimmune diseases: markers of low progression to clinical Addison's disease. J Clin Endocrinol Metab. (1997) 82:932–8. 10.1210/jcem.82.3.3819 9062509

[306] Betterle C Volpato M Rees Smith B Furmaniak J Chen S Zanchetta R. II. Adrenal cortex and steroid 21-hydroxylase autoantibodies in children with organ-specific autoimmune diseases: markers of high progression to clinical Addison's disease. J Clin Endocrinol Metab. (1997) 82:939–42. 10.1210/jcem.82.3.3849 9062510

[307] Betterle C Coco G Zanchetta R. Adrenal cortex autoantibodies in subjects with normal adrenal function. Best Pract Res Clin Endocrinol Metab. (2005) 19:85–99. 10.1016/j.beem.2004.11.008 15826924

[308] Coco G Dal Pra C Presotto F Albergoni MP Canova C Pedini B. Estimated risk for developing autoimmune Addison's disease in patients with adrenal cortex autoantibodies. J Clin Endocrinol Metab. (2006) 91:1637–45. 10.1210/jc.2005-0860 16522688

[309] Wedlock N Asawa T Baumann-Antczak A Rees Smith B Furmaniak J. Autoimmune Addison's disease. Analysis of autoantibody binding sites on human steroid 21-hydroxylase. FEBS Lett. (1993) 332:123–6. 10.1016/0014-5793(93)80497-i 8405426

[310] Song YH Connor EL Muir A She JX Zorovich B Derovanesian D. Autoantibody epitope mapping of the 21-hydroxylase antigen in autoimmune Addison's disease. J Clin Endocrinol Metab. (1994) 78:1108–12. 10.1210/jcem.78.5.7513715 7513715

[311] Volpato M Prentice L Chen S Betterle C Rees Smith B Furmaniak J. A study of the epitopes on steroid 21-hydroxylase recognized by autoantibodies in patients with or without Addison's disease. Clin Exp Immunol. (1998) 111:422–8. 10.1046/j.1365-2249.1998.00475.x 9486414 PMC1904918

[312] Chen S Sawicka J Prentice L Sanders JF Tanaka H Petersen V. Analysis of autoantibody epitopes on steroid 21-hydroxylase using a panel of monoclonal antibodies. J Clin Endocrinol Metab. (1998) 83:2977–86. 10.1210/jcem.83.8.5010 9709979

[313] Liiv I Teesalu K Peterson P Clemente MG Perheentupa J Uibo R. Epitope mapping of cytochrome P450 cholesterol side-chain cleavage enzyme by sera from patients with autoimmune polyglandular syndrome type 1. Eur J Endocrinol. (2002) 146:113–9. 10.1530/eje.0.1460113 11751076

[314] Peterson P Krohn KJE Mapping of B cell epitopes on steroid 17α-hydroxylase an autoantigen in autoimmune polyglandular syndrome type I. Clin Exp Immunol. (1994) 98:104–9. 10.1111/j.1365-2249.1994.tb06614.x 7523005 PMC1534182

[315] Weetman AP. Autoantigens in Addison's disease and associated syndromes. Clin Exp Immunol. (1997) 107:227–9. 10.1111/j.1365-2249.1997.1137a.x 9030856 PMC1904566

[316] Peterson P Uibo R Peränen J Krohn K. Immunoprecipitation of steroidogenic enzyme autoantigens with autoimmune polyglandular syndrome type I (APS I) sera; further evidence for independent humoral immunity to P450c17 and P450c21. Clin Exp Immunol. (1997) 107:335–40. 10.1111/j.1365-2249.1997.282-ce1175.x 9030872 PMC1904569

[317] Bøe AS Bredholt G Knappskog PM Hjelmervik TO Mellgren G Winqvist O. Autoantibodies against 21-hydroxylase and side-chain cleavage enzyme in autoimmune Addison's disease are mainly immunoglobulin G1. Eur J Endocrinol. (2004) 150:49–56. 10.1530/eje.0.1500049 14713279

[318] Brozzetti A Marzotti S La Torre D Bacosi ML Morelli S Bini V. Autoantibody responses in autoimmune ovarian insufficiency and in Addison's disease are IgG1 dominated and suggest a predominant, but not exclusive, Th1 type of response. Eur J Endocrinol. (2010) 163:309–17. 10.1530/EJE-10-0257 20498138

[319] Kendall-Taylor P Lambert A Mitchell R Robertson WR. Antibody that blocks stimulation of cortisol secretion by adrenocorticotrophic hormone in Addison's disease. Br Med J (Clin Res Ed). (1988) 296:1489–91. 10.1136/bmj.296.6635.1489 2839266 PMC2546014

[320] Reimand K Perheentupa J Link M Krohn K Peterson P Uibo R. Testis-expressed protein TSGA10 – an auto-antigen in autoimmune polyendocrine syndrome type I. Int Immunol. (2008) 20:39–44. 10.1093/intimm/dxm118 18000009

[321] Smith CJA Oscarson M Rönnblom L Alimohammadi M Perheentupa J Husebye ES. TSGA10 – a target for autoantibodies in autoimmune polyendocrine syndrome type 1 and systemic lupus erythematosus. Scand J Immunol. (2011) 73:147–53. 10.1111/j.1365-3083.2010.02486.x 21198756

[322] Tong ZB Nelson LM. A mouse gene encoding an oocyte antigen associated with premature ovarian failure. Endocrinology. (1999) 140:3720–6. 10.1210/endo.140.8.6911 10433232

[323] Otsuka N Tong ZB Vanevski K Tu W Cheng MH Nelson LM. Autoimmune oophoritis with multiple molecular targets mitigated by transgenic expression of mater. Endocrinology. (2011) 152:2465–73. 10.1210/en.2011-0022 21447630 PMC3100611

[324] Brozzetti A Alimohammadi M Morelli S Minarelli V Hallgren Å Giordano R. Autoantibody response against NALP5/MATER in primary ovarian insufficiency and in autoimmune Addison's disease. J Clin Endocrinol Metab. (2015) 100:1941–8. 10.1210/jc.2014-3571 25734249

[325] Moritz CP Paul S Stoevesandt O Tholance Y Camdessanché JP Antoine JC. Autoantigenomics: holistic characterization of autoantigen repertoires for a better understanding of autoimmune diseases. Autoimmun Rev. (2020) 19:e102450. 10.1016/j.autrev.2019.102450 31838165

[326] Landegren N Sharon D Freyhult E Hallgren Å Eriksson D Edqvist PH. Proteome-wide survey of the autoimmune target repertoire in autoimmune polyendocrine syndrome type 1. Sci Rep. (2016) 6:e20104. 10.1038/srep20104 26830021 PMC4735587

[327] Vazquez SE Ferré EMN Scheel DW Sunshine S Miao B Mandel-Brehm C. Identification of novel, clinically correlated autoantigens in the monogenic autoimmune syndrome APS1 by proteome-wide PhIP-Seq. eLife. (2020) 9:e55053. 10.7554/eLife.55053 32410729 PMC7228772

[328] Winqvist O Söderbergh A Kämpe O. The autoimmune basis of adrenocortical destruction in Addison's disease. Mol Med Today. (1996) 2:282–9. 10.1016/1357-4310(96)10024-1 8796908

[329] Rabinowe SL Jackson RA Dluhy RG Williams GH. Ia-positive T lymphocytes in recently diagnosed idiopathic Addison's disease. Am J Med. (1984) 77:597–601. 10.1016/0002-9343(84)90348-6 6333179

[330] Šedivá A Ciháková D Lebl J. Immunological findings in patients with autoimmune polyendocrinopathy-candidiasis-ectodermal dystrophy (APECED) and their family members: are heterozygotes subclinically affected? J Pediatr Endocrinol Metab. (2002) 15:1491–6. 10.1515/jpem.2002.15.9.1491 12503856

[331] Perniola R Lobreglio G Rosatelli MC Pitotti E Accogli E De Rinaldis C. Immunophenotypic characterisation of peripheral blood lymphocytes in autoimmune polyglandular syndrome type 1: clinical study and review of the literature. J Pediatr Endocrinol Metab. (2005) 18:155–64. 10.1515/jpem.2005.18.2.155 15751604

[332] Przybylski P Kurowska M Glazer M Plewik D Radej S Wiktor K. Analysis of blood dendritic cells and lymphocytes in patients with autoimmune polyglandular syndromes (APS) and isolated autoimmune endocrine diseases – a pilot study. Ann Univ Mariae Curie Sklodowska Med. (2008) 63:83–7. 10.2478/v10079-008-0055-6

[333] Wolff ASB Oftedal BEV Kisand K Ersvær E Lima K Husebye ES. Flow cytometry study of blood cell subtypes reflects autoimmune and inflammatory processes in autoimmune polyendocrine syndrome type I. Scand J Immunol. (2010) 71:459–67. 10.1111/j.1365-3083.2010.02397.x 20500699

[334] Fujii Y Kato N Kito J Asai J Yokochi T. Experimental autoimmune adrenalitis: a murine model for Addison's disease. Autoimmunity. (1992) 12:47–52. 10.3109/08916939209146129 1617104

[335] Dudley HR Ritchie AC Schilling A Baker WH. Pathologic changes associated with the use of sodium ethylene diamine tetra-acetate in the treatment of hypercalcemia. Report of two cases with autopsy findings. N Engl J Med. (1955) 252:331–7. 10.1056/NEJM195503032520901 13236025

[336] Craig JM Schiff LH Boone JE. Chronic moniliasis associated with Addison's disease. AMA Am J Dis Child. (1955) 89:669–84. 10.1001/archpedi.1955.02050110809003 14375372

[337] Perlmutter M Ellison RR Norsa L Kantrowitz AR. Idiopathic hypoparathyroidism and Addison's disease. Am J Med. (1956) 21:634–43. 10.1016/0002-9343(56)90078-x 13362293

[338] Whitaker J Landing BH Esselborn VM Williams RR. The syndrome of familial juvenile hypoadrenocorticism, hypoparathyroidism and superficial moniliasis. J Clin Endocrinol Metab. (1956) 16:1374–87. 10.1210/jcem-16-10-1374 13367177

[339] McMahon FG Cookson DU Kabler JD Inhorn SL. Idiopathic hypoparathyroidism and idiopathic adrenal cortical insufficiency occurring with cystic fibrosis of the pancreas. Ann Intern Med. (1959) 51:371–84. 10.7326/0003-4819-51-2-371

[340] Carter AC Kaplan SA DeMayo AP Rosenblum DJ. An unusual case of idiopathic hypoparathyroidism, adrenal insufficiency, hypothyroidism and metastatic calcification. J Clin Endocrinol Metab. (1959) 19:1633–41. 10.1210/jcem-19-12-1633 13808009

[341] Petri M Nerup J. Addison's adrenalitis. Studies on diffuse lymphocytic adrenalitis (idiopathic Addison's disease) and focal lymphocytic infiltration in a control material. Acta Pathol Microbiol Scand A. (1971) 79:381–8. 10.1111/j.1699-0463.1971.tb01835.x 5132054

[342] Rottembourg D Le Deist F Carel JC Mallone R Caillat-Zucman S Deal C. Autoreactive T cells in patients with autoimmune adrenal diseases. Horm Res. (2009) 72(S3):217. 10.1159/000239668

[343] Rottembourg D Deal C Lambert M Mallone R Carel JC Lacroix A. 21-Hydroxylase epitopes are targeted by CD8 T cells in autoimmune Addison's disease. J Autoimmun. (2010) 35:309–15. 10.1016/j.jaut.2010.07.001 20685079

