# Supplementary material for: Autoimmune Addison's Disease as Part of the Autoimmune Polyglandular Syndrome Type 1: Historical Overview and Current Evidence
Source: Front Immunol. 2021 Feb 26;12:606860. doi: 10.3389/fimmu.2021.606860 (PMC7953157; doi:10.3389/fimmu.2021.606860)
Supplement: Supplementary file 1 [file Table_1.pdf]

Table S1. Personal data of APS1 patients reported until 1980. Data given in red indicate subclinical diseases. The asterisk indicates probable decease by acute adrenal failure

Author<br>Year	Gender	Initials	Birth Date	CMC	HPT	AAD	GI	AG	T1D	ATD	M	AH	K	A	V	EH	Last Obs.<br>Death †	Autopsy – Pathologic changes of adrenal cortex – Histology
Thorpe and Handley 1929<br>*Philadelphia (PA), USA*	F	EP	Oct 01, 1921	4	4				5				5				7 †	Not performed
Ostertag 1930<br>*Berlin, Germany*	F	CL			1	2											2 †*	Atrophy and fibrosis – Lymphocyte and mononuclear cell infiltrate
Söderlund 1938<br>*Helsinki, Finland*	M	MH			+				+								21	
Emerson et al. 1941 (updated by Gass 1962)<br>*Baltimore (MD), USA*	M	JH		+	13								4	+		+	18 †	Undisclosed
McQuarrie et al. 1941 (updated by Papadatos and Klein 1954, and DiGeorge and Paschkis 1957) – *Minneapolis (MN), USA*	M	LW			13	19										+	19 †	Complete atrophy – Histology not reported
Sevringhaus 1942 (updated by Sutphin et al. 1943)<br>*Madison (WI), USA*	F	DE		4	5												27	
Sutphin et al. 1943 (updated by Kantrowitz et al. 1969)<br>*Boston (MA), USA*	M▲	AG		0	10												35	
	F▲	LG		2	5												14 †	Not performed
	F▲	LG		4	11	12							+	+			12 †*	Not performed
	F	ER		1	7												7	
	F	SAH		+	3											+	7	
Talbot et al. 1943 (updated by Talbot et al. 1954, Craig et al. 1955, and Fisher and Fitzpatrick 1970) – *Boston (MA), USA*	F▲			+	4	8											10 †*	Not performed
	M▲		Sep 26, 1936	+	6	6						+	+	+			6 †	Atrophy – Lymphocyte and plasma cell infiltrate
	F▲			0	6	14									14		21	
Leonard 1946<br>*New York (NY), USA*	F	EK			3	12							10	+		+	12 †*	Severe atrophy and fibrosis – Lymphocyte infiltrate
Keating 1947 (updated by Lovestedt 1950, and Hermans et al. 1969) – *Rochester (MN), USA*	F▲			2	6								2	+	7	+	45	
	F▲				3	18	13									+	19 †*	Undisclosed
Gotta and Odoriz 1948<br>*Buenos Aires, Argentina*	M	AC		+	16												21	
Berezin and Stein 1948 (updated by Weinberg et al. 1976)<br>*New York (NY), USA*	M▲	ER	Mar 08, 1940	7	4	33	33		33	33				20	28	+	34	
	M▲				+												?	
Collins-Williams 1950 (updated by Craig et al. 1955)<br>*Boston (MA), USA*	M▲		Apr 28, 1940	1	6	6					2			7			11 †*	Severe atrophy – Lymphocyte and plasma cell infiltrate
	M▲	LW		1	6				6		4	4					14	
Salvesen and Böe 1953<br>*Oslo, Norway*	M	LM	Mar 17, 1940	0	7	8					7						8 †	Undisclosed
Leifer and Hollander 1953<br>*New York (NY), USA*	M	PH			8	19					8						20	
McLean 1954<br>*Aberdeen, United Kingdom*	F			+	4												11	

Papadatos and Klein 1954	M	YS			10	10							9	5		+	10	
Pittsburgh (PA), USA																		
Blackburn 1954	F			10	23												31	
Sidney, Australia																		
Castleman and Towne 1954 (updated by Dudley et al. 1955)	F	JG		2	3	4											4 †*	Severe atrophy – Lymphocyte and plasma cell infiltrate
Boston (MA), USA																		
Craig et al. 1955	F		Jun 14, 1941	3	7	7								7			12 †*	Complete atrophy and fibrosis – Mononuclear cell infiltrate
Boston (MA), USA																		
Perlmutter et al. 1956	F	JL			10	27	24			+		+			+	+	30 †	Severe atrophy and fibrosis – Lymphocyte infiltrate
New York (NY), USA																		
Forbes 1956	F	LJH	Nov 11, 1946		4	5							1				8	
Rochester (NY), USA	F	EF	Jul 21, 1941		1	11										+	13 †*	Complete atrophy and fibrosis
	F	KW	Feb 14, 1948	2	4									3		+	6	
Sano et al. 1956	F			3	4	5										+	6	
Sendai, Japan																		
Whitaker et al. 1956	M	NA		2	15	15	14			+	15	+		+		+	15 †	Severe atrophy – Lymphocyte infiltrate
Middletown (OH), USA																		
DiGeorge and Paschkis 1957	?▲					5											5 †	Undisclosed
Philadelphia (PA), USA	?▲				10	8											10	
	?▲			3	6						2						6	
Wilkins 1957 (updated by Gass 1962)	F	AO’M	Jul 12, 1940	+	4			12			3		0	5		+	20	
Baltimore (MD), USA																		
Malloy and Woodruff 1958	M			+	1	5					6						7 †	Not performed
Nashville (TN), USA																		
Järvinen and Latvalahti 1958 (updated by Siurala et al. 1968)	F▲	EE		10	5			32	37		4			10			45	
Helsinki, Finland	M▲				0												0 †	Undisclosed
	F▲				+												3 †	Undisclosed
	M▲			10							+			10			35	
Hetzel and Robson 1958	F			0	10	15	13						12			+	17	
Adelaide, Australia																		
Fargašová 1958 (updated by Fárková 1971)	F	VL	… .., 1945	+	8	11								8		+	16 †*	Severe atrophy – Histology not reported
Olomouc, former Czechoslovakia																		
Akers et al. 1958 (complemented by Irvine et al. 1968, and updated by Kolb et al. 1970) – *Denver (CO), USA*	F	MH	Aug .., 1948		1	12	13				2						20	
Cathala et al. 1958 (updated by Brun 1975, and Cortet et al. 1978) – *Dijon, France*	F	MFC	Apr 29, 1954	2	2	17	13	18									23	
Svane-Knudsen 1959 (updated by Axelsen 1971)	F▲			32	7			+					26				56	
Kolding, Denmark	F▲				+												21 †	Undisclosed
Cramblett et al. 1959	F▲			4		7											7	
Iowa City (IA), USA	M▲			+		+											? †*	Undisclosed
McMahon et al. 1959	F			+	6	19	13				6			7	+		21 †	Severe atrophy – Mononuclear cell infiltrate
Madison (WI), USA																		
Williams and Wood 1959	F				6	7					6	7				+	7 †	Focal atrophy – Lymphocyte infiltrate
London, United Kingdom																		
Szczepańska and Sapiecha 1959	F	ZB	… .., 1952		+	5											6 †	Extreme atrophy – Histology not reported
Warsaw, Poland																		
Anning 1959	M			+	0												14	
Leeds, United Kingdom																		
Prader et al. 1959	M	JCP	Mar 22, 1944	+		12					3						12 †	Atrophy and fibrosis – Lymphocyte infiltrate
Zurich, Switzerland																		
Carter et al. 1959	F	CV			3	4				+				5			6 †	Severe atrophy – Lymphocyte infiltrate
New York (NY), USA																		
Chaptal et al. 1960 (updated by Vayssettes-Vernet 1976)	F▲	EC	Jun 30, 1953	2	3	9											18 †	Undetectable gland
Montpellier, France	F▲	SC	Nov 26, 1956	0	3	14					0						14 †	Atrophy – Lymphocyte infiltrate

Wagner 1960	F▲			+	3								+				11	
Boston (MA), USA	M▲			+		5											6 †	Undetectable gland

**Author Year**	**Gender**	**Initials**	**Birth Date**	**CMC**	**HPT**	**AAD**	**GI**	**AG**	**T1D**	**ATD**	**M**	**AH**	**K**	**A**	**V**	**EH**	**Last Obs. Death †**	**Autopsy – Pathologic changes of adrenal cortex – Histology**
Buzdygan 1961	M▲			3	9	9											9 †*	Severe atrophy – Lymphocyte infiltrate
Chicago (IL), USA	M▲			+													?	
Morse et al. 1961	F▲	MB			18		13	+							+	+	24	
Halifax, Canada	M▲	RB			8	12	14	+			11					+	14	
	F▲	JB			+			+								+	17	
	F▲	SB			+	15		+								+	15	
	M▲	LB			+											+	19	
	F	NL			8	10										+	10	
Pohjola 1962 (updated by Krohn et al. 1974, and Myllärniemi	M▲	JM	… .., 1954	4	4								5	7			20	
and Perheentupa 1978) – *Helsinki, Finland*	M▲	EM	… .., 1960	0	4	11						3	5			+	17	
Gass 1962	M	FC		4	22	27							4			+	27	
Baltimore (MD), USA																		
Kunin et al. 1963 (updated by Wuepper et al. 1969)	M	WF		+	10	11	14	+			9	+				+	25	
Burlington (VT), USA																		
Visakorpi and Gerber 1963 (updated by Krohn et al. 1974, and	F	SS	… .., 1956	4	5	11		10			4					+	19	
Myllärniemi and Perheentupa 1978) – *Helsinki, Finland*																		
Hung et al. 1963 (updated by Quinto et al. 1964)	M▲	FO	… .., 1942			5											5 †*	Complete atrophy and fibrosis
Washington (DC), USA	M▲	MO				3											14	
	F▲	FO		3	3	7		10									10	
	M▲	JO		16													22	
Shuster 1963 (updated by Lehner 1964, and Lehner et al. 1972)	M▲	MS		14	8	+										+	24	
Newcastle upon Tyne, United Kingdom	M▲					11											21	
Páez Allende and Imbert 1963	F	IRU		+	+											+	12	
Santa Fe, Argentina																		
Birk and Mlczoch 1963	M▲	JK	Dec 12, 1938	0	9	18					7	6		9		+	22	
Vienna, Austria	M▲			5													6 †	Undisclosed
	M▲		… .., 1940	6	6	9					5					+	10 †	Undisclosed
Ikkala et al. 1964 (updated by Siurala et al. 1968)	M	MN	… .., 1932		4		14	+				17					32	
Helsinki, Finland																		
Hiekkala 1964 (updated by Krohn et al. 1974, and Myllärniemi	F▲	HS	… .., 1952	4	4	5	13									+	23	
and Perheentupa 1978) – *Helsinki, Finland*	F▲	TS	… .., 1953	4	4											+	22	
	F▲	PS	… .., 1956	0	4	7											19	
	F▲	MS	… .., 1956	0	6	7											19	
	M▲		… .., 1949			6											6 †*	Not performed
Kuske and Krebs 1964 (updated by Zuppinger et al. 1966, and	M	MB	… .., 1950	0	5			13			+						20	
Krebs and Kuske 1970) – *Bern, Switzerland*																		
Braun 1964	M				8	8					2						11 †	Undetectable gland
Erfurt, former West Germany																		
Kenny and Holliday 1964 (updated by Vazquez and Kenny	F▲	CA	Jul 16, 1947	6	6	8	13	18	22	16	0			+			22	
1973) – *Pittsburgh (PA), USA*	F▲	AA		5	5		15			15							16	
Stickler et al. 1965	M			+	9	10			7		7	+					10	
Rochester (MN), USA																		
Coen and Mazzuoli 1965	F	GO		15	15	15										+	21	
Rome, Italy																		
Morais et al. 1965	F	NT		6	11	11										+	13	
Quebec City, Canada																		

Galusha and Kelly 1965	F▲			0	3								+	+			17	
Charlotte (NC), USA	F▲					0											0 †*	Undisclosed
Marchenko et al. 1965<br>*Moscow, former USSR*	F	OI		1	3	4					0					+	4	
Sjöberg 1966	M▲			6	14						14					+	18	
Mölndal, Sweden	F▲			2	18		13									+	25	
	F			+	12			22							+	+	22	
Sweetnam 1966	F▲				7	7											7	
Huddersfield, United Kingdom	F▲				4	4											4	
Pisanty 1966 (updated by Moshkowitz et al. 1969, and Pisanty and Garfunkel 1977) – *Jerusalem, Israel*	F	MH		2	0						8			+		+	10	
Conte et al. 1967 (updated by Panizon 1969)	F▲	GC	… .., 1950	6	13									10			18	
Padua, Italy	F▲	ALC	May .., 1951	0	8	17	13				0	4	+	+	+		17	
	F▲	EC	… .., 1955	+	3	10					0	4		+			13	
Wuepper and Fudenberg 1967 (updated by Kirkpatrick et al. 1971) – *San Francisco (CA), USA*	M	GR		2		19	14	20	+	20	+	18	19			+	24 †*	Undisclosed
Jackson and Whyte 1967<br>*Glasgow, United Kingdom*	M				17	25											29	
Brodehl et al. 1967	F▲	KK	… .., 1961	+	+												4	
Bonn, former West Germany	M▲				9												9 †	Undisclosed
	M▲				0												0 †	Undisclosed
Chilgren et al. 1967	M			13		17											23	
Minneapolis (MN), USA	M▲			0													25	
	F▲			14	9			9			9						17	
	F▲			8	8												13	
Mariani et al. 1967 (updated by Mariani et al. 1971)	M▲	JPT			5	8											8 †*	Undisclosed
Marseille, France	F▲	BT			6	6											6	
Suchnicka 1967<br>*Wroclaw, Poland*	F			7	16		15										16	
Austoni et al. 1968 (updated by Del Prete et al. 1975)<br>*Padua, Italy*	F	MTF		+	7	18	21	25	25								25	
Sethi et al. 1968<br>*New Dehli, India*	M	MN		+	8											+	14	
Golonka and Goodman 1968<br>*Albany (NY), USA*	F			+	3	17	13										20	
Vukadinović et al. 1968<br>*Zagreb, former Yugoslavia*	F	BT		6	7			+			6	2					12	
Panizon 1969<br>*Trieste, Italy*	F	PH	Dec 19, 1964	0	2						3						4	
Hermans et al. 1969<br>*Rochester (MN), USA*	F			4		29	22							21			33 †	Severe atrophy – Lymphocyte infiltrate
Quichaud et al. 1969 (complemented by Massy 1969)	M▲	JBH		9	9	9					9					+	13	
Amiens, France	F▲	MH		0	0												4 †	Undisclosed
Clarke 1969<br>*Melbourne, Australia*	M	ST		2	10	16											19	
Riley 1969<br>*Danville (PA), USA*	F			+	6						+					+	13	
Goudie et al. 1969<br>*Glasgow, United Kingdom*	M			9	3	22					19					+	22	
Haddock and Blizzard (reported by Irvine 1969 and complemented by Irvine et al. 1969) – *San Juan (PR), USA*	F				2	19											20	
Alteraş et al. 1969<br>*Bucharest, Romania*	F			+		14	13										14	
Greenberg et al. 1969	M			0	11								7	+		+	17	

Author Year	Gender	Initials	Birth Date	CMC	HPT	AAD	GI	AG	T1D	ATD	M	AH	K	A	V	EH	Last Obs. Death †	Autopsy – Pathologic changes of adrenal cortex – Histology
Philadelphia (PA), USA																		
Peillon et al. 1970 *Reims, France*	M	DD	… .., 1955	13	13	12	14							+			14	
Meehan 1970 (updated by Mackey 1975) *Dublin, Ireland*	M		… .., 1954	0		18		+			17						18	
Kirkpatrick et al. 1970 (updated by Kirkpatrick et al. 1971) *Kansas City (KS), USA*	M▲	WM		2		8					17						18	
	M▲	RM		3		8					7						16	
Drury 1970 (complemented by Irvine et al. 1968) *Dublin, Ireland*	F▲	ML	Jan 12, 1945	7	16	15	13	21		15				5			24 †*	Undetectable gland
	M▲	SL	… .., 1947	7		11					7			11			11 †*	Extreme atrophy – Histology not reported
Krebs and Kuske 1970 *Bern, Switzerland*	F▲	RA	… .., 1962	2	2									6		+	8	
	M▲	BA	… .., 1966	0	4									2			4	
Foz et al. 1970 (updated by Foz et al. 1973) *Barcelona, Spain*	F▲	AAP			24	22											26	
	F▲	EAP		+		12											14	
Windorfer 1970 *Freiburg in Brisgow, former West Germany*	M	WL	Jun 12, 1959	0	9	10						2					10	
Nally 1970 *Dublin, Ireland*	F▲			0	2			13					+			+	22	
	M▲				6	17			17								17	
Castanier et al. 1970 *Metz, France*	F		Sep 19, 1964	0	4												5	
Ferchiou et al. 1970 *Tunis, Tunisia*	M				0	11											11	
Irvine 1970 (complemented by Irvine et al. 1968) *London, United Kingdom*	F			19		13	18							19			26	
Block et al. 1971 *Chicago (IL), USA*	F	JW		22	7	23	16										29	
Fields et al. 1971 *Concord (MA), USA*	M			2	14									14	14		15	
Blizzard 1971 *Baltimore (MD), USA*	F	SW	… .., 1955		1	14					+		+			+	14	
Fárková 1971 *Olomouc, former Czechoslovakia*	F	HH	… .., 1952	+	5	13								5		+	16 †*	Undisclosed
	M	JL	… .., 1955	+	10											+	16	
Money et al. 1971 *Baltimore (MD), USA*	M▲		Oct 23, 1949	3	5	5					0		+	16			19	
	M▲		Mar 03, 1951	8	4	7			16		4		+		7		18	
	M▲		Sep 27, 1956	2	7	0											12	
Castells et al. 1971 *New York (NY), USA*	F▲	MC		6	7	7										+	7	
	M▲	MC		+		6											6	
	F▲	MC		+		4											4	
Levy et al. 1971 (updated by Arvanitakis and Knouss 1973) *Madison (WI), USA*	M			3		8		+			+						17	
Kössling and Emmrich 1971 *Mainz, former West Germany*	M				2	11											12 †*	Complete atrophy and fibrosis – Lymphocyte infiltrate
Stankler and Bewsher 1972 *Aberdeen, United Kingdom*	F▲	HC	… .., 1951	+	15		13				3	3					18	
	F▲	KC	… .., 1951	1	18									+			18	
	M▲	DC	… .., 1955	8	14												14	
Témime 1972 *Marseille, France*	F			5	10												11	
Olin and Poindexter 1972 *Fargo (ND), USA*	F▲		Oct 30, 1951	+	6	19		11								+	19	
	F▲		May 11, 1953	10	4								+				17	

Rameis et al. 1972	M▲	HO	May 27, 1953	3	3											+	18	
Innsbruck, Austria	F▲	EO	Feb 03, 1955	3	3		13				12					+	16	
	F	IB	Oct 02, 1967	1	0												3	
	F	SF	Aug 19, 1962	5	3	5					1		5				7	
Breynaert et al. 1973 (complemented by Dumortier-Iscovich	M▲	TF	Jun 28, 1962	5	7								2				10	
1973) – *Lille, France*	M▲	BF	Mar 25, 1957	0		14							10				16	
Mitschke et al. 1973	F			5	7	7				7		5					7 †	Severe atrophy and fibrosis – Lymphocyte and
Hamburg, former West Germany																		macrophage infiltrate
Arvanitakis and Knouss 1973	M▲			7	14	23	+	23								+	23	
Madison (WI), USA	M▲			5		8											8 †*	Undisclosed
Scott 1973	F				16	+											16	
Craigavon, United Kingdom																		
Hooper et al. 1973	M▲				3												24	
Sidney, Australia	M▲					12											12	
Corvaglia 1973	F▲	LM		+	2	5											5	
Florence, Italy	F▲	AM		+	6	8											8	
Wong and Kirkpatrick 1973 (complemented by Kirkpatrick et	M	RH		0	7								+	+			17	
al. 1971) – *Washington (DC), USA*																		
Langslet et al. 1974	M			+	+	+											7	
Oslo, Norway																		
Marieb et al. 1974	F			+	12	13					12						13	
Boston (MA), USA																		
Kleerekoper et al. 1974	F		Oct 28, 1956	13	5		16							8		+	17	
Sidney, Australia																		
Krohn et al. 1974 (updated by Myllärniemi and Perheentupa	F	RH	… .., 1959	12	12	12										+	14	
1978) – *Helsinki, Finland*	F	VT	… .., 1965	1	1	6										+	10	
	F	LP	… .., 1961	6	6	12		+			6						12	
	F	LR	… .., 1965	1	2	8							1			+	11	
	M▲	SA	… .., 1961	2		9								7		+	14	
	F	KL	… .., 1963	1	3	9				9						+	12	
	M	TW		8	8							10					10 †	Undisclosed
	M▼	TM	… .., 1963	8	1											+	12	
	F▼	NM	… .., 1967	2	1	5										+	8	
	M▼	KM	… .., 1961	3	4	4										+	14	
	F	MK		1	11												11	
	F	HL	… .., 1967	3	3											+	8	
	F	AN	… .., 1966	2	6								2				8	
	M▲	PA	… .., 1966	1	6												10	
Conte et al. 1974	F▲	EB	… .., 1929	33	26												43	
Padua, Italy	M▲	EB	… .., 1934	+	36												38	
Jehanne and Guivarch 1974	F	GB	Mar 10, 1955	2	2		13				0			10			16	
Roscoff, France																		
Bovier-Lapierre and Jeune 1974	M	DP		+	3	3						5					5 †	Undisclosed
Lyon, France																		
Schulkind and Ayoub 1975	F			1	3			+							3		12	
Gainesville (FL), USA																		
Kaffe et al. 1975	F▲	MC		7	7												12	
New York (NY), USA	M▲	MC		+	10											+	10	
	F▲	MC		4	8							7					8	
Ellwood et al. (reported by Irvine and Barnes 1975)	F▲			+	9	10	13					8	+	15			15	
Bristol, United Kingdom	M▲				7							7	0				13	
Joksimović et al. 1975	M▲	RS	Jun 17, 1956	2							3	2					3 †	Not reported
Belgrade, former Yugoslavia	F▲	GS	Dec 11, 1960	3	4						4	6					6 †	Not performed
	F▲	SS	Sep 09, 1964		2	3					1	3					3 †*	Severe atrophy – Lymphocyte infiltrate

	M▲	VS	Feb 05, 1969		2					1						2	
	F	BB	Apr 04, 1964	4	3	5				4	4	4				6 †*	Not performed
	M	RT	Jun 12, 1954		7	7				12	12					19	
Petković 1975	M			5		15						7	7			23	
Zagreb, former Yugoslavia																	
Champenois 1975	F	NP	Mar 29, 1969	3	3					3						6	
Lille, France																	
Heinonen et al. 1976	M	JK	… .., 1946	0		19		20		17						26	
Tampere, Finland																	
Lawton et al. 1976 (updated by Kessel and Taylor 1980)	M▲	KH	Aug 14, 1968	0	4											9	
Edinburgh, United Kingdom	M▲	MH	Oct 11, 1975	0												2	
Chesney et al. 1976	M	PR		3	13	14							6			16	
Montreal, Canada																	
Matsumoto et al. 1976	M			+	+	+										16	
Fukuoka, Japan	F			4	12											14	
Farrell et al. 1976	F			2	6	9										9	
Madison (WI), USA																	
Blake 1976	F			+	3	18										25	
Dublin, Ireland	F			+	18							+				22	
Vayssettes-Vernet 1976	F	PP	Dec 04, 1962	11	11	11	13									13	
Grenoble, France																	
Stieglitz et al. 1977	F▲				2							4			+	15	
Toronto, Canada	F				+							9			+	17	
	M			+	+	+							+	+		10	
	M			+	+	+					+					6	
	M▲				+	+										19	
	F				+	+										5	
Lucky et al. 1977	F			0	4	11				0			4			17	
Bethesda (MD), USA	F▲			7	8		14									14	
	M▲			+		+			+				+	+		11	
Hertz et al. 1977	F			6	9	34	15	13					6	28		34	
Bethesda (MD), USA	F			3	6	10	16						3	3		18	
Chesney et al. 1977	F		… .., 1962	12	7					+	7					14	
Madison (WI), USA																	
Pisanty and Garfunkel 1977	F▲	VA		9	18					+		+	+			18	
Jerusalem, Israel	M▲	IA		8									+			21	
	F	ZS		+	17								+		+	18	
	M	IA		+	+								+		+	17	
	M▼	NS		+	7								+		+	30	
	M▼			+	+										+	?	
Rebollar et al. 1977	F			+	21											34	
Madrid, Spain																	
Miani and Rumi 1977	M	FG		6	+	+										14	
Rome, Italy																	
Dučić and Terzić-Kazić 1978	M▲	BM		+	8	11									+	11	
Sarajevo, former Yugoslavia	F▲			+												9	
Prader 1978	M▲			+						+						3 †	Undisclosed
Zurich, Switzerland	M▲			+	+					+						7 †	Undisclosed
	M▲				+	+										3 †	Atrophy – Histology not reported
	M▲				2					2						8 †	Undisclosed
Myllärniemi and Perheentupa 1978	F	MH		0	4										+	5	
Helsinki, Finland	F	AR		10	5	10									+	11	
	F	MK			6	7									+	8	
	F	MP		3	3	9									+	14	

	F	SK		7	23	23										+	23	
	M	MP		5	8											+	11	
	M	HK		10	10	10										+	17	
	M	KK		7	18											+	22	
Brenton et al. 1978<br>*Colchester, United Kingdom*	M		… .., 1940	+	34						+						36	
Tomar et al. 1979<br>*Syracuse (NY), USA*	F		… .., 1955	10	2	20	13				17			10			20	
Betterle et al. 1979<br>*Padua, Italy*	F			+	+	+		+							+		25	
Matzner et al. 1979<br>*Jerusalem, Israel*	F				3	15		15							10		16	
Arulanantham et al. 1979<br>*New Haven (CT), USA*	M▲			7	7						+			3			12	
	F▲			5	4	4											6	
	F▲			2		2						2					3 †	Not reported
Ivanova and Ignatovich 1979<br>*Omsk, former USSR*	M			2	5						3					+	9	
Myers et al. 1980<br>*Hartford (CT), USA*	F▲				2	15	13										26	
	M▲					6											6 †*	Undisclosed
Adami Lami et al. 1980<br>*Florence, Italy*	F▲	CS		0	8	13	13								+	+	13	
	M▲			+													13	
Clifton-Bligh et al. 1980<br>*Sidney, Australia*	M	DOB	Jul 21, 1952	0	10	22									+		25	
Rico Zalba and Calderin 1980<br>*Madrid, Spain*	M▲					+											? †*	Undisclosed
	M▲	DVC		+	8	14					+					+	14	
	F▲	FVC		+	11						2						19	
	M▲	SVC		13													14	

CMC, chronic mucocutaneous candidiasis; HPT, idiopathic hypoparathyroidism; AAD, autoimmune Addison’s disease; GI, gonadal insufficiency; AG, atrophic gastritis; T1D, type-1 diabetes; ATD, autoimmune thyroid disease; M, malabsorption; AH, autoimmune hepatitis; K, keratitis; A, alopecia; V, vitiligo; EH, enamel hypoplasia

References

1. Thorpe ES Jr, Handley HE. Chronic tetany and chronic mycelial stomatitis in a child aged four and one-half years. *Am J Dis Child*. (1929) 38:328–38. doi: 10.1001/archpedi.1929.01930080104011

2. Ostertag B. Die an bestimmte Lokalisation gebundenen Konkremente des Zentralnervensystems und ihre Beziehung zur "Verkalkung intracelebraler Getäße" beigewissen endokrinen Erkrankungen. *Virchows Arch Pathol Anat Physiol Klin Med*. (1930) 275:828–59. doi: 10.1007/BF01947423

3. Söderlund S. Några fall av tetani, behandlade med A.T. 10. *Fin Lakaresallsk Handl*. (1938) 81:659–87.

4. Emerson K, Walsh FB, Howard JE. Idiopathic hypoparathyroidism: a report of 2 cases. *Ann Intern Med*. (1941) 14:1256–70. doi: 10.7326/0003-4819-14-7-1256

5. McQuarrie I, Hansen AE, Ziegler MR. Studies on the convulsive mechanism in idiopathic hypoparathyroidism. *J Clin Endocrinol*. (1941) 1:789–98. doi: 10.1210/jcem-1-10-789

6. Sevringhaus EL. Activated sterols and calcium salts in treatment of parathyroid tetany. *Am J Med Sci*. (1942) 203:726–31.

7. Sutphin A, Albright F, McCune DJ. Five cases (three in siblings) of idiopathic hypoparathyroidism associated with moniliasis. *J Clin Endocrinol*. (1943) 3:625–34. doi: 10.1210/jcem-3-12-625

8. Sevringhaus EL, St. John R. Parathyroid tetany treated with massive doses of vitamin D. *J Clin Endocrinol*. (1943) 3:635–7. doi: 10.1210/jcem-3-12-635

9. Talbot NB, Butler AM, MacLachlan EA. The effect of testosterone and allied compounds on the mineral, nitrogen, and carbohydrate metabolism of a girl with Addison's disease. *J Clin Invest*. (1943) 22:583–93. doi: 10.1172/JCI101430

10. Leonard MF. Chronic idiopathic hypoparathyroidism with superimposed Addison's disease in a child. *J Clin Endocrinol*. (1946) 6:493–506. doi: 10.1210/jcem-6-7-493

11. Keating FR Jr. The normal and pathologic physiology of the parathyroid glands. *Am J Orthod Oral Surg*. (1947) 33:B129–37. doi: 10.1016/s0096-6347(47)90046-x

12. Gotta H, Odoriz JB. The electroencephalogram in hypoparathyroidism with tetany and epilepsy. *J Clin Endocrinol*. (1948) 8:674–86. doi: 10.1210/jcem-8-8-674

13. Berezin SW, Stein JD. Idiopathic hypoparathyroidism. A case simulating epilepsy and brain tumor. *J Pediatr*. (1948) 33:346–51. doi: 10.1016/s0022-3476(48)80112-5

14. Lovestedt SA. The dental picture of spontaneous parathyroid insufficiency. *Oral Surg Oral Med Oral Pathol*. (1950) 3:396–402. doi: 10.1016/0030-4220(50)90337-9

15. Collins-Williams C. Idiopathic hypoparathyroidism with papilledema in a boy six years of age. Report of a case associated with moniliasis and the celiac syndrome and a brief review of the literature. *Pediatrics*. (1950) 5:998–1007.

16. Gotta H. Tetany and epilepsy. *AMA Arch Neurol Psychiatry*. (1951) 66:714–21. doi: 10.1001/archneurpsyc.1951.02320120047006

17. Salvesen HA, Böe J. Idiopathic hypoparathyroidism. Observations on two cases, one complicated by moniliasis and idiopathic steatorrhea and one with an unusual degree of calcium deposition in the bones. *Acta Endocrinol (Copenh)*. (1953) 14:214–26. doi: 10.1530/acta.0.0140214

18. Salvesen HA, Böe J. Osteomalacia in sprue. *Acta Med Scand*. (1953) 146:290–9. doi:

10.1111/j.0954-6820.1953.tb10243.x

19. Papadatos C, Klein R. Addison's diseases developing in a boy with hypoparathyroidism. *AMA Am J Dis Child*. (1953) 86:336–7. doi: 10.1001/archpedi.1953.02050080329008

20. Walsh FB, Murray RG. Ocular manifestations of disturbances in calcium metabolism. *Am J Ophthalmol*. (1953) 36:1657–76. doi: 10.1016/0002-9394(53)90001-4

21. Leifer E, Hollander W Jr. Idiopathic hypoparathyroidism and chronic adrenal insufficiency: a case report. *J Clin Endocrinol Metab*. (1953) 13:1264–9. doi: 10.1210/jcem-13-10-1264

22. McLean MM. Chronic idiopathic hypoparathyroidism associated with moniliasis. *Arch Dis Child*. (1954) 29:419–21. doi: 10.1136/adc.29.147.419

23. Papadatos C, Klein R. Addison's disease in a boy with hypoparathyroidism. *J Clin Endocrinol Metab*. (1954) 14:653–60. doi: 10.1210/jcem-14-6-653

24. Blackburn CRB. The management of chronic hypoparathyroid tetany. *Med J Aust*. (1954) 1:928–32. doi: 10.5694/j.1326-5377.1954.tb110394.x

25. Castleman B, Towne VW. Case records of the Massachusetts General Hospital. Weekly clinicopathological exercises. Case 40361. *N Engl J Med*. (1954) 251:442–8. doi: 10.1056/NEJM195409092511109

26. Talbot NB, Sobel EH, McArthur JW, Crawford JD. The parathyroids. In: *Functional endocrinology from birth through adolescence*. Cambridge (MA), USA: Harvard University Press. (1954). p. 52–133. doi: 10.4159/harvard.9780674335677

27. Talbot NB, Sobel EH, McArthur JW, Crawford JD. The adrenal cortices. In: *Functional endocrinology from birth through adolescence*. Cambridge (MA), USA: Harvard University Press. (1954). p. 134–268. doi: 10.4159/harvard.9780674335677

28. Craig JM, Schiff LH, Boone JE. Chronic moniliasis associated with Addison's disease. *AMA Am J Dis Child*. (1955) 89:669–84. doi: 10.1001/archpedi.1955.02050110809003

29. Dudley HR, Ritchie AC, Schilling A, Baker WH. Pathologic changes associated with the use of sodium ethylene diamine tetra-acetate in the treatment of hypercalcemia. Report of two cases with autopsy findings. *N Engl J Med*. (1955) 252:331–7. doi: 10.1056/NEJM195503032520901

30. Perlmutter M, Ellison RR, Norsa L, Kantrowitz AR. Idiopathic hypoparathyroidism and Addison's disease. *Am J Med*. (1956) 21:634–43. doi: 10.1016/0002-9343(56)90078-x

31. Forbes GB. Clinical features of idiopathic hypoparathyroidism in children. *Ann N Y Acad Sci*. (1956) 64:432–55. doi: 10.1111/j.1749-6632.1956.tb52465.x

32. Sano T, Nihei J, Suzuki M, Sato N. Chronischer idiopatischer Hypoparathyreoidismus mit Moniliasis. *Helv Paediatr Acta*. (1956) 11:653–9.

33. Whitaker J, Landing BH, Esselborn VM, Williams RR. The syndrome of familial juvenile hypoadrenocorticism, hypoparathyroidism and superficial moniliasis. *J Clin Endocrinol Metab*. (1956) 16:1374–87. doi: 10.1210/jcem-16-10-1374

34. Hinrichs EH Jr. Dental changes in idiopathic juvenile hypoparathyroidism. *Oral Surg Oral Med Oral Pathol*. (1956) 9:1102–14. doi: 10.1016/0030-4220(56)90073-1

35. Harrison HE. Idiopathic hypoparathyroidism. *Pediatrics*. (1956) 17:442–8.

36. DiGeorge AM, Paschkis K. The syndrome of Addison's disease, hypoparathyroidism, and superficial moniliasis. *AMA J Dis Child*. (1957) 94:476–8. doi:

10.1001/archpedi.1957.04030060008003

37. O'Donovan DK. Hypoparathyroidism and moniliasis. *Ir J Med Sci*. (1957) 378:255–7. doi: 10.1007/BF02954478

38. Wilkins L. Calcium and phosphorus metabolism – Parathyroid disorders and tetany. In: *The diagnosis and treatment of endocrine disorders in childhood and adolescence. Second Edition*. Oxford, UK: Blackwell Scientific Publications. (1957). p. 407–40.

39. Malloy BM, Woodruff CW. Addison's disease in three six-year-old boys. *AMA J Dis Child*. (1958) 95:364–9. doi: 10.1001/archpedi.1958.02060050366004

40. Järvinen KAJ, Latvalahti J. Some observations of nontropical sprue. A case with hypocalcemia, intrinsic factor deficiency, anemia and diabetes mellitus. *Ann Med Intern Fenn*. (1958) 47:39–46.

41. Hetzel BS, Robson HN. The syndrome of hypoparathyroidism, Addison's disease and moniliasis. *Australas Ann Med*. (1958) 7:27–33. doi: 10.1111/imj.1958.7.1.27

42. Fargašová I. Primární idiopatický hypoparatyreoidismus a Addisonova nemoc u 12letého děvčete. *Cesk Pediatr*. (1958) 13:539–43.

43. Akers DR, Binkley EL, Miller AP. Homotransplantation of parathyroid gland in idiopathic hypoparathyroidism. *Pediatrics*. (1958) 21:973–9.

44. Cathala J, Polonovski C, Barre C. L'hypoparathyroïdie chronique primitive de l'enfant. *Presse Med*. (1958) 66:1925–8.

45. Svane-Knudsen P. Severe secondary ocular changes in a patient suffering from idiopathic hypoparathyroidism and pernicious anaemia. *Acta Ophthalmol (Copenh)*. (1959) 37:560–7. doi: 10.1111/j.1755-3768.1959.tb03469.x

46. Cramblett HG, Moffet H, Najjar S. Experiences with amphotericin B in treatment of histoplasmosis and moniliasis. *AMA J Dis Child*. (1959) 98:509–10. doi: 10.1001/archpedi.1959.02070010497016

47. McMahon FG, Cookson DU, Kabler JD, Inhorn SL. Idiopathic hypoparathyroidism and idiopathic adrenal cortical insufficiency occurring with cystic fibrosis of the pancreas. *Ann Intern Med*. (1959) 51:371–84. doi: 10.7326/0003-4819-51-2-371

48. Williams E, Wood C. The syndrome of hypoparathyroidism and steatorrhoea. *Arch Dis Child*. (1959) 34:302–6. doi: 10.1136/adc.34.176.302

49. Szczepańska H, Sapiecha J. Addison's disease with hypofunction of the parathyroid glands. *Arch Dis Child*. (1959) 34:498–500. doi: 10.1136/adc.34.178.498

50. Anning ST. Spontaneous hypoparathyroidism with generalized moniliasis. *Br J Dermatol*. (1959) 71:75–6. doi: 10.1111/j.1365-2133.1959.tb13388.x

51. McIntyre PA, Hahn R, Conley CL, Glass B. Genetic factors in predisposition to pernicious anemia. *Bull Johns Hopkins Hosp*. (1959) 104:309–42.

52. Prader A, Uehlinger E, Illig R. Hypercalcämie bei Morbus Addison im Kindesalter. *Helv Paediatr Acta*. (1959) 14:607–17.

53. Carter AC, Kaplan SA, DeMayo AP, Rosenblum DJ. An unusual case of idiopathic hypoparathyroidism, adrenal insufficiency, hypothyroidism and metastatic calcification. *J Clin Endocrinol Metab*. (1959) 19:1633–41. doi: 10.1210/jcem-19-12-1633

54. Chaptal J, Jean R, Bonnet H, Guillaumot R, Morel G. Hypoparathyroïdie familiale. Études clinique, biologique et thérapeutique. *Arch Fr Pediatr*. (1960) 17:866–78.

55. Wagner R. The syndrome of chronic hypoparathyroidism, Addison's disease and superficial moniliasis. *Exp Med Surg*. (1960) 18:157–60.

56. Buzdygan D, Lepper M, Neuhauser I, Rosenthal IM. Extensive cutaneous moniliasis. Treatment with amphotericin B. *Am J Dis Child*. (1961) 102:168–79. doi: 10.1001/archpedi.1961.02080010170004

57. Morse WI, Cochrane WA, Landrigan PL. Familial hypoparathyroidism with pernicious anemia, steatorrhea and adrenocortical insufficiency. A variant of mucoviscidosis. *N Engl J Med*. (1961) 264:1021–6. doi: 10.1056/NEJM196105182642003

58. Pohjola S. Ocular manifestations of idiopathic hypoparathyroidism. Case report and review of literature. *Acta Ophthalmol (Copenh)*. (1962) 40:255–65. doi: 10.1111/j.1755-3768.1962.tb02365.x

59. Gass JDMcI. The syndrome of keratoconjunctivitis, superficial moniliasis, idiopathic hypoparathyroidism and Addison's disease. *Am J Ophthalmol*. (1962) 54:660–74. doi: 10.1016/0002-9394(62)92198-0

60. Blizzard RM, Kyle MA, Chandler RW, Hung W. Adrenal antibodies in Addison's disease. *Lancet*. (1962) 280:901–3. doi: 10.1016/s0140-6736(62)90681-5

61. Conti C, Sereno L. Adrenal antibodies in Addison's disease. *Lancet*. (1962) 280:1228. doi: 10.1016/S0140-6736(62)91004-8

62. Kunin AS, MacKay BR, Burns SL, Halberstam MJ. The syndrome of hypoparathyroidism and adrenocortical insufficiency, a possible sequel of hepatitis. Case report and review of the literature. *Am J Med*. (1963) 34:856–66. doi: 10.1016/0002-9343(63)90092-5

63. Visakorpi JK, Gerber M. Hypoparathyroidism with steatorrhoea and some features of pernicious anaemia in a 5-year-old girl. *Ann Paediatr Fenn*. (1963) 9:128–37.

64. Blizzard RM, Kyle M. Studies of the adrenal antigens and antibodies in Addison's disease. *J Clin Invest*. (1963) 42:1653–60. doi: 10.1172/JCI104851

65. Hung W, Migeon CJ, Parrott RH. A possible autoimmune basis for Addison's disease in three siblings, one with idiopathic hypoparathyroidism, pernicious anemia and superficial moniliasis. *N Engl J Med*. (1963) 269:658–63. doi: 10.1056/NEJM196309262691303

66. Shuster S. Moniliasis with idiopathic hypoparathyroidism. *Proc R Soc Med*. (1963) 56:302–3.

67. Páez Allende F, Imbert O. Catarata infantil por hipoparatiroidismo espontaneo. *Rev Asoc Med Argent*. (1963) 77:461–3.

68. Birk W, Mlczoch F. Zur Problematik des Syndroms von familiärer Moniliasis, Morbus Addison und Hypoparathyreoidismus. *Wien Z Inn Med*. (1963) 44:520–7.

69. Ikkala E, Siurala M, Viranko M. Hypoparathyroidism and pernicious anaemia. *Acta Med Scand*. (1964) 176:73–7. doi: 10.1111/j.0954-6820.1964.tb00647.x

70. Hiekkala H. Idiopathic hypoparathyroidism, adrenal insufficiency and moniliasis in children. *Ann Paediatr Fenn*. (1964) 10:213–22.

71. Kuske H, Krebs A. Therapieresistente Soormykose bei kongenitalem Hypoparathyreoidismus. *Dermatologica*. (1964) 129:162–3.

72. Quinto MG, Leikin SL, Hung W. Pernicious anemia in a young girl associated with idiopathic hypoparathyroidism, familial Addison's disease, and moniliasis. *J Pediatr*. (1964) 64:241–7. doi: 10.1016/s0022-3476(64)80268-7

73. Braun W. Das Syndrom Hypocortizismus (Morbus Addison) mit Hypoparathyreoidismus im Kindesalter. *Kinderarztl Prax*. (1964) 33:207–10.

74. Kenny FM, Holliday MA. Hypoparathyroidism, moniliasis, Addison's and Hashimoto's diseases. Hypercalcemia treated with intravenously administered sodium sulfate. *N Engl J Med*. (1964) 271:708–13. doi: 10.1056/NEJM196410012711404

75. Lehner T. Chronic candidiasis. *Trans St Johns Hosp Dermatol Soc*. (1964) 50:8–21.

76. Hiekkala H. Idiopathic hypoparathyroidism, adrenal insufficiency and moniliasis in children. *Acta Endocrinol (Copenh)*. (1965) 49(S101):41.

77. Stickler GB, Peyla TL, Dower JC, Logan GB. Moniliasis, steatorrhea, diabetes mellitus, cirrhosis, gallstones, and hypoparathyroidism in a 10-year-old boy. *Clin Pediatr (Phila)*. (1965) 4:276–85. doi: 10.1177/000992286500400508

78. Coen G, Mazzuoli GF. Idiopathic hypoparathyroidism associated with adrenal insufficiency – Report of a case with a study of calcium metabolism. *Folia Endocrinol*. (1965) 18:136–48.

79. Morais T, Saucier G, Brunet J. Maladie d'Addison et hypoparathyroïdie. *Laval Med*. (1965) 36:732–7.

80. Galusha BL, Kelly LW Jr. Idiopathic hypoparathyroidism associated with chronic moniliasis. A problem in management of repeated severe seizures. *N C Med J*. (1965) 26:441–5.

81. Marchenko LF, Storozhenko OG, Tsyvil'skaia LA. K voprosu o khronicheskom idiopaticheskom gipoparatireoze. *Vopr Okhr Materin Det*. (1965) 10:78–82.

82. Sjöberg K-H. Moniliasis – an internal disease? Three cases of idiopathic hypoparathyroidism with moniliasis, steatorrhea, primary amenorrhea and pernicious anemia. *Acta Med Scand*. (1966) 179:157–66. doi: 10.1111/j.0954-6820.1966.tb05443.x

83. Zuppinger K, Moser H, Tönz O. Idiopatischer Hypoparathyreoidismus und megaloblastäre Anämie. *Ann Paediatr*. (1966) 206:235–6.

84. Blizzard RM, Chee D, Davis W. The incidence of parathyroid and other antibodies in the sera of patients with idiopathic hypoparathyroidism. *Clin Exp Immunol*. (1966) 1:119–28.

85. Blizzard RM, Chee D, Davis WG. Idiopathic hypoparathyroidism: a probable autoimmune disease. *J Pediatr*. (1966) 69:969. doi: 10.1016/S0022-3476(66)80555-3

86. Sweetnam WP. Juvenile familial endocrinopathy. *Lancet*. (1966) 287:463–5. doi: 10.1016/s0140-6736(66)91461-9

87. Goudie RB, Anderson JR, Gray KK, Whyte WG. Autoantibodies in Addison's disease. *Lancet*. (1966) 287:1173–6. doi: 10.1016/S0140-6736(66)91070-1

88. Pisanty S. Primary idiopathic juvenile hypoparathyroidism. *Oral Surg Oral Med Oral Pathol*. (1966) 21:294–8. doi: 10.1016/0030-4220(66)90060-0

89. Conte N, Scandellari C, Macrì C, Ferlin G. La cinetica del Ca^{45} nell'ipoparatiroidismo familiare. *Acta Isot (Padova)*. (1967) 7:315–23.

90. Blizzard RM, Chee D, Davis W. The incidence of adrenal and other antibodies in the sera of patients with idiopathic adrenal insufficiency (Addison's disease). *Clin Exp Immunol*. (1967) 2:19–30.

91. Irvine WJ, Stewart AG, Scarth L. A clinical and immunological study of adrenocortical

insufficiency (Addison's disease). *Clin Exp Immunol*. (1967) 2:31–69.

92. Wuepper KD, Fudenberg HH. Moniliasis, "autoimmune" polyendocrinopathy, and immunologic family study. *Clin Exp Immunol*. (1967) 2:71–82.

93. Hällström T. Familial occurrence of idiopathic hypoparathyroidism. *Hereditas*. (1967) 58:325–32. doi: 10.1111/j.1601-5223.1967.tb02160.x

94. Jackson IMD, Whyte WG. Addison's disease in association with idiopathic hypoparathyroidism. *J Clin Endocrinol Metab*. (1967) 27:348–54. doi: 10.1210/jcem-27-3-348

95. Brodehl J, Gellissen K, Kowalewski S. Isolierter Defekt der tubulären Cystin-Rückresorption in einer Familie mit idiopathischem Hypoparathyreoidismus. *Klin Wochenschr*. (1967) 45:38–40.

96. Chilgren RA, Quie PG, Meuwissen HJ, Hong R. Chronic mucocutaneous candidiasis, deficiency of delayed hypersensitivity, and selective local antibody defect. *Lancet*. (1967) 290:688–93. doi: 10.1016/s0140-6736(67)90974-9

97. Mariani R, Unal D, Favro C, Bernard R. Hypoparathyroïdie chronique d'évolution fatale chez un enfant de 8 ans. *Mars Med*. (1967) 104:1041–5.

98. Charlas J, Paupe J. Le syndrome candidose hypoparathyroïdie chronique idiopathique insuffisance surrénale. *Med Infant (Paris)*. (1967) 74:405–12.

99. Fanconi A. Familiäres Syndrom von Moniliasis, Hypoparathyreoidismus und Nebennierenrindeninsuffizienz. *Schweiz Med Wochenschr*. (1967) 97:1262–3.

100. Suchnicka R. Samoistna niedoczynność przytarczyc okresu pokwitania. *Wiad Lek*. (1967) 20:575–7.

101. Austoni M, Conte N, Scarpa R, Casson F. Ipoparatiroidismo idiopatico associato a insufficienza surrenalica e a gozzo. *Acta Med Patav*. (1968) 28:131–44.

102. Siurala M, Varis K, Lamberg VA. Intestinal absorption and autoimmunity in endocrine disorders. *Acta Med Scand*. (1968) 184:53–64. doi: 10.1111/j.0954-6820.1968.tb02422.x

103. Cawson RA, Lehner T. Chronic hyperplastic candidiasis – Candidal leukoplakia. *Br J Dermatol*. (1968) 80:9–16. doi: 10.1111/j.1365-2133.1968.tb11899.x

104. Anderson JR, Goudie RB, Gray K, Stuart-Smith DA. Immunological features of idiopathic Addison's disease: an antibody to cells producing steroid hormones. *Clin Exp Immunol*. (1968) 3:107–17.

105. Goudie RB, McDonald E, Anderson JR, Gray K. Immunological features of idiopathic Addison's disease: characterization of the adrenocortical antigens. *Clin Exp Immunol*. (1968) 3:119–31.

106. Sethi AS, Verma IC, Mittal SK, Khanna KK. Idiopathic hypoparathyroidism. Report of two cases. *Indian J Pediatr*. (1968) 35:490–5. doi: 10.1007/BF02753123

107. Golonka JE, Goodman AD. Coexistence of primary ovarian insufficiency, primary adrenocortical insufficiency and idiopathic hypoparathyroidism. *J Clin Endocrinol Metab*. (1968) 28:79–82. doi: 10.1210/jcem-28-1-79

108. Spinner MW, Blizzard RM, Childs B. Clinical and genetic heterogeneity in idiopathic Addison's disease and hypoparathyroidism. *J Clin Endocrinol Metab*. (1968) 28:795–804. doi: 10.1210/jcem-28-6-795

109. Vukadinović S, Kačić M, Jezerinac Z. Hipoparatireoidizam s cirozom jetre i steatorejom. *Jugoslav Pedijatr*. (1968) 11:193–203.

110. Irvine WJ, Chan MMW, Scarth L, Kolb FO, Hartog M, Bayliss RIS, et al. Immunological aspects of premature ovarian failure associated with idiopathic Addison's disease. *Lancet*. (1968) 292:883–7. doi: 10.1016/s0140-6736(68)91053-2

111. Blizzard RM, Gibbs JH. Candidiasis: studies pertaining to its association with endocrinopathies and pernicious anemia. *Pediatrics*. (1968) 42:231–7.

112. Irvine WJ. Clinical and immunological associations in adrenal disorders. *Proc R Soc Med*. (1968) 61:271–5.

113. Panizon F. La sindrome "moniliasi superficiale-ipoparatiroidismo". Dimostrazione di un difetto dell'immunità ritardata. *Acta Paediatr Lat*. (1969) 22:1–25.

114. Wuepper KD, Wegienka LC, Fudenberg HH. Immunologic aspects of adrenocortical insufficiency. *Am J Med*. (1969) 46:206–16. doi: 10.1016/0002-9343(69)90005-9

115. Hermans PE, Ulrich JA, Markowitz H. Chronic mucocutaneous candidiasis as a surface expression of deep-seated abnormalities. Report of a syndrome of superficial candidiasis, absence of delayed hypersensitivity and aminoaciduria. *Am J Med*. (1969) 47:503–19. doi: 10.1016/0002-9343(69)90181-8

116. Quichaud J, Le Bozec R, Frison B, Galez A, Massy B, Blanchard J. Syndrome candidose – Hypoparathyroïdie Insuffisance surrénale idiopathique chez un garçon de 10 ans. Considérations nosologiques et étiopathogéniques. *Ann Endocrinol (Paris)*. (1969) 30:682–95.

117. Clarke DM. Chronic candidiasis of the nails, hypoparathyroidism and Addison's disease. *Australas J Dermatol*. (1969) 10:185–6. doi: 10.1111/j.1440-0960.1969.tb01189.x

118. Irvine WJ, Chan MMW, Scarth L. The further characterization of autoantibodies reactive with extra-adrenal steroid-producing cells in patients with adrenal disorders. *Clin Exp Immunol*. (1969) 4:489–503.

119. Irvine WJ, Scarth L. Antibody to the oxyphil cells of the human parathyroid in idiopathic hypoparathyroidism. *Clin Exp Immunol*. (1969) 4:505–10.

120. Spinner MW, Blizzard RM, Gibbs J, Abbey H, Childs B. Familial distributions of organ specific antibodies in the blood of patients with Addison's disease and hypoparathyroidism and their relatives. *Clin Exp Immunol*. (1969) 5:461–8.

121. Kantrowitz PA, Fleischli DJ, Butler WT. Successful treatment of chronic esophageal moniliasis with a viscous suspension of nystatin. *Gastroenterology*. (1969) 57:424–30. doi: 10.1016/S0016-5085(19)33875-2

122. Riley DJ. Dental changes in patients with idiopathic hypoparathyroidism: report of two cases. *J Oral Surg*. (1969) 27:44–7.

123. Goudie RB, Boyle IT, Stuart-Smith DA, Ferguson A. Serological diagnosis of idiopathic Addison's disease in patients on prolonged prednisolone therapy for steatorrhoea. *Lancet*. (1969) 293:186–8. doi: 10.1016/s0140-6736(69)91194-5

124. Vallotton MB, Forbes AP. Premature menopause in autoimmune diseases. *Lancet*. (1969) 293:156–7. doi: 10.1016/s0140-6736(69)91171-4

125. Irvine WJ. Premature menopause in autoimmune diseases. *Lancet*. (1969) 293:264. doi: 10.1016/s0140-6736(69)91280-x

126. Chilgren RA, Meuwissen HJ, Quie PQ, Good RA, Hong R. The cellular immune defect in chronic mucocutaneous candidiasis. *Lancet*. (1969) 293:1286–8. doi: 10.1016/s0140-6736(69)92223-5

127. Alteraş I, Cojocaru I, Bălănescu A. Generalized candidiasis associated with Addison's disease. *Mykosen*. (1969) 12:575–7. doi: 10.1111/j.1439-0507.1969.tb04479.x

128. Greenberg MS, Brightman VJ, Lynch MA, Ship II. Idiopathic hypoparathyroidism, chronic candidiasis, and dental hypoplasia. *Oral Surg Oral Med Oral Pathol*. (1969) 28:42–53. doi: 10.1016/0030-4220(69)90192-3

129. Moshkowitz A, Abrahamov A, Pisanti S. Congenital hypoparathyroidism simulating epilepsy, with other symptoms and dental signs of intra-uterine hypocalcemia. *Pediatrics*. (1969) 44:401–9.

130. Massy B (1969). Le syndrome candidose-hypoparathyroïdie-insuffisance cortico surrénale. *Dissertation Thesis*. University of Amiens, France.

131. Peillon F, Delzant G, Fandre M, Couchot S, Gilbert-Dreyfus, Simon F. Triple syndrome infantile: insuffisance surrénale, hypoparathyroïdie et moniliase. *Ann Med Interne (Paris)*. (1970) 121:231–6.

132. Fischer M, Firzpatrick TB. Candidiasis, vitiligo, Addison's disease, and hypoparathyroidism. *Arch Dermatol*. (1970) 102:110–2. doi: 10.1001/archderm.102.1.110

133. Meehan FO. Chronic moniliasis. *Br J Dermatol*. (1970) 83:707. doi: 10.1111/j.1365-2133.1970.tb15774.x

134. Kirkpatrick CH, Chandler JW, Schimke RN. Chronic mucocutaneous moniliasis with impaired delayed hypersensitivity. *Clin Exp Immunol*. (1970) 6:375–85.

135. Drury MI, Keelan DM, Timoney FJ, Irvine WJ. Juvenile familial endocrinopathy. *Clin Exp Immunol*. (1970) 7:125–32.

136. Krebs A, Kuske H. Chronischer Befall von Mundschleimhaut und Nägeln mit Soor (Candida albicans) als Leitsymptom für primären (idiopathischen) Hypoparathyreoidismus. *Hautarzt*. (1970) 21:400–6.

137. Foz M, Mirada A, Guardia J. Endocrine disorders in a family. *Lancet*. (1970) 296:269.

138. Windorfer A. Kasuistischer Beitrag zum Syndrom Moniliasis, Hypoparathyreoidismus und Morbus Addison. *Monatsschr Kinderheilkd*. (1970) 118:103–5.

139. Nally FF. Idiopathic juvenile hypoparathyroidism with superficial moniliasis. *Oral Surg Oral Med Oral Pathol*. (1970) 30:356–65.

140. Castanier J, Hee P, Lelievre-Gassin C, Brulliard M. Deux cas d'hypoparathyroïdie dont un avec candidose cutanéo-muqueuse chronique. *Rev Pediatr*. (1970) 6:403–8.

141. Ferchiou A, Valencogne A, Denguezli, Castaing H, Snoussi N, Younes B. Á propos d'une tétanie (une hypoparathyroïdie chronique idiopathique de l'enfant). *Tunis Med*. (1970) 48:9–15.

142. Irvine WJ. Autoimmune ovarian failure. In: Irvine WJ, editor. *Reproductive endocrinology*. Edinburgh, UK: E. and S. Livingstone. (1970). p. 106–15.

143. Kolb FO, Irvine WJ, Steinbach HL, Goldman L, Webb G. Primary hypoparathyroidism, Addison's disease and ovarian failure. In: Irvine WJ, editor. *Reproductive endocrinology*. Edinburgh, UK: E. and S. Livingstone. (1970). p. 116–21.

144. Block MB, Pachman LM, Windhorst D, Goldfine ID. Immunological findings in familial juvenile endocrine deficiency syndrome associated with mucocutaneous candidiasis. *Am J Med Sci*. (1971) 261:213–8.

145. Kirkpatrick CH, Rich RR, Bennett JE. Chronic mucocutaneous candidiasis: model-building in

cellular immunity. *Ann Intern Med*. (1971) 74:955–78. doi: 10.7326/0003-4819-74-6-955

146. Fields JP, Fragola L, Hadley TP. Hypoparathyroidism, candidiasis, alopecia and vitiligo. *Arch Dermatol*. (1971) 103:687–9. doi: 10.1001/archderm.103.6.687

147. Mariani R, Maurel P, Combres J-C, Giraud F, Bernard R. Hypoparathyroïdie et insuffisance surrénale familiale. *Arch Fr Pediatr*. (1971) 28:447–8.

148. Blizzard RM. Idiopathic hypoparathyroidism and Addison's disease without moniliasis. *Birth Defects Orig Artic Ser*. (1971) 7:266–7.

149. Fárková H. Idiopatický hypoparatyreoidismus. *Cesk Pediatr*. (1971) 26:280–2.

150. Axelsen NH. Human precipitins against a micro-organism (*Candida albicans*) demonstrated by means of quantitative immunoelectrophoresis. *Clin Exp Immunol*. (1971) 9:749–52.

151. Money J, Bobrow NA, Clarke FC. Autism and autoimmune disease: a family study. *J Autism Child Schizophr*. (1971) 1:146–60. doi: 10.1007/BF01537954

152. Castells S, Fikrig S, Inamdar S, Orti E. Familial moniliasis, defective delayed hypersensitivity, and adrenocorticotropic hormone deficiency. *J Pediatr*. (1971) 79:72–9. doi: 10.1016/s0022-3476(71)80061-6

153. Moulias R, Goust JM, Muller-Berat CN. Hypoparathyroidism and cell-mediated immunity. *Lancet*. (1971) 297:1239. doi: 10.1016/s0140-6736(71)91750-8

154. Levy RL, Bach ML, Huang S-W, Bach FH, Hong R, Ammann AJ, et al. Thymic transplantation in a case of chronic mucocutaneous candidiasis. *Lancet*. (1971) 298:898–900. doi: 10.1016/s0140-6736(71)92503-7

155. Moulias R, Goust J-M, Berthaux P. Les phénomènes immunitaires dans les maladies endocriennes. *Rev Prat*. (1971) 21:3325–34.

156. Kössling FK, Emmrich P. Demonstration eines Falles von kindlichem Morbus Addison mit Hypoparathyreoidismus. *Verh Dtsch Ges Pathol*. (1971) 55:155–60.

157. de Moraes Ruehsen M, Blizzard RM, Garcia-Bunuel R, Jones GS. Autoimmunity and ovarian failure. *Am J Obstet Gynecol*. (1972) 112:693–703. doi: 10.1016/0002-9378(72)90797-1

158. Stankler L, Bewsher PD. Chronic mucocutaneous candidiasis, endocrine deficiency and alopecia areata. *Br J Dermatol*. (1972) 86:238–45. doi: 10.1111/j.1365-2133.1972.tb02223.x

159. Higgs JM, Wells RS. Chronic muco-cutaneous candidiasis: associated abnormalities of iron metabolism. *Br J Dermatol*. (1972) 86(S8):88–102. doi: 10.1111/j.1365-2133.1972.tb15420.x

160. Témime P. Les manifestations cutanées dentaires et unguéales rencontrées dans l'hypoparathyroïdisme idiopathique. *Bull Soc Fr Dermatol Syphiligr*. (1972) 79:293.

161. Irvine WJ, Barnes EW. Adrenocortical insufficiency. *Clin Endocrinol Metab*. (1972) 1:549–94. doi: 10.1016/S0300-595X(72)80030-6

162. Perheentupa J. Kaksi ryhmää lisämunuaiskuoren periytyviä vajaatoimintatiloja. *Duodecim*. (1972) 88:119–27.

163. Lehner T, Wilton JMA, Ivanyi L. Immunodeficiencies in chronic muco-cutaneous candidosis. *Immunology*. (1972) 22:775–87.

164. Wells RS, Higgs JM, Macdonald A, Valdimarsson H, Holt PJL. Familial chronic muco-cutaneous candidiasis. *J Med Genet*. (1972) 9:302–10. doi: 10.1136/jmg.9.3.302

165. Olin R, Poindexter MH. Familial idiopathic hypoparathyroidism with superficial moniliasis, pernicious anemia and Addison's disease. *Minn Med*. (1972) 55:701–4.

166. Rameis K, Kurz R, Glatzl J. Beitrag zum Syndrom Moniliasis, Hypoparathyreoismus und Morbus Addison. *Padiatr Padol*. (1972) 7:279–87.

167. Breynaert R, Bergeron G, Dumortier-Iscovich C, Pieraert C, Lefebvre J, Farriaux JP, et al. Une observation familiale de syndrome de Whitaker. *Acta Paediatr Belg*. (1973) 27:184–217.

168. Perheentupa J, Hiekkala H. Twenty cases of the syndrome of autoimmune endocrinopathy and candidiasis. *Acta Paediatr Scand*. (1973) 62:110–1. doi: 10.1111/j.1651-2227.1973.tb08074.x

169. Valdimarsson H, Higgs JM, Wells RS, Yamamura M, Hobbs JR, Holt PJL. Immune abnormalities associated with chronic mucocutaneous candidiasis. *Cell Immunol*. (1973) 6:348–61. doi: 10.1016/0008-8749(73)90035-x

170. Mitsckhe H, Altenähr E, Delling G, Wiebel J. Das pluriglanduläre Insuffizienzsyndrom. Hypoparathyreoidismus – Morbus Addison – Moniliasis. *Dtsch Med Wochenschr*. (1973) 98:1666–9. doi: 10.1055/s-0028-1107105

171. Arvanitakis C, Knouss RF. Selective hypopituitarism. Impaired cell-mediated immunity and chronic mucocutaneous candidiasis. *JAMA*. (1973) 225:1492–5. doi: 10.1001/jama.225.12.1492

172. Scott ME. Resistant hypoparathyroidism and magnesium depletion. *Lancet*. (1973) 301:1005. doi: 10.1016/s0140-6736(73)91653-x

173. Hooper MJ, Carter JN, Stiel JN. Idiopathic hypoparathyroidism and idiopathic hypoadrenalism occurring separately in two siblings. *Med J Aust*. (1973) 1:990–3. doi: 10.5694/j.1326-5377.1973.tb110856.x

174. Foz M, Mirada A, Guardia J, Tresánchez JM. Enfermedad de Addison familiar asociada con hipoparatiroidismo. *Med Clin (Barc)*. (1973) 60:318–21.

175. Corvaglia E. L'ipoparatiroidismo cronico idiopatico familiare nell'infanzia. Descrizione di due casi inquadrabili nella sindrome di Whitaker. *Minerva Pediatr*. (1973) 25:104–19.

176. Vazquez AM, Kenny FM. Ovarian failure and antiovarian antibodies in association with hypoparathyroidism, moniliasis, and Addison's and Hashimoto's diseases. *Obstet Gynecol*. (1973) 41:414–8.

177. Krohn K, Heinonen E, Pelkonen R, Perheentupa J. Precipitating antiadrenal antibodies in Addison's disease. *Scand J Immunol*. (1973) 2:450. doi: 10.1111/j.1365-3083.1973.tb02053.x

178. Wong VG, Kirkpatrick CH. Immunologic reconstitution in a patient with keratoconjunctivitis, superficial candidiasis and hypoparathyroidism: the role of immunocompetent lymphocyte transfusion and transfer factor. *Trans Am Ophthalmol Soc*. (1973) 71:254–71.

179. Dumortier-Iscovich C (1973). Hypoparathyroïdie chronique insuffisance surrénalienne et candidose. Etude chez deux frères. *Dissertation Thesis*. University of Lille, France.

180. Kamp P, Platz P, Nerup J. "Steroid-cell" antibody in endocrine diseases. *Acta Endocrinol. (Copenh)*. (1974) 76:729–40. doi: 10.1530/acta.0.0760729

181. Langslet A, Olsen I, Lie SO, Løkken P. Chlorhexidine treatment of oral candidiasis in seriously diseased children. *Acta Paediatr Scand*. (1974) 63:809–11. doi: 10.1111/j.1651-2227.1974.tb04868.x

182. Marieb NJ, Melby JC, Lyall SS. Isolated hypoaldosteronism associated with idiopathic

hypoparathyroidism. *Arch Intern Med*. (1974) 134:424–9. doi: 10.1001/archinte.1974.00320210034004

183. Kleerekoper M, Basten A, Penny R, Posen S. Idiopathic hypoparathyroidism with primary ovarian failure. Report of a case with detailed immunological studies. *Arch Intern Med*. (1974) 134:944–7. doi: 10.1001/archinte.1974.00320230154028

184. Krohn K, Perheentupa J, Heinonen E. Precipitating anti-adrenal antibodies in Addison's disease. *Clin Immunol Immunopathol*. (1974) 3:59–68. doi: 10.1016/0090-1229(74)90023-3

185. Conte N, Del Prete GF, Betterle C, Bottazzo GF, Trisotto A. Familial chronic idiopathic hypoparathyroidism associated with rheumatoid arthritis. *Folia Allergol Immunol Clin*. (1974) 21:482–91.

186. Jehanne M, Guivarch J. Hypoparathyroïdie chronique idiopathique avec insuffisance ovarienne immunologique. *Ouest Med*. (1974) 27:313–9.

187. L'vovskaia SS. Tsirkuliruiushchie antitela pri addisonovoi bolezni. *Probl Endokrinol (Mosk)*. (1974) 20:44–6.

188. Bovier-Lapierre M, Jeune M. La maladie d'Addison chez l'enfant. Á propos de 9 observations personelles. In: Journées Parisiennes de Pédiatrie, editor. *L'infection urinaire récidivante de l'enfant. Les insuffisances surrénales chroniques du nourrisson et de l'enfant. Paris, 21–22 Septembre 1974*. Paris, France: Flammarion Médecine-Sciences. (1974). p. 127–46.

189. Del Prete GF, Bottazzo GF, Betterle C, Trisotto A. Hypoparathyroïdisme chronique, insuffisance surrénale et ovarienne, gastrite atrophique, goitre, diabète chimique et moniliase. *Ann Endocrinol (Paris)*. (1975) 36:95–6.

190. Schulkind ML, Ayoub EM. Transfer factor as an approach to the treatment of immune deficiency disease. *Birth Defects Orig Artic Ser*. (1975) 11:436–40.

191. Kaffe S, Petigrow CS, Cahill LT, Perlman D, Moloshok RE, Hirschhorn K, et al. Variable cell-mediated immune defects in a family with "*Candida* endocrinopathy syndrome". *Clin Exp Immunol*. (1975) 20:397–408.

192. McNatty KP, Short RV, Barnes EW, Irvine WJ. The cytotoxic effect of serum from patients with Addison's disease and autoimmune ovarian failure of human granulosa cells in culture. *Clin Exp Immunol*. (1975) 22:378–84.

193. Irvine WJ, Barnes EW. Addison's disease, ovarian failure and hypoparathyroidism. *Clin Endocrinol Metab*. (1975) 4:379–434. doi: 10.1016/S0300-595X(75)80027-2

194. Del Prete GF, Bottazzo GF, Betterle C, Bersani G, Trisotto A. Poliendocrinopatia autoimmune. Studio immunologico. *Folia Allergol Immunol Clin*. (1975) 22:123–9.

195. Mackey JP. Endocrine candidosis syndrome. *Ir J Med Sci*. (1975) 144:44–5. doi: 10.1007/BF02938988

196. Bottazzo GF, Pouplard A, Florin-Christensen A, Doniach D. Autoantibodies to prolactin-secreting cells of human pituitary. *Lancet*. (1975) 306:97–101. doi: 10.1016/s0140-6736(75)90004-5

197. Petković V. Sindrom keratokonjunktivitisa, površne moniliaze i Addisonove bolesti. *Rad Med Fak Zagrebu*. (1975) 23:77–83.

198. Joksimović I, Banićević M, Vulović D, Kezić J, Mikuška M, Filipović D, et al. Sindrom kandidijaza – hipoparatireoidizam – Adisonova bolest. *Srp Arh Celok Lek*. (1975) 103:1–10.

199. Brun J-M (1975). Polyendocrinopathies juvéniles auto-immunes et affections associées.

Dissertation Thesis. University of Dijon, France.

200. Champenois J-P (1975). Étude des anomalies dentaires dans l’hypoparathyroïdie idiopathique précoce. *Dissertation Thesis*. University of Lille, France.

201. Weinberg U, Kraemer FB, Kammerman S. Coexistence of primary endocrine deficiencies: a unique case of male hypergonadism associated with hypoparathyroidism, hypoadrenocorticism, and hypothyroidism. *Am J Med Sci*. (1976) 272:215–20.

202. Heinonen E, Krohn K, Perheentupa J, Aro A, Pelkonen R. Association of precipitating anti-adrenal antibodies with moniliasis-polyendocrinopathy syndrome. *Ann Clin Res*. (1976) 8:262–5.

203. Lawton JWM, Costello C, Barclay GR, Urbaniak SJ, Darg C, Raeburn JA, et al. The effect of transfer factor on neutrophil function in chronic mucocutaneous candidiasis. *Br J Haematol*. (1976) 33:137–42. doi: 10.1111/j.1365-2141.1976.tb00980.x

204. Takeya K, Nomoto K, Matsumoto T, Miyake T, Himeno K. Chronic mucocutaneous candidiasis accompanied by enhanced antibody production. *Clin Exp Immunol*. (1976) 25:497–500.

205. Chesney RW, O’Regan S, Guyda HJ, Drummond KN. Candida endocrinopathy syndrome with membranoproliferative glomerulonephritis: demonstration of glomerular candida antigen. *Clin Nephrol*. (1976) 5:232–8.

206. Farrell PM, Rikkers H, Moel D. Cortisol-dihydrotachysterol antagonism in a patient with hypoparathyroidism and adrenal insufficiency: apparent inhibition of bone resorption. *J Clin Endocrinol Metab*. (1976) 42:953–7. doi: 10.1210/jcem-42-5-953

207. Heinonen E. Variety of determinants in an adrenal antigen common to man and some animals. *Med Biol*. (1976) 54:341–6.

208. Griscelli C, Herzog C, Durandy A, Fischer A, Mozziconacci P. Étude immunologique des candidoses chroniques de l’enfant. *Rev Fr Allergol Immunol Clin*. (1976) 16:257–63.

209. Blake J. Eye signs in idiopathic hypoparathyroidism. *Trans Ophthalmol Soc U K*. (1976) 96:448–51.

210. Vayssettes-Vernet A-M (1976). Association hypoparathyroïdie chronique – insuffisance cortico-surrénalienne – candidose chez l’enfant (Syndrome de Whitaker). À propos de 3 observations. Revue de la littérature. *Dissertation Thesis*. University of Grenoble, France.

211. Stieglitz LN, Kind HP, Kazdan JJ, Fraser D, Kooh SW. Keratitis with hypoparathyroidism. *Am J Ophthalmol*. (1977) 84:467–72. doi: 10.1016/0002-9394(77)90436-6

212. Okamoto GA, Hall JG, Ochs H, Jackson C, Rodaway K, Chandler J. New syndrome of chronic mucocutaneous candidiasis. *Birth Defects Orig Artic Ser*. (1977) 13:117–25.

213. Del Prete GF, Betterle C, Padovan D, Erle G, Toffolo A, Bersahi G. Incidence and significance of islet-cell autoantibodies in different types of diabetes mellitus. *Diabetes*. (1977) 26:909–15. doi: 10.2337/diab.26.10.909

214. Tiengo A, Del Prete GF, Nosadini R, Betterle C, Garotti C, Bersani G. Insulin and glucagon secretion in diabetic and non-diabetic patients with circulating islet cell antibodies. *Diabetologia*. (1977) 13:451–8. doi: 10.1007/BF01234495

215. Lucky AW, Rebar RW, Blizzard RM, Goren EM. Pubertal progression in the presence of elevated serum gonadotropins in girls with multiple endocrine deficiencies. *J Clin Endocrinol Metab*. (1977) 45:673–8. doi: 10.1210/jcem-45-4-673

216. Heinonen E, Krohn K. Studies on an adrenal antigen common to man and different animals. *Med*

Biol. (1977) 55:48–53.

217. Hertz KC, Gazze LA, Kirkpatrick CH, Katz SI. Autoimmune vitiligo. Detection of antibodies to melanin-producing cells. *N Engl J Med*. (1977) 297:634–7. doi: 10.1056/NEJM197709222971204

218. Chesney RW, Horowitz SD, Kream BE, Eisman JA, Hong R, De Luca HF. Failure of conventional doses of 1α,25-dihydroxycholecalciferol to correct hypocalcemia in a girl with idiopathic hypoparathyroidism. *N Engl J Med*. (1977) 297:1272–5. doi: 10.1056/NEJM197712082972310

219. Pisanty S, Garfunkel A. Familial hypoparathyroidism with candidiasis and mental retardation. *Oral Surg Oral Med Oral Pathol*. (1977) 44:374–83. doi: 10.1016/0030-4220(77)90407-8

220. Rebollar JL, Prieto S, Perez Gutierrez A, Gilsanz FJ, Amarillas L. Hipoparatiroidismo primario: a propósito de tres casos. *Rev Clin Esp (Barc)*. (1977) 147:207–10.

221. Miani C, Rumi G. Candidosi del cavo orale, ipoparatiroidismo, iposurrenalismo associati a gravi alterazioni dentarie. *Riv Ital Stomatol*. (1977) 46:4–9.

222. Brun J-M. Les polyendocrinopathies juvéniles auto-immunes. *Ann Endocrinol (Paris)*. (1978) 39:463–81.

223. Cortet P, Brun J-M, Rifle C, Seigneuric C. Candidose, hypoparathyroïdie chronique, insuffisance surrénale, insuffisance ovarienne primitive et maladie de Biermer. Extension de la triade de Whitaker. *Ann Med Interne (Paris)*. (1978) 129:449–53.

224. Matsumoto T. Chronic mucocutaneous candidiasis and transfer factor. *Nihon Ishinkin Gakkai Zasshi*. (1978) 19:214–20.

225. Bottazzo GF, Doniach D. Pituitary autoimmunity: a review. *J R Soc Med*. (1978) 71:433–6.

226. Dučić V, Terzić-Kazić N. Sindrom kandida-endokrinopatija. *Med Arh*. (1978) 32:155–8.

227. Irvine WJ. Autoimmunity against steroid producing organs. *Menarini Ser Immunopathol*. (1978) 1:35–49.

228. Bottazzo GF, Doniach D. The detection of autoantibodies to discrete cells in anterior pituitary and pancreatic islets. *Menarini Ser Immunopathol*. (1978) 1:50–62.

229. Prader A. Syndrome mit besonderer Beteiligung des Endokriniums. *Monatsschr Kinderheilkd*. (1978) 126:264–8.

230. Myllärniemi S, Perheentupa J. Oral findings in the autoimmune polyendocrinopathy-candidosis syndrome (APECS) and other forms of hypoparathyroidism. *Oral Surg Oral Med Oral Pathol*. (1978) 45:721–9. doi: 10.1016/0030-4220(78)90147-0

231. Perheentupa J, Tiilikainen A, Lokki M-L. Autoimmune polyendocrinopathy-candidosis syndrome (APECS): clinical variation, inheritance and HLA association in 40 Finnish patients. *Pediatr Res*. (1978) 12:1087. doi: 10.1203/00006450-197811000-00038

232. Brenton DP, Gonzales J, Pollard AB. Hypocalcaemic cardiac failure. *Postgrad Med J*. (1978) 54:633–6. doi: 10.1136/pgmj.54.635.633

233. Irvine WJ. Adrenalitis, hypoparathyroidism and associated diseases. In: M. Samter, editor. *Immunological Diseases. Third Edition*. Boston (MA), USA: Little, Brown and Company. (1978). p. 1278–95.

234. Tomar RH, Rao RJ, Lawrence A, Moses AM. Moniliasis and anergy in hypoparathyroidism: treatment with transfer factor. *Ann Allergy*. (1979) 42:241–5.

235. Eisenbarth GS, Wilson PW, Ward F, Buckley C, Lebovitz H. The polyglandular failure syndrome: disease inheritance, HLA type, and immune function. Studies in patients and families. *Ann Intern Med.* (1979) 91:528–33. doi: 10.7326/0003-4819-91-4-528

236. Betterle C, Peserico A, Bersani G. Vitiligo and autoimmune polyendocrine deficiencies with autoantibodies to melanin producing cells. *Arch Dermatol.* (1979) 115:364. doi: 10.1001/archderm.1979.04010030066028

237. Matzner Y, Rubinger D, Eldor A. Hoser-dam mam'ir, halbanat hase'ar wehafra'ot holfot be-mangnon ha-hisuni uvatifqud hatuha etzel hola tat-yoteret-trisiyyut rishonit. *Harefuah.* (1979) 96:104–5.

238. Garfunkel AA, Pisanty S, Michaeli Y. Familial hypoparathyroidism, candidiasis and mental retardation – a histopathologic study of the dental structures. *J Oral Med.* (1979) 34:13–7.

239. Arulanantham K, Dwyer JM, Genel M. Evidence for defective immunoregulation in the syndrome of familial candidiasis endocrinopathy. *N Engl J Med.* (1979) 300:164–8. doi: 10.1056/NEJM197901253000403

240. Ivanova MA, Ignatovich TP. Idiopaticheskii gipoparatireoz u rebenka 9 let. *Pediatriia.* (1979) 3:60–1.

241. Myers TJ, Bower BF, Hild DH. Pure red cell aplasia and the syndrome of multiple endocrine gland insufficiency. *Am J Med Sci.* (1980) 280:29–34. doi: 10.1097/00000441-198007000-00005

242. Adami Lami C, Calandi C, De Martino M, Redi Orienti M, Vierucci A. Alterazioni immunologiche in un caso di sindrome di Whitaker. *Ann Sclavo.* (1980) 22:640–53.

243. Clifton-Bligh P, Lee C, Smith H, Posen S. The association of diabetes insipidus with hypoparathyroidism, Addison's disease and mucocutaneous candidiasis. *Aust N Z J Med.* (1980) 10:548–51. doi: 10.1111/j.1445-5994.1980.tb04974.x

244. Kessel LJ, Taylor WD. Chronic mucocutaneous candidiasis – treatment of the oral lesions with miconazole: two case reports. *Br J Oral Surg.* (1980) 18:51–6. doi: 10.1016/0007-117x(80)90052-9

245. Sotsiou F, Bottazzo GF, Doniach D. Immunofluorescence studies on autoantibodies to steroid-producing cells, and to germline cells in endocrine disease and infertility. *Clin Exp Immunol.* (1980) 39:97–111.

246. Betterle C, Caretto A, Tiengo A, Trevisan A. Complement-fixing islet-cell antibodies in type I diabetes and in susceptible patients with autoimmune diseases. *Lancet.* (1980) 315:1418–9. doi: 10.1016/s0140-6736(80)92685-9

247. Neufeld M, Maclaren N, Blizzard R. Autoimmune polyglandular syndromes. *Pediatr Ann.* (1980) 9:154–62.

248. Neufeld M, Blizzard RM. Polyglandular autoimmune disease. *Proc Serono Symp.* (1980) 33:357–65.

249. Bottazzo GF, Vandelli C, Mirakian R. The detection of autoantibodies to discrete endocrine cells in complex endocrine organs. *Proc Serono Symp.* (1980) 33:367–77.

250. Brun JM, Cortet P. Hypoparathyroidism and chronic candidiasis with multiple endocrine deficiencies in a child: a "juvenile autoimmune polyendocrine syndrome". *Proc Serono Symp.* (1980) 33:379–84.

251. Rico Zalba LA, Calderin FM. Síndrome de endocrinopatía familiar con candidiasis. *Rev Clin Esp.* (1980) 157:205–7.

252. Sapelkina LV, Skripkina VM, Tsyvilskaya LA, Demidova LV, Tint EG, Kravchenko AI. Gipoparatireoz u detei. *Vopr Okhr Materin Det*. (1980) 25:16–20.

253. Perheentupa J. Autoimmune polyendocrinopathy – candidosis – ectodermal dystrophy (APECED). In: Eriksson AW, Forsius HR, Nevanlinna HR, Workman PL, Norio RK, editors. *Population structure and genetic disorders*. London, UK: Academic Press. (1980). p. 583–7.
